# Bromine–lithium exchange: An efficient tool in the modular construction of biaryl ligands

**DOI:** 10.3762/bjoc.7.148

**Published:** 2011-09-14

**Authors:** Laurence Bonnafoux, Frédéric R Leroux, Françoise Colobert

**Affiliations:** 1 Laboratoire de stéréochimie, UMR 7509, CNRS-Université de Strasbourg, ECPM, 25 rue Becquerel, F-67087 Strasbourg Cedex 02, France

**Keywords:** biaryl, bromine–lithium exchange, ligand, lithiation, phosphine

## Abstract

Regioselective bromine–lithium exchange reactions on polybrominated biaryls enable the modular synthesis of various polysubstituted biphenyls such as bis(dialkylphosphino)-, bis(diarylphosphino)- and dialkyl(diaryl)phosphinobiphenyls. All permutations of substituents at the ortho positions of the biphenyls are possible. In a similar manner, one can gain access to monophosphine analogues. So far, such a process, based on the effective discrimination between bromine atoms as a function of their chemical environment, has been observed only sporadically.

## Introduction

Atropisomeric biaryls are important compounds in various fields. In particular, pharmaceuticals and agrochemicals with biaryl substructures are of general interest [1]. In addition, they have widespread applications as ligands in catalysis, or in materials sciences [2]. The atropisomeric *C*_2_-symmetric binaphthyl- or biphenyl-bridged diphosphine ligands BINAP [3], H8-BINAP [4], BIPHEMP [5], MeO-BIPHEP [6], SEGPHOS [7–9], P-Phos [10], SYNPHOS [11–12], C*_n_*-TUNAPHOS [13] and DIFLUORPHOS [14–16] and their analogues are well known as highly efficient chiral ligands for a variety of transition metal-catalyzed asymmetric transformations. The biphenyl backbone has the advantage that substituents at the 6- and 6'-positions can affect the dihedral angle of the biphenyl backbone, one of the key factors for ligand efficiency. Both the aryl phosphorus substituents and the biphenyl backbone can be tailored in order to modify the stereoelectronic profile of a ligand.

Most frequently, biaryls are prepared through transition metal-catalyzed reactions of suitable functionalized starting materials [17–22]. Although these methods are well established, alternatives are investigated in order to avoid expensive transition metals or ligands, which are especially required for the coupling of deactivated or sterically hindered substrates.

Our group is developing new methods towards the synthesis of highly functionalized atropisomeric biphenyls [23–32]. We seek to perform their synthesis (a) in a modular way starting from a few common and easily available precursors; (b) with a high degree of structural diversity; (c) in a straightforward, short, reproducible manner; (d) in high yield and on multigram scale; and, last but not least, (e) with a restricted use of transition metals and ligands.

Polar organometallic chemistry [33–35] allows the performance of highly selective reactions. Therefore, it seemed to us the ideal tool to reaching this goal. In this context, we recently developed a novel transition metal-free aryl–aryl coupling protocol, the "ARYNE-coupling", which allows the preparation of di-, tri-, and even tetra-substituted *ortho*,*ortho*'-dibromobiphenyls [25,28,31,36–38]. These have the advantage that, by means of successive or simultaneous bromine–lithium exchanges, a huge panel of substituents can be introduced into the biphenyl backbone.

The bromine–lithium exchange reaction is certainly one of the most fundamental synthetic transformations [39]. Although this reaction was reported by C. S. Marvel in 1927 [40], G. Wittig [41] and H. Gilman [42–44] were the first to apply it in organic synthesis in the late thirties. Since then, this reaction has been considered as a mature method lacking both appeal and surprise [33], and only new applications of this reaction or mechanistic studies have been reported [30,32–33,45–55]. However, in the last few years, the halogen–metal permutation has recaptured its former role as one of the most important and versatile methods in organic synthesis. New exchange reagents, such as isopropylmagnesium chloride, its LiCl complex [53–61] and lithium tributylmagnesate [62–63], have been developed and allow reactions under noncryogenic conditions [64–66]. New access routes to synthetically challenging aryl halide precursors have been devised.

A. Alexakis et al. recently achieved a significant breakthrough*.* They succeeded in the desymmetrization of prochiral polybrominated [32,51] compounds by an asymmetric bromine–lithium exchange in the presence of a stoichiometric amount of chiral diamines. An enantiomeric excess of up to 63% was obtained [67]. H. Kagan et al. reported the desymmetrization of prochiral aromatic or vinylic dihalide substrates by halogen–metal exchange in the presence of a stoichiometric amount of diamines, with enantiomeric excess up to 26% [68]. Very recently, the Alexakis group achieved the catalytic bromine–lithium exchange allowing the preparation of biarylatropisomers in quantitative yields and enantiomeric excesses up to 82% [69].

**Figure 1:**
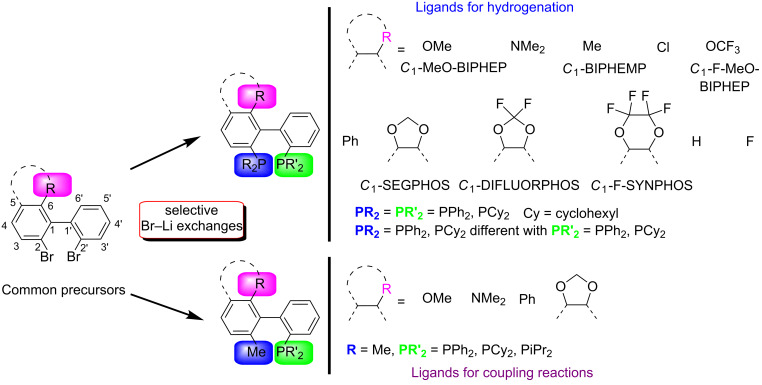
Modular synthesis of bis(diarylphosphino)-, bis(dialkylphosphino)- and dialkyl(diaryl)phosphinobiphenyls as well as monophosphinobiphenyls by means of polar organometallic chemistry.

Herein, we report on the preparation of *C*_1_ analogues of the most efficient and popular *C*_2_-symmetric biphenyl ligands. We will show that by means of regioselective bromine–lithium exchanges all possible permutations of bis(diaryldiphosphino)-, bis(dialkylphosphino)- and dialkyl(diaryl)phosphinobiphenyls become feasible. In a similar way, biphenyl-based monophosphine ligands were also obtained (Figure 1).

## Results and Discussion

### Regioselective bromine–lithium exchange on polybrominated biphenyls

**Figure 2:**
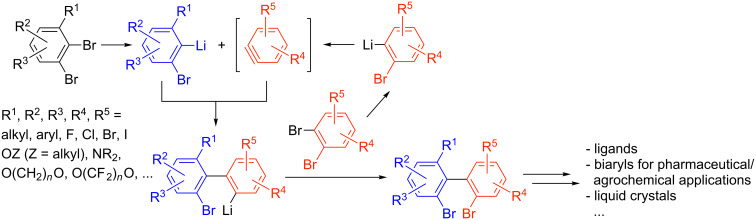
ARYNE coupling.

Our group recently reported the efficient coupling of organolithium intermediates with arynes, the so-called "ARYNE coupling" [25,31,36]. This protocol is based on the formation of a thermodynamically stable aryllithium intermediate and its subsequent reaction with a 1,2-dibromobenzene derivative. The transient benzyne adds the aryllithium derivative, followed by in situ transfer of bromine between the resulting 2-biaryllithium intermediate and another molecule of 1,2-dibromobenzene. Mono-, di- or even tetra-substituted *ortho*-bromobiaryls can be obtained on a gram scale (Figure 2).

The intriguing question is whether one bromine would be exchanged preferentially on the substrate when the bromine atoms are not activated by adjacent heteroatoms. An effective discrimination between two bromine atoms as a function of their chemical environment has so far been observed only sporadically in such processes [30,34–35,51–52,70–72].

**Scheme 1:**
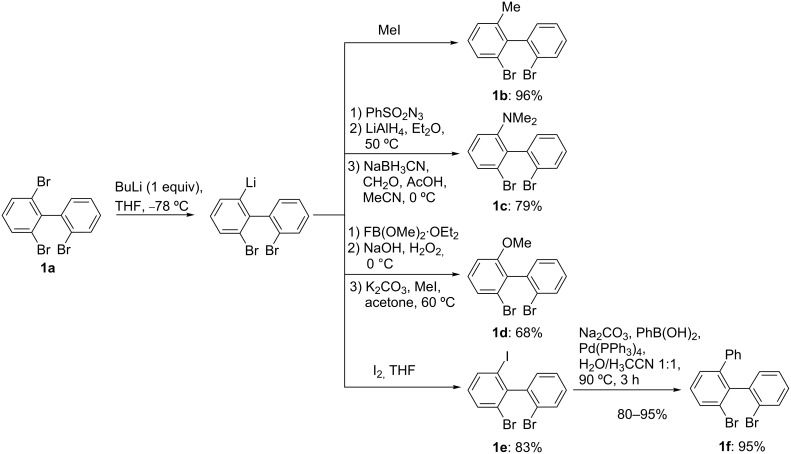
Functionalization of 2,2',6-tribromobiphenyl (**1a**) by regioselective bromine–lithium exchange.

Fortunately, the reaction occurred exclusively on the doubly halogenated ring when 2,2',6-tribromobiphenyl [28,38], obtained almost quantitatively by the ARYNE coupling protocol, was submitted to the bromine–lithium exchange reaction. When 2,2',6-tribromobiphenyl (**1a**) [28] was treated at −78 °C with BuLi and the intermediate aryllithium trapped with iodomethane, 2,2'-dibromo-6-methylbiphenyl (**1b**) was obtained in an excellent yield of 96%. Analogously, when benzenesulfonylazide was used as an electrophile, 2-azido-2',6-dibromobiphenyl was obtained. The use of lithium aluminium hydride in ether at reflux for 4.5 h gave exclusively 2-amino-2',6-dibromobiphenyl, which was submitted to a reductive methylation by means of formaldehyde and sodium cyanoborohydride. 2-*N*,*N*-Dimethylamino-2',6-dibromobiphenyl (**1c**) was obtained in an overall yield of 79% in 3 steps (Scheme 1). To introduce the methoxy group, 2,2',6-tribromobiphenyl (**1a**) was successively subjected to lithiation, borylation with fluorodimethoxyborane·diethyl ether, followed by oxidation with hydrogen peroxide and *O*-methylation with iodomethane in acetone. 2,2'-Dibromo-6-methoxybiphenyl (**1d**) was finally obtained in a very good global yield of 68% in 3 steps. Finally, we proposed to introduce the phenyl ring (**1f**, 95%) by a regioselective Suzuki–Miyaura coupling via the iodo derivative **1e**, the latter being obtained in 83% yield after trapping with iodine.

**Scheme 2:**
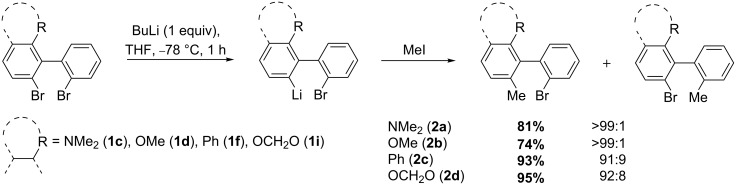
Functionalization of 2,2'-dibromobiphenyls (**1b–e**) by regioselective bromine–lithium exchange.

Similarly, as shown for 2,2',6-tribromobiphenyl (**1a**), when 6-substituted 2,2'-dibromobiphenyls **1b–e** were treated with just one equivalent of butyllithium in tetrahydrofuran at −78 °C, another regioselective bromine–lithium exchange occurs on the functionalized ring (Scheme 2). Trapping with iodomethane afforded the biphenyls **2** in high yield and perfect regioselectivity, except for R = Ph (**1f**) and the benzodioxole derivative (**1i**), where the regioselectivity was slightly lower (91:9 and 92:8, respectively).

M. Schlosser and J. Gorecka-Kobylinska recently reported on the relative basicities of aryllithiums bearing methoxy, chlorine, fluorine, trifluoromethyl and trifluoromethoxy substituents at the ortho, meta, and para positions. Equilibration studies of two aryllithiums of comparable basicity with the corresponding bromo- or iodoarenes allowed them to determine the "basicity" (protodelithiation) increments ∆∆*G*, derived from the equilibrium constants. The authors showed that the basicity increments are linearly correlated with the relative protonation enthalpies of the corresponding aryl anions in the gas phase. Compared with "naked" aryl anions, the basicity of aryllithiums mirrors the effects of ortho, meta, and para substituents to the extent of 36%, 30%, and 25%, respectively [73].

These results explain the difference in regioselectivity of the bromine–lithium exchange, between a bromine atom residing on a phenyl ring that bears a "stabilizing" substituent at a remote meta position and a bromine atom on an "unstabilized" phenyl ring.

### Biaryl mono- and diphosphines

**Figure 3:**
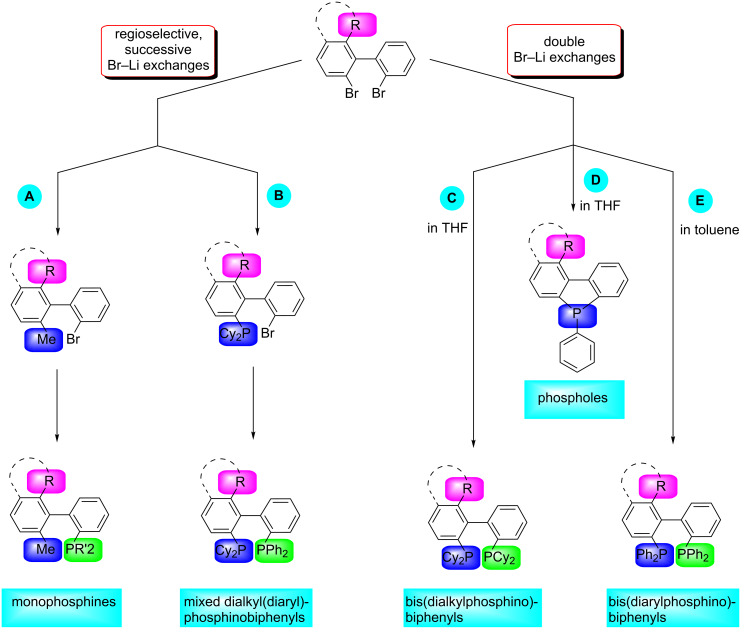
General access to biaryl mono- and diphosphine ligands; (Cy = cyclohexyl).

In the following section we will show how a large family of biaryl mono- and diphosphines becomes readily accessible through these common building-blocks. The general access is depicted in Figure 3.

### Biarylmonophosphines: Path A

**Scheme 3:**
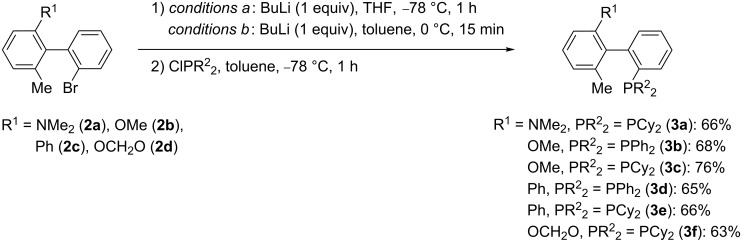
Synthesis of monophosphines **3**; (Cy = cyclohexyl).

From the methylated intermediates **2a–d**, new monophosphines became accessible in one additional step (Scheme 3). The bromine–lithium exchange was either performed with just one equivalent of butyllithium in tetrahydrofuran at −78 °C (*conditions a*) or in toluene at 0 °C (*conditions b*). After cooling to −78 °C, the lithiated intermediate was then allowed to react with a solution of ClPCy_2_, or ClPPh_2_ in toluene. In these cases, the monophosphines **3** were obtained in good yields (Scheme 3).

**Figure 4:**
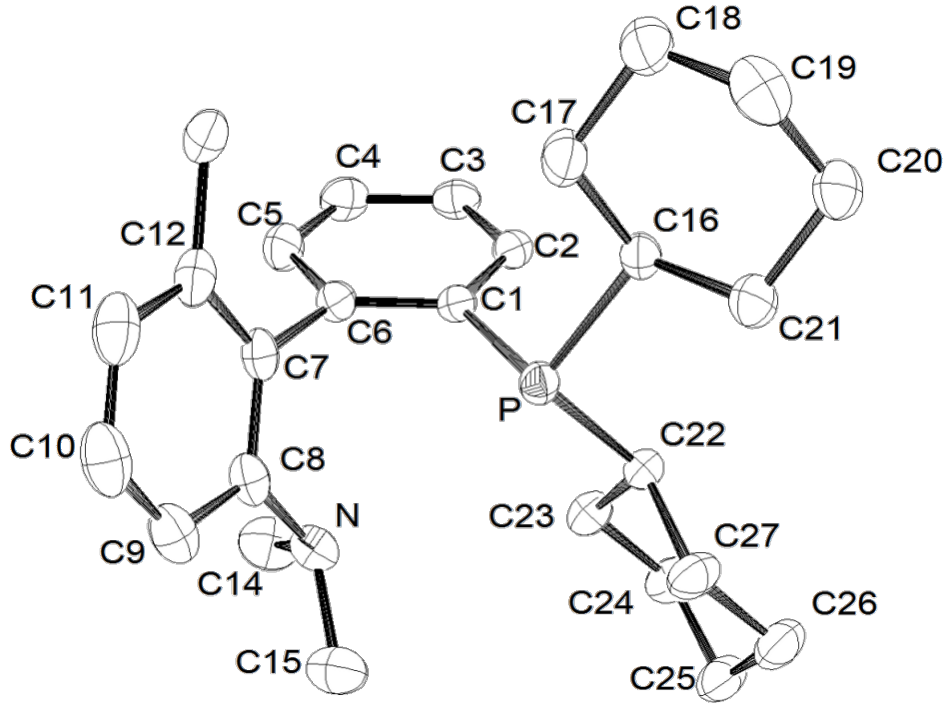
Molecular structure of compound **3a** (crystallized from ethyl acetate/hexane) [74].

Single crystal X-ray analyses [74] of one ligand of each family (R^1^ = NMe_2_ (**3a**, Figure 4), OMe (**3b**, see Supporting Information File 1), Ph (**3d**, see Supporting Information File 2), OCH_2_O (**3f**, see Supporting Information File 3)) were performed in order to confirm their structure. Thus, we confirmed the selectivity of the different halogen–metal exchange reactions performed throughout the synthesis.

### Mixed dialkyl(diaryl)phosphinobiphenyls: Path B

**Scheme 4:**
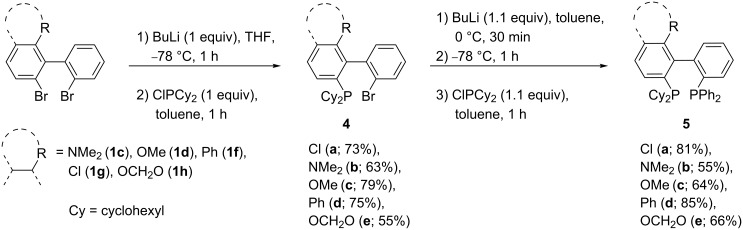
Preparation of mixed dialkyl(diaryl)phosphinobiphenyls **5** via successive bromine–lithium exchange.

In order to synthesize mixed diphosphines, we took advantage of the non-equivalence of the two phenyl rings towards lithiation. We started from the same dibromobiaryls **1** as before, but submitted them now to just one equivalent of butyllithium in THF. The aryllithium intermediate was then trapped with one equivalent of ClPCy_2_. The resulting monophosphine **4** was isolated and then submitted to a second bromine–lithium exchange in toluene followed by addition of ClPPh_2_ (Scheme 4). The lithiation–phosphination sequence was not substrate-dependent and reaction conditions were the same for all the different ortho substituents. In this way, pure ligands were obtained on a gram scale.

### Bis(dialkyl)phosphinobiphenyls: Path C

In order to gain access to bis(dicyclohexylphosphino)biphenyls **3**, a double bromine–lithium exchange was performed on the 2,2'-dibromobiaryls **1**. A screening of the reaction conditions revealed higher yields for the double phosphination when the bromine–lithium exchange and trapping with ClPCy_2_ were carried out in toluene rather than in THF and at higher temperature. For example, with R^1^ = Me, OCF_2_O, and OCF_2_CF_2_O, the corresponding diphosphines were obtained in a yield of 54% (in toluene) versus 12% (in THF), 53% versus 36% and 73% versus 35%, respectively (Table 1, entries 1, 2, 9, 10, 11 and 12). A series of bis(dicyclohexylphosphino)biphenyls **6** was obtained in good yield. However, we noticed lower yields for biaryls carrying α-fluorinated ether substituents, such as OCF_2_O (53%; Table 1, entry 9) or OCF_3_ (33%; Table 1, entry 14), in comparison with their nonfluorinated counterparts, OCH_2_O (70%; Table 1, entry 8) and OMe [30] (74%; Table 1, entry 5).

**Table 1:** Synthesis of bis(dicyclohexylphosphino)biphenyls **6**.

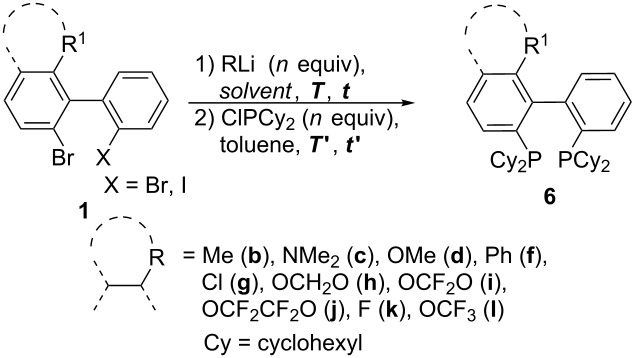											

Entry	R^1^	X	RLi	*n*	Solvent	*T* [° C]	*t*	*T*' [°C]	*t*'	Ligand	Yield

1	Me	Br	BuLi	2	toluene	110	1 h	50	2 h	**6b**	54%
2	Me	Br	BuLi	2	THF	−78	1 h	−78	1 h	**6b**	12%
3	NMe_2_	Br	*t-*BuLi	4	THF	−78	1 h	−78	1 h	**6c**	22%
4	NMe_2_	Br	BuLi	2	toluene	0	15 min	−78	1 h	**6c**	30%
5	OMe [30]	Br	BuLi	2	THF	−78	1 h	−78	1 h	**6d**	74%
6	Ph	Br	*t-*BuLi	4	THF	−78	1 h	−78	1 h	**6f**	66%
7	Cl	Br	BuLi	2	THF	−78	1 h	−78	1 h	**6g**	49%
8	OCH_2_O	Br	BuLi	2	toluene	110	1 h	50	2 h	**6h**	70%
9	OCF_2_O	I	BuLi	3	toluene	25	1 h	25	2 h	**6i**	53%
10	OCF_2_O	I	BuLi	3	THF	−78	1 h	−78	1 h	**6i**	36%
11	O(CF_2_)_2_O	I	BuLi	3	toluene	25	1 h	25	2 h	**6j**	73%
12	O(CF_2_)_2_O	I	BuLi	3	THF	−78	1 h	−78	1 h	**6j**	35%
13	F	Br	BuLi	2	THF	−78	15 min	−78	1 h	**6k**	79%
14	OCF_3_	Br	BuLi	2	toluene	110	1 h	50	2 h	**6l**	33%

**Scheme 5:**
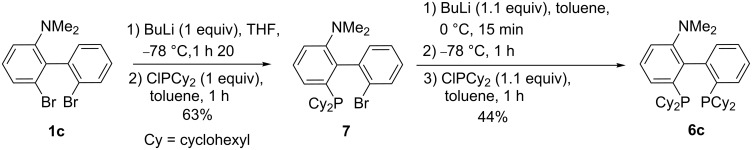
Stepwise bromine–lithium exchange on **1c**.

In the case of 2-*N*,*N*-dimethylamino-2',6-dibromobiphenyl (**1c**), higher yields were obtained when the double bromine–lithium exchange was realized successively instead of simultaneously. Indeed, the double phosphination in THF or toluene gave the corresponding ligand in a very poor yield (Table 1, entries 3 and 4). When the first Br–Li exchange was carried out in THF at −78 °C, followed by trapping with ClPCy_2_, the corresponding monophosphine **7** was obtained in a good yield of 63%. The second Br–Li exchange was performed in toluene at 0 °C, affording ligand **6c** in a moderate yield of 44% after trapping with ClPCy_2_ (Scheme 5).

Single crystal X-ray analysis of **6c** was performed (see Supporting Information File 4) [74].

### Phosphafluorenes and bis(diarylphosphino)biphenyls: Path D and E

When the *ortho,ortho*'-dibromobiphenyls **1** were submitted to a double bromine–lithium exchange in THF followed by trapping with two equivalents of ClPPh_2_, whatever the nature of the ortho substituent (R = Me, OMe, NMe_2_, Cl, OCF_3_, Ph, OCH_2_O, OCF_2_O, OCF_2_CF_2_O, H, F), the formation of an intramolecular cyclization product, a dibenzophosphole, was exclusively observed. This is consistent with the observations of O. Desponds et al. [75]. However, when THF was replaced by toluene, the outcome of the reaction could be modified in favor of the 2,2'-bis(diphenylphosphino)biphenyls **8** [27] which were still contaminated with varying amounts of phosphafluorenes **9** and triphenylphosphine (Table 2).

**Table 2:** Synthesis of bis(diphenylphosphino)biphenyls **8**.

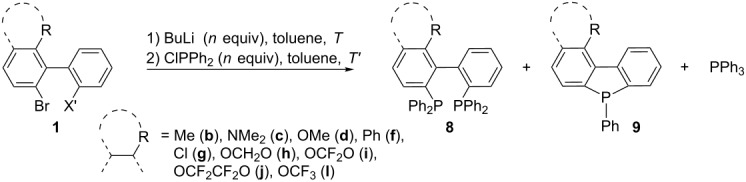						

Cpd.	R	X'	*n* [equiv]	*T*^a^ [°C]	*T*' ^b^ [°C]	Yield **8**^c^

**8b**	Me	Br	2	110	50	30%
**8c**	NMe_2_	Br	2	110	50	44%
**8d**	OMe	Br	2	110	50	17%
**8f**	Ph	Br	2	110	50	24%
**8g**	Cl	Br	2	110	50	23%
**8h**	OCH_2_O	Br	2	110	50	18%
**8i**	OCF_2_O	I	3	25	25	47%
**8j**	OCF_2_CF_2_O	I	3	25	25	34%
**8l**	OCF_3_	Br	2	110	50	10%

^a^Temperature for the halogen/metal exchange; ^b^Trapping temperature; ^c^Isolated yield of **8** after flash-chromatography on silica gel.

## Conclusion

In the present work, we showed how completely regioselective bromine–lithium exchange reactions on polybrominated biphenyls allow the construction of a new family of di- and monophosphine ligands. The required polybrominated biphenyls can be easily obtained through an efficient transition metal-free aryl–aryl coupling protocol developed by our group. The regioselectivity can be explained by recent equilibration studies of M. Schlosser et al. which allowed the determination of ∆∆*G* increments for substituents at the ortho, meta, and para positions.

Overall, the methodology presented in this study offers the possibility to consider new pathways for the synthesis of more sophisticated biaryl scaffolds. Catalytic studies as well as the control of biaryl axial chirality are currently underway and will be reported in due course.

## Supporting Information

File 1 Experimental details and spectroscopic data for new compounds.

File 2 Crystal structure data for **3a**.

File 3 Crystal structure data for **3b**.

File 4 Crystal structure data for **3d**.

File 5 Crystal structure data for **3f**.

File 6 Crystal structure data for **6c**.
