# Supplementary material for: Bromine–lithium exchange: An efficient tool in the modular construction of biaryl ligands
Source: Beilstein J Org Chem. 2011 Sep 14;7:1278–87. doi: 10.3762/bjoc.7.148 (PMC3182437; doi:10.3762/bjoc.7.148)
Supplement: File 1 — Experimental details and spectroscopic data for new compounds. [file Beilstein_J_Org_Chem-07-1278-s001.pdf]

Supporting Information

for

Bromine–lithium exchange: An efficient tool in the modular construction of biaryl ligands

Laurence Bonnafoux, Frédéric R. Leroux* and Françoise Colobert*

Address: Laboratoire de stéréochimie, UMR 7509, CNRS-Université de Strasbourg, ECPM, 25 rue Becquerel, F-67087 Strasbourg Cedex 02, France

Email: Laurence Bonnafoux - laurence.bonnafoux@gmail.com; Frédéric R. Leroux* - frederic.leroux@unistra.fr; Françoise Colobert* - francoise.colobert@unistra.fr

* Corresponding author

Experimental details and spectroscopic data for new compounds.

General remarks: Starting materials, if commercial, were purchased and used as such, provided that adequate checks (melting ranges, refractive indices, and gas chromatography) had confirmed the claimed purity. When known compounds had to be prepared according to literature procedures, pertinent references are given. Air- and moisture-sensitive materials were stored in Schlenk tubes or Schlenk burettes. They were protected by and handled under an atmosphere of argon, with appropriate glassware. Et_2O and THF were dried by distillation from sodium after the characteristic blue color of sodium diphenyl ketyl (benzophenone-sodium "radical-anion") had been found to persist. Ethereal or other organic extracts were dried by washing with brine and then by storage over sodium sulfate. If no reduced pressure is specified, boiling ranges (bp) refer to ordinary atmosphere conditions (725 $\pm$ 25 Torr). Melting ranges (mp) given were found to be reproducible after recrystallization, unless stated otherwise ("decomp."), and are uncorrected. Thin-layer chromatography (TLC) was carried out on 0.25 mm Merck silica-gel (60-F254). Column chromatography was carried out on a column packed with silica-gel 60N spherical neutral size 63–210 µm. Gas chromatography monitoring was performed with HP 6890 series apparatus, capillary column HP-5 (5% phenylmethylsiloxane), FID detector (250 °C), with the following program: 60 °C for 3 min, 30 °C/min until 300 °C, 30 °C for 45 min, injector (230 °C). BuLi and *t*-BuLi were used as solutions in hexanes or pentane and their concentrations were determined following the Wittig–Harborth Double Titration method ((total base) – (residual base after reaction with 1,2-dibromoethane)). Organometallic reagents were usually checked by Gilman tests 1 (all organolithiums) and 2 (only for alkyllithiums) [1]. The starting materials obtained by aryne coupling will be reported elsewhere (**1g**, **1h**, **1i**, **1j**, **1k** and **1l**) [2]. Compounds **4c**, **5c** and **6d** have been published previously and prepared in a different manner. The spectroscopic data were consistent with the previously obtained ones [3]. The spectroscopic data of bis(diphenylphosphino)biphenyls have been discussed in detail previously [4].

Regioselective bromine–lithium between 2,2',6-tribromobiphenyl ***(1a)*** [5] and butyllithium

2,2'-Dibromo-6-methylbiphenyl (1b): At –78 °C, BuLi (25.0 mmol, 1 equiv) in hexanes (16.0 mL) was added to a solution of 2,2',6,-tribromo-1,1'-biphenyl (**3**; 25.0

mmol, 1 equiv) in THF (60.0 mL). The mixture was treated with MeI (25.0 mmol, 3.55 g, 1 equiv) and allowed to reach 25 °C. Then, it was treated with a saturated aqueous solution of ammonium chloride (60.0 mL). The mixture was extracted with ethyl acetate (3 × 50.0 mL), and the combined organic layers were dried over sodium sulfate. After concentration of the solvent, the residue was purified by chromatography on silica gel with a mixture of hexane and ethyl acetate as eluent. 2,2'-Dibromo-6-methylbiphenyl (**2c**; 96%) was obtained as a colorless solid. m.p. 54–56 °C. ^{1}H NMR ($CDCl_3$, 300 MHz): δ = 7.80 (dd, 1H, *J* = 8.0 Hz, 1.1 Hz), 7.63 (d, 1H, *J* = 7.8 Hz), 7.53 (td, 1H, *J* = 7.5, 1.2 Hz), 7.22–7.42 (m, 4H), 2.19 (s, 3H) ppm. ^{13}C NMR ($CDCl_3$, 75 MHz): δ = 132.7, 130.7, 123.0, 129.3, 129.2, 128.8, 127.6, 21.0 ppm. $C_{13}H_{10}Br_2$ (326.03): calcd. (%) C 47.89, H 3.09; found (%): C 48.01, H 3.14.

(2',6-Dibromo-1,1'-biphenyl-2-yl)dimethylamine (1c): At –78 °C, BuLi (16.3 mmol, 1 equiv) in hexanes (10.6 mL) was added dropwise to a solution of 2,2',6-tribromobiphenyl (**1a**; 6.36 g, 16.3 mmol, 1 equiv) in THF (33:0 mL). Benzenesulfonyl azide [6] (2.98 g, 16.3 mmol, 1 equiv) was added dropwise and after 1 h the reaction mixture was allowed to reach 25 °C. After addition of distillated water (40.0 mL), the aqueous layer was extracted with ethyl acetate (3 × 40.0 mL). The combined organic layers were dried, filtered and evaporated. *6-Azido-2,2'-dibromobiphenyl* was obtained as a viscous brown oil which was used for the reduction of the azide without further purification.

^{1}H NMR (300 MHz, $CDCl_3$): δ = 7.72 (d, 1H, *J* = 1.3 Hz), 7.64 (d, 1H, *J* = 2.6 Hz), 7.40 (td, 2H, *J* = 7.5, 1.2 Hz), 7.29 (d, 2H, *J* = 1.5 Hz), 7.26 (d, 1H, *J* = 0.9 Hz) ppm. ^{13}C NMR ($CDCl_3$, 75 MHz): δ = 117.4, 124.4, 124.8, 125.1, 128.8, 130.7, 130.8, 131.7, 131.8, 139.0, 139.9, 142.0 ppm. *v* (film) : 2102,91 cm^{-1} (N_3).

A solution of *6-azido-2,2'-dibromobiphenyl* (5.75 g, 16.3 mmol, 1 equiv) in Et_2O (150 mL) was added slowly by cannula to a stirred solution of $LiAlH_4$ (0.64 g, 16.8 mmol, 1.05 equiv) in Et_2O (150 mL). After the addition was completed, the reaction mixture was heated at 50 °C for 4.5 h. After cooling to rt, the reaction mixture was diluted with Et_2O (100 mL) and carefully hydrolyzed with water (200 mL). The solution was filtered through a filtering funnel. The aqueous phase was extracted with Et_2O (2 x 80.0 mL) and the combined organic layers were dried and evaporated. *6,2'-Dibromobiphenyl-2-ylamine* was obtained as an orange, crystalline solid and directly submitted to a reductive aminomethylation. m.p. 63–66 °C. ^{1}H NMR (300 MHz,

$CDCl_3$): δ = 7.74 (dd, 1H, *J* = 7.8, 0.9 Hz), 7.46 (td, 1H, *J* = 7.5, 1.2 Hz), 7.33 (dd, 1H, *J* = 7.5, 1.8 Hz), 7.28 (dd, 1H, *J* = 6.9, 1.8 Hz), 7.00 (m, 2H), 6.70 (dd, 1H, *J* = 6.9, 2.1), 3.5 (s, 2H) ppm. ^{13}C NMR (75 MHz, $CDCl_3$): δ = 114.1, 122.1, 124.5, 124.6, 127.3, 128.2, 129.9, 130.0, 131.8, 133.2, 138.8, 145.0 ppm.

Sodium cyanoborohydride (2.04 g, 48.0 mmol, 3 equiv) was added in small portions to a stirred solution of *2',6-dibromobiphenyl-2-ylamine* (5.68 g, 16.0 mmol, 1 equiv) and 37% aqueous formaldehyde (13.0 mL, 160 mmol, 10 equiv) in MeCN (64.0 mL) placed in a cool bath. Glacial acetic acid (1.76 mL, 18.0 mmol, 1.7 equiv) was added very slowly and the reaction mixture was stirred at rt for 2 h. An additional glacial acetic acid portion (1.76 mL, 18.0 mmol, 1.7 equiv) was added and stirring was continued for additional 2 h. The reaction mixture was poured into Et_2O (200 mL) and then washed with 1 N aqueous KOH solution (3 × 60.0 mL) and brine (1 × 60.0 mL). The organic phase was dried and evaporated in vacuo. The crude product was submitted to chromatography on silica gel with a mixture of ethyl acetate/cyclohexane = 9:1. It afforded (2',6-dibromobiphenyl-2-yl)-dimethylamine (**1c**; 4.56 g, 12.8 mmol, overall yield: 79%) as yellow crystals. m.p. 67–69 °C. ^{1}H NMR (300 MHz, $CDCl_3$): δ = 7.65–7.72 (m, 1H), 7.30–7.44 (m, 2H), 7.15–7.29 (m, 3H), 7.06 (d, 1H, *J* = 8.1 Hz), 2.54 (s, 6H) ppm. ^{13}C NMR (75 MHz, $CDCl_3$): δ = 43.6, 117.9, 125.2, 125.5, 125.9, 127.2, 128.8, 129.6, 132.2, 132.7, 135.4, 141.4, 153.8 ppm. $C_{14}H_{13}Br_2N$ (355.07): calcd. (%) C 47.36, H 3.69, N 3.95; found C 48.00, H 3.84, N 3.62.

2,2'-Dibromo-6-methoxybiphenyl (1d): At –78 °C, BuLi (45.5 mmol, 1 equiv) in hexanes (30.0 mL) was added to a solution of 2,2',6-tribromobiphenyl (**1a**; 17.8 g, 45.5 mmol, 1 equiv) in THF (95.0 mL). Immediately after the addition was completed, fluorodimethoxyborane Et_2O (10.0 mL, 52.0 mmol, 1.2 equiv) was added and the reaction mixture was then allowed to reach 0 °C. After 30 min, a 3 M aqueous solution of NaOH (18.0 mL) and 30% hydrogen peroxide (5.00 mL) were added dropwise. The reaction mixture was then allowed to reach 25 °C overnight. After addition of water (100 mL), the organic phase was separated and the aqueous layer was extracted with dichloromethane (3 × 50.0 mL). The combined organic layers were dried over sodium sulfate before being evaporated. Purification by column chromatography afforded *2',6-dibromobiphenyl-2-ol* (10.6 g, 32.3 mmol, 71%) as a slightly yellow oil.

^{1}H NMR ($CDCl_3$, 400 MHz): δ = 7.77 (dd, 1H, *J* = 8.3, 1.3 Hz), 7.47 (dt, 1H, *J* = 7.7, 1.4 Hz), 7.36 (dt, 1H, *J* = 8.0, 1.7 Hz), 7.29 (dd, 1H, *J* = 7.5, 1.7 Hz), 7.28 (dd, 1H, *J* = 7.7, 1.3 Hz), 7.19 (t, 1H, *J* = 8.3 Hz), 6.97 (dd, 1H, *J* = 8.3, 1.1 Hz,), 4.59 (s, 1H) ppm. ^{13}C NMR ($CDCl_3$, 101 MHz): δ = 156.4, 142.0, 136.1, 131.2, 130.8, 129.9, 129.5, 127.0, 122.7, 117.5, 116.8 ppm. $C_{12}H_8Br_2O$ (328.00): calcd. (%) C 43.94, H 2.46; found C 44.19, H 2.57.

2',6-Dibromobiphenyl-2-ol was then dissolved in acetone (100 mL). Potassium carbonate (6.00 g, 45.0 mmol, 1 equiv) and MeI (7.76 g, 3.40 mL, 1.2 equiv) were successively added. The mixture was heated at 60 °C overnight. After cooling to 25 °C, water (50.0 mL) was added and the organic layer was extracted with dichloromethane (4 × 100 mL). The combined organic layers were dried, filtered and evaporated. The crude product was purified by chromatography which afforded pure 2',6-dibromo-2-methoxy-1,1'-biphenyl (**1d**; 11.6 g, 34.0 mmol, 95%) as light yellow crystalline solid. Overall yield: 68%. m.p. 93–95 °C. ^{1}H NMR ($CDCl_3$, 300 MHz): δ = 7.60 (dd, 1H, *J* = 7.9, 1.0 Hz), 7.38 (td, 1H, *J* = 7.4, 1.2 Hz), 7.2 (m, 4H), 6.86 (dd, 1H, *J* = 7.9, 1.3 Hz), 3.66 (s, 3H) ppm. ^{13}C NMR ($CDCl_3$, 75 MHz): *δ* = 158.0, 138.9, 132.5, 131.5, 131.3, 130.0, 129.2, 127.2, 124.7, 124.6, 124.4, 109.9, 56.2 ppm. $C_{13}H_{10}Br_2O$ (342.03): calcd. (%) C 45.32, H 2.95; found C 45.32, H 2.85.

2,2'-Dibromo-6-iodobiphenyl (**1e**)**:** At –78 °C, BuLi (47.2 mmol, 1 equiv) in hexanes (30.5 mL) was added to a solution of 2,2',6,-tribromo-1,1'-biphenyl (**1a**; 47.2 mmol, 18.4 g, 1 equiv) in THF (94.0 mL). The mixture was consecutively treated with iodine (47.2 mL, 12.0 g, 1 equiv) in THF (50.0 mL) and allowed to reach 25 °C. Then, it was treated with a saturated aqueous solution of ammonium chloride (50.0 mL). The mixture was extracted with ethyl acetate (3 × 100 mL), and the combined organic layers were dried over sodium sulfate. After concentration of the solvent, the residue was purified by chromatography on silica gel with a mixture of hexane and ethyl acetate as eluent. 2,2'-Dibromo-6-iodobiphenyl *(***1e***)* (83%) was obtained as a yellow solid. m.p. 100–103 °C. ^{1}H NMR ($CDCl_3$, 300 MHz): *δ* = 7.83 (td, 1H, *J* = 8.2, 1.1 Hz), 7.61 (td, 2H, *J* = 7.5, 1.2 Hz), 7.19–7.35 (m, 2H), 7.08 (dd, 1H, *J* = 7.51, 1.66 Hz), 6.86 (t, 1H, *J* = 8.0 Hz) ppm. ^{13}C NMR ($CDCl_3$, 75 MHz): *δ* = 145.7, 145.2, 138.1, 132.8, 132.6, 130.7, 130.6, 129.9, 127.6, 123.4, 123.1, 100.1 ppm. $C_{12}H_7Br_2I$ (437.90): calcd. (%) C 32.91, H1.61; found C 33.03, H 1.72.

2,6'-Dibromo-1,1':2',1''-terphenyl (1f): To a solution of 2,2'-dibromo-6-iodobiphenyl (**1e**: 3.88 g, 8.86 mmol, 1 equiv), sodium carbonate (5.63 g, 53.2 mmol, 6 equiv), phenylboronic acid (2.16 g, 17.7 mmol, 2 equiv), in MeCN (130 mL) and water (130 mL), $Pd(PPh_3)_4$ (0.51 g, 0.44 mmol, 5 mol%) was added. The mixture was heated at 90 °C for 3 h and was then allowed to reach 25 °C. Water (100 mL) was added, followed by extraction with ethyl acetate (4 × 100 mL). The combined organic layers were dried over sodium sulfate, filtered and evaporated. The dark brown oil was purified by filtration through silica gel with cyclohexane as eluent to afford a colorless oil (**1f**, 3.33 g, 8.58 mmol, 95%). ^1H NMR ($CDCl_3$, 300 MHz): δ =7.61 (dd, 1H, J = 7.5, 1.7 Hz), 7.44 (dd, 1H, J = 7.7, 1.1 Hz), 7.30–7.20 (m, 2H), 7.10–7.00 (m, 6H), 7.0–6.9 (m, 2H) ppm. ^{13}C NMR ($CDCl_3$, 75 MHz): δ = 143.6, 141.2, 140.5, 140.3, 132.3, 131.9, 131.6, 129.6, 129.3 (2C), 129.1, 129.0, 127.6, 127.4, 127.0, 126.9, 124.8, 124.7 ppm. MS (EI): *m/z* (%) = 388 (7) $[M]^+$, 308 $[M – Br]^+$, 228 $[M – 2 Br]^+$. $C_{18}H_{12}Br_2$ (388.10): calcd. (%) C 55.71, H 3.12; found C 55.13, H 3.47.

*General Protocol: Regioselective bromine–lithium exchange between 2,2'-dibromobiphenyls **1*** and butyllithium

At –78 °C, butyllithium (1 equiv) in hexanes was added dropwise to a solution of dibromobiaryl (1 equiv) in THF (150 mL for 33.0 mmol). After the addition was completed, iodomethane (1 equiv) was added dropwise and the reaction mixture was then allowed to reach 25 °C. Water (80.0 mL) was added, followed by extraction with ethyl acetate (3 × 80.0 mL). The combined organic layers were dried over sodium sulfate, filtered, and evaporated.

2'-Bromo-*N*,*N*,6-trimethylbiphenyl-2-amine (2a)

Starting from (6,2'-dibromobiphenyl-2-yl)-dimethyl-amine (**1c**). Flash chromatography on silica gel with a mixture of ethyl acetate/cyclohexane 2:98 afforded (2'-bromo-6-methyl-biphenyl-2-yl)-dimethylamine (**2a**; 0.67 g, 2.31 mmol, 81%) as colorless cubes. ^1H NMR (300 MHz, $CDCl_3$): δ = 7.67 (d, J = 7.7 Hz, 1H), 7.14–7.42 (m, 4H), 6.94–7.05 (m, 2H), 2.51 (s, 6H), 2.00 (s, 3H). ^{13}C NMR (75 MHz, $CDCl_3$): δ = 152.2, 141.3, 137.5, 135.4, 132.7, 132.0, 128.3, 128.1, 127.2, 123.9, 116.5, 44.0 (2C), 20.4. $C_{15}H_{16}BrN$ (290.20): calcd. C (%) 62.08, H 5.56, N 4.83; found C 62.13, H 5.64, N 4.91.

2'-Bromo-6-methoxy-2-methylbiphenyl (2b): Starting from 2,2'-dibromo-6-methoxybiphenyl (**1d**). Purification by filtration through silica gel followed by crystallization from methanol afforded 2'-bromo-6-methoxy-2-methylbiphenyl (**2b**, 74%) as colorless cubes. mp 58–59 °C. ^{1}H NMR ($CDCl_3$, 300 MHz): δ = 7.61 (dd, *J* = 7.7, 0.9 Hz, 1H), 7.31 (td, *J* = 7.4, 1.2 Hz, 1H), 7.22 (t, *J* = 7.9 Hz, 1H), 7.2–7.1 (m, 2H), 6.85 (d, *J* = 7.5 Hz, 1H), 6.77 (d, *J* = 8.2 Hz, 1H), 3.66 (s, 3H), 1.95 (s, 3H). ^{13}C NMR ($CDCl_3$, 75 MHz): δ= 156.8, 139.0, 137.7, 132.5, 131.4, 130.1, 128.7, 128.6, 127.3, 124.6, 122.2, 108.4, 55.9, 19.9. $C_{14}H_{13}BrO$ (276.01): calcd. (%) C 60.67, H 4.73; found (%) C 61.18, H 4.61.

2''-Bromo-3'-methyl-[1,1';2',1'']terphenyl (2c): Starting from 2,2'-dibromo-6-phenylbiphenyl (**1f**). The brown oil was purified by filtration on silica gel which afforded 2''-bromo-3'-methyl-[1,1';2',1'']terphenyl (**2c**, 93%) as white crystals. ^{1}H NMR ($CDCl_3$, 300 MHz): δ = 7.46 (dd, *J* = 7.9, 0.9, 1H), 7.31 (d, *J* = 7.5, 1H), 7.3–7.2 (m, 2H), 7.1–7.0 (m, 6H), 7.0–6.9 (m, 2H), 2.06 (s, 3H). ^{13}C NMR ($CDCl_3$, 75 MHz): δ = 141.6, 141.5, 141.4, 139.4, 136.7, 132.3, 131.9, 129.3 (2C), 128.9, 128.4, 127.8, 127.5, 127.4 (2C), 127.0, 126.4, 124.9. $C_{19}H_{15}Br$ (323.22): calcd. (%) C 70.60, H 4.68; found C 70.48, H 4.90.

4-(2-bromophenyl)-5-methyl-benzo[1,3]dioxole (2d): Starting from 5-bromo-4-(2-bromophenyl)-benzo[1,3]dioxole (**1h**). Purification by flash chromatography afforded 4-(2-bromophenyl)-5-methyl-benzo[1,3]dioxole (**2d**, 95%) as a colorless oil. ^{1}H NMR ($CDCl_3$, 300 MHz): δ = 7.61 (dd, *J* = 7.5, 1.0 Hz, 1H), 7.31 (td, *J* = 7.0, 8.1 Hz, 1H), 7.3–7.1 (m, 2H), 6.7–6.6 (m, 2H), 5.9–5.7 (m, 2H), 1.95 (s, 3H). ^{13}C NMR ($CDCl_3$, 100 MHz): 145.6, 137.0, 133.3, 131.7, 130.1, 130.3, 129.8, 127.8, 124.6, 123.3, 122.5, 108.2, 101.5, 19.5. HRMS for $C_{14}H_{11}BrO_2$ [M+Na]: calcd. 312.9835; found 312.9846.

<u>General Procedures</u>: Synthesis of monophosphines

Conditions a: At –78 °C, butyllithium (1 equiv) in hexanes was added dropwise to a solution of the substrate (1 equiv) in THF (4.00 mL for 2 mmol). After 1 h, a solution of chlorophosphine (1 equiv) in toluene (2.00 mL) was added dropwise. After 1 h, the

mixture was allowed to reach 25 °C and filtered through silica gel with diethyl ether. The organic layer was evaporated and the crude product was purified by chromatography on silica gel. **Conditions b:** At 0 °C, butyllithium (1.1 equiv) in hexanes was added dropwise to a solution of the substrate (1 equiv) in toluene (14.0 mL for 7 mmol). After 15 min the reaction mixture was cooled to –78 °C. A solution of chlorophosphine (1.1 equiv) in toluene (7.00 mL for 7 mmol) was added dropwise. After 1 h, the mixture was allowed to reach 25 °C and filtered through silica gel with diethyl ether. The organic layer was evaporated.

2'-(Dicyclohexylphosphino)-*N,N*-6-trimethylbiphenyl-2-amine (3a): At –78 °C, butyllithium (3.25 mmol) in hexanes (2.00 mL) was added to a solution of (2'-bromo-6-methyl-biphenyl-2-yl)dimethylamine (**2a**; 0.79 mg, 2.71 mmol) in THF (5.00 mL). After 1 h, a solution of chlorodicyclohexylphosphine (0.90 mL, 0.95 mg, 4.07 mmol) in THF (1.00 mL) was added and the mixture allowed to reach 25 °C. After 1 h, a saturated aqueous solution of ammonium chloride (10.0 mL) was added followed by extraction with ethyl acetate (3 × 30 mL). The combined organic phases were dried and evaporated. Flash chromatography on silica gel with a mixture of ethyl acetate/cyclohexane 2:98 and crystallization of the residue from hexanes afforded (2'-dicyclohexylphosphanyl-6-methylbiphenyl-2-yl)dimethylamine (**3a**; 0.73 mg, 1.79 mmol, 66%) as colorless prisms. ^{1}H NMR (300 MHz, $CDCl_3$): δ = 7.60 (d, *J* = 7.2 Hz, 1H), 7.17–7.43 (m, 4H), 6.86–6.96 (m, 2H), 2.43 (s, 6H), 1.99 (s, 3H), 1.49–1.95 (m, 11H), 0.83–1.37 (m, 11H). ^{13}C NMR (75 MHz, $CDCl_3$): δ = 152.1 (d, *J* = 2.2 Hz), 147.5 (d, *J* = 31.3 Hz), 137.0, 136.2 (d, *J* = 19.2 Hz), 135.9 (d, *J* = 6.0 Hz), 133.0 (d, *J* = 3.3 Hz), 132.0 (d, *J* = 6.6 Hz), 127.9, 127.8, 125.8, 123.8, 115.4, 44.0 (2C), 35.5 (d, *J* = 14.8 Hz), 34.3 (d, *J* = 15.36 Hz), 30.6, 30.4, 30.2, 29.9, 29.1 (d, *J* = 8.2 Hz), 27.8 (d, *J* = 10.4 Hz), 27.5 (d, *J* = 11.0 Hz), 27.4 (d, *J* = 6.6 Hz), 26.6 (d, *J* = 17.0 Hz), 21.38. ^{31}P NMR (121 MHz, $CDCl_3$): δ = –8.29. $C_{27}H_{38}NP$ (407.58): calcd. (%) C 79.57, H 9.40, N 3.44; found C 79.47, H 9.44, N 3.29.

(2-Methoxy-6-methylbiphenyl-2’-yl)diphenylphosphine (3b): Prepared under *conditions b* and starting from 2'-bromo-6-methoxy-2-methylbiphenyl (**2b**). Triturating the crude product with methanol afforded (2-methoxy-6-methylbiphenyl-2’-yl)diphenylphosphane (**3b**, 68%) as a white powder. mp 105–106 °C. ^{1}H NMR ($CDCl_3$, 300 MHz): δ = 7.35 (td, *J* = 7.4, 1.1 Hz, 1H), 7.2–7.0 (m, 14H), 6.78 (d, *J* =

7.6 Hz, 1H), 6.54 (d, *J* = 8.2 Hz, 1H), 3.17 (s, 3H), 1.87 (s, 3H). ^{13}C NMR ($CDCl_3$, 75 MHz): δ = 157.1, 144.3 (d, *J* = 33.5 Hz), 138.2, 138.1, 137.6, 137.5, 137.4 (d, *J* = 2.8 Hz), 137.3, 134.1 (d, *J* = 2.2 Hz), 133.9, 133.8, 133.7, 133.5, 130.3, 130.1 (d, *J* = 6.0 Hz), 129.1, 128.4, 128.3, 128.2, 128.0, 127.9, 127.2, 122.0, 107.5, 54.8, 20.4. ^{31}P NMR ($CDCl_3$, 161 MHz): δ= –12.3. $C_{26}H_{23}OP$ (382.15): calcd. (%) C 81.66, H 6.06; found C 81.82, H 6.15.

Dicyclohexyl(2-methoxy-6-methylbiphenyl-2'-yl)phosphane (3c): Prepared under *conditions b* and starting from 2'-bromo-6-methoxy-2-methylbiphenyl (**2f**). Triturating the crude product with methanol afforded dicyclohexyl(2-methoxy-6-methylbiphenyl-2'-yl)phosphane (**3b**, 76%) as a white powder. mp 103–105 °C. ^{1}H NMR ($CDCl_3$, 300 MHz): δ = 7.49 (d, *J* = 7.1 Hz, 1H), 7.3–7.2 (m, 2H), 7.2–7.1 (m, 1H), 7.1–7.0 (m, 1H), 6.79 (d, *J* = 7.5 Hz, 1H), 6.67 (d, *J* = 8.2 Hz, 1H), 3.58 (s, 3H), 1.91 (s, 3H), 1.7–1.4 (m, 11H), 1.2–0.9 (m, 11H). ^{13}C NMR ($CDCl_3$, 75 MHz): δ = 156.8, 145.6 (d, *J* = 33.8 Hz), 137.4, 135.8 (d, J = 1.6 Hz), 132.5 (d, *J* = 18.7 Hz), 131.1 (d, *J* = 6.6 Hz), 130.6 (d, *J* = 6.0 Hz), 128.4, 128.1, 126.2, 121.9, 107.2, 54.9, 34.8 (d, *J* = 14.8 Hz), 33.3 (d, *J* = 13.7 Hz), 30.3, 30.1, 29.9, 29.8, 29.6, 29.6, 29.4, 27.8, 27.7, 27.4, 27.3, 27.2, 26.5, 20.8 (d, *J* = 4.4 Hz). ^{31}P NMR ($CDCl_3$, 161 MHz): δ = –8.1. $C_{26}H_{35}OP$ (394.53): calcd. C 79.15%, H 8.94%; found C 79.12%, H 8.96%.

(3'-Methyl-[1,1';2',1'']terphenyl-2''-yl)diphenylphosphane (3d): Prepared under *conditions a* and starting from 2''-bromo-3'-methyl-[1,1';2',1'']terphenyl (**2c**). The crude product was purified by chromatography on silica gel. Triturating from methanol afforded (3'-methyl-[1,1';2',1'']terphenyl-2''-yl)diphenylphosphane (**3d**, 65%) as a colorless powder. mp 120–122 °C. ^{1}H NMR ($CDCl_3$, 300 MHz): δ = 7.3–7.2 (m, 3H), 7.2–7.1 (m, 4H), 7.1–7.0 (m, 12H), 7.0–6.9 (m, 1H), 6.6–6.4 (m, 2H), 1.61 (s, 3H). ^{13}C NMR ($CDCl_3$, 75 MHz): δ = 146.58 (d, *J* = 33.5 Hz, 3C), 142.1 (d, *J* = 1.7 Hz), 141.8 (d, *J* = 2.2 Hz), 139.7 (d, *J* = 6.6 Hz), 138.3 (d, *J* = 13.7 Hz),137.0 (d, *J* = 5.5 Hz), 134.6 (2C), 134.3, 132.6 (d, *J* = 18.1 Hz), 131.5 (d, *J* = 6.6 Hz), 130.5 (2C), 128.9, 128.7, 128.4–128.0 (6C), 127.7, 127.6, 127.5, 127.3 (3C), 126.0, 20.8. ^{31}P NMR ($CDCl_3$, 161 MHz): δ= -13.7. HRMS for $C_{31}H_{25}P$ [M+H]: calcd. 429.1767; found 429.1742.

Dicyclohexyl(3'-methyl-[1,1';2',1'']terphenyl-2''-yl)phosphane (3e): Prepared under *conditions a* and starting from 2''-bromo-3'-methyl-[1,1';2',1'']terphenyl (**2c**). Triturating from methanol afforded dicyclohexyl(3'-methyl-[1,1';2',1'']terphenyl-2''-yl)phosphane (**3e**, 66%). mp 106–108 °C. ^{1}H NMR ($CDCl_3$, 300 MHz): δ = 7.36–7.06 (m, 12H), 2.04 (s, 3H), 1.7–1.4 (m, 11H),1.3–0.9 (m, 11H). ^{13}C NMR ($CDCl_3$, 75 MHz): δ = 147.53 (d, *J* = 31 Hz), 142.4, 141.2, 140.2, 136.7, 135.47 (d, *J* = 20 Hz), 133.1, 132.6, 132.5, 130.7 (3C), 129.0, 128.0, 127.6 (3C), 126.2 (d, *J* = 31 Hz), 35.3 (d, *J* = 16 Hz), 33.4 (d, *J* = 15 Hz), 30.2, 30.1, 29.6, 29.5, 29.4, 29.3, 27.8–27 (m), 26.5, 26.4, 21.1. ^{31}P NMR ($CDCl_3$, 161 MHz): δ = –9.2. HRMS for $C_{31}H_{38}P$: calcd. 441.2706; found 441.2703.

Dicyclohexyl[2-(5-methyl-benzo[1,3]dioxol-4-yl)-phenyl]phosphane (3f)
Prepared under *conditions a* and starting from 4-(2-bromophenyl)-5-methyl-benzo[1,3]dioxole (**2d**).The residue was purified by flash chromatography and triturating from methanol afforded dicyclohexyl[2-(5-methyl-benzo[1,3]dioxol-4-yl)-phenyl]phosphane (**3f**, 63%) as a colorless powder. mp 128–130 °C. ^{1}H NMR ($CDCl_3$, 300 MHz): δ = 7.6–7.4 (m, 1H), 7.4–7.2 (m, 2H), 7.2–7.0 (m, 1H), 6.7–6.5 (m, 2H), 5.82 (d, *J* = 1.5 Hz, 1H), 5.71 (s, 1H), 1.93 (s, 3H), 1.7–1.4 (m, 12H), 1.3–0.8 (m, 10H). ^{13}C NMR ($CDCl_3$, 100 MHz): 144.3, 144.3, 143.7, 142.4, 142.0, 131.9 (d, *J* = 3.3 Hz), 129.3 (d, *J* = 6 Hz), 128.8, 127.7, 126.1, 120.9, 106.3, 99.5, 33.6 (d, *J* = 12.1 Hz), 32.0 (d, *J* = 12.6 Hz), 29.0 (d, *J* = 8.8 Hz), 29.1, 29.0, 28.9, 28.8, 28.7, 28.5, 28.1, 28.0, 26.7, 26.6, 26.5, 26.5, 26.3, 26.2, 26.1, 25.4, 18.9 (d, *J* = 5.5 Hz). ^{31}P NMR ($CDCl_3$, 161 MHz): δ= –8.0. HRMS for $C_{26}H_{33}O_2P$ [M+H]: calcd. 409.2291; found 409.2272.

<u>General Procedure</u>: *Synthesis of intermediate monophosphines towards mixed diphosphines*
At –78 °C, butyllithium (1 equiv) in hexanes was added dropwise to a solution of the dibromobiaryl (1 equiv) in THF (20.0 mL for 10.0 mmol). After 1 h at –78 °C, a solution of chlorodicyclohexylphosphine (1 equiv) in toluene (10.0 mL) was added dropwise. Stirring was continued for 1 h. The reaction mixture was then allowed to reach 25 °C and filtered through silica gel with diethyl ether. The organic layer was evaporated.

(2'-Bromo-6-chlorobiphenyl-2-yl)dicyclohexylphosphine (4a): Synthesized according to the general procedure and starting from 2,2'-dibromo-6-chlorobiphenyl (**1g**). Chromatography on silica gel followed by crystallization from methanol afforded (2'-bromo-6-chlorobiphenyl-2-yl)dicyclohexylphosphine (**4a**; 73%) as a colorless solid. ^{1}H NMR ($CDCl_3$, 300 MHz): δ = 7.58 (dd, *J* = 7.9, 1.2 Hz, 1H), 7.44 (d, *J* = 7.6 Hz, 1H), 7.40 (dd, *J* = 7.9, 1.0 Hz, 1H), 7.32–7.23 (m, 2H), 7.19 (t, *J* = 7.7 Hz, 1H), 7.07 (dd, *J* = 7.5, 1.8 Hz, 1H), 1.95–1.78 (m, 1H), 1.76–1.49 (m, 10H), 1.49–1.38 (m, 1H), 1.28–0.85 (m, 10H). MS (EI): m/z (%)= 463.1 (<1) [M^+], 383.1 (100) [M^+–Br], 349.2 (1) [M^+–Br–Cl], 183.1 (26) [M^+–Br–Cl–2 PCy_2]. $C_{24}H_{29}BrClP$ (463.82): calcd. (%) C 62.15, H 6.30, found C 62.54, H 5.99.

(2'-Bromo-6-dicyclohexylphosphanylbiphenyl-2-yl)dimethylamine (4b): Synthesized according to the general procedure and starting from (2',6-dibromobiphenyl-2-yl)dimethylamine (**1c**). The crude product was purified by chromatography on silica gel with a mixture of ethyl acetate/cyclohexane (1:19) as eluent. Triturating the slight yellow oil from methanol afforded pure (2'-bromo-6-dicyclohexylphosphanylbiphenyl-2-yl)dimethylamine (**4b**, 63%) as a white powder. mp 115–116 °C. ^{1}H NMR ($CDCl_3$, 300 MHz): δ = 7.54 (d, *J* = 8.3 Hz, 1H), 7.3–7.2 (m, 3H), 7.1–7.0 (m, 3H), 2.41 (s, 6H), 1.6–1.5 (m, 11H), 1.2–0.9 (m, 11H). ^{13}C NMR ($CDCl_3$, 75 MHz): δ = 152.7 (d, *J* = 7.7 Hz), 143.7 (d, *J* = 29.6 Hz), 141.3 (d, *J* = 7.1 Hz), 136.8 (d, *J* = 19.8 Hz), 134.2, 134.1, 132.2, 128.0 (d, *J* = 36.2 Hz), 127.0, 126.9, 125.9, 119.9, 44.2 (2C), 35.7 (d, *J* = 15.4 Hz), 34.1 (d, *J* = 14.3 Hz), 30.9, 30.7, 30.3, 30.2, 29.9, 29.5, 29.4, 27.6, 27.6, 27.5, 27.5, 27.4, 27.4, 27.3, 27.2, 26.5, 26.5, 26.4. ^{31}P NMR ($CDCl_3$, 161 MHz): δ = –8.9. $C_{26}H_{35}BrNP$ (472.44): calcd. (%) C 66.10, H 7.47, N 2.96, found C 65.85, H 7.74, N 2.70.

(2''-Bromo-[1,1':2',1'']terphenyl-3'-yl)dicyclohexylphosphane (4d): Synthesized according to the general procedure and starting from 2,2'-dibromo-6-phenylbiphenyl (**1f**). Triturating the crude product from methanol afforded 2''-bromo-[1,1';2',1'']terphenyl-3'-yl)dicyclohexylphosphane (**4d**, 75%) as a white powder. mp 116–118 °C. ^{1}H NMR ($CDCl_3$, 300 MHz): δ = 7.6–7.5 (m, 1H), 7.4–7.2 (m, 3H), 7.1–7.0 (m, 6H), 6.95 (t, *J* = 7.0 Hz, 2H), 1.8–1.4 (m, 12H), 1.3–0.9 (m, 11H). ^{13}C NMR

($CDCl_3$, 75 MHz): δ = 146.9 (d, J = 31 Hz), 142.1 (d, J = 6 Hz), 141.6, 134.0, 134.0, 131.9 (2C), 124.9, 129.4 (2C), 128.4, 127.3 (2C), 127.0, 126.4, 125.6, 125.4, 125.3, 35.7 (d, J = 15 Hz), 33.8 (d, J = 13 Hz), 31.0, 30.8, 30.4, 30.4, 30.2, 30.1, 29.9, 29.3, 29.2, 27.6, 27.6, 27.5, 27.5, 27.4, 27.3, 27.3, 27.2, 26.5, 26.4. ^{31}P NMR ($CDCl_3$, 161 MHz): δ = –9.0. HRMS for $C_{30}H_{34}BrP$ [M+H]: calcd. 505.1654; found 505.1648.

(4-(2-Bromophenyl)benzo[*d*][1,3]dioxol-5-yl)dicyclohexylphosphine (4e): Synthesized according to the general procedure and starting from 5-bromo-4-(2-bromophenyl)benzo[1,3]dioxole (**1h)**. Crystallization from methanol afforded (4-(2-bromophenyl)benzo[*d*][1,3]dioxol-5-yl)dicyclohexylphosphine (**4e**; 55%) as colorless crystals. 168–169 °C. ^{1}H NMR ($CDCl_3$, 300 MHz): δ = 7.6–7.5 (m, 1H), 7.4–7.3 (m, 1H), 7.3–7.2 (m, 3H), 6.95 (m, 1H), 5.9 (m, 2H), 1.8–1.0 (m, 22H). MS (EI): *m/z* (%): 590.3 [M^+], 399.2 (100) [M^+ - PCy_2], 311.2 (14) [M^+ - PCy_2 - Cy], 227.0 (24) [M^+– 2 PCy_2 – 2Cy]. $C_{25}H_{30}BrO_2P$ (473.38): calcd. (%) C 63.43, H 6.39, found C 63.78, H 6.43.

<u>General Procedure</u>: Synthesis of mixed (dicyclohexyl/diphenylphosphino)biphenyl

At 0 °C, butyllithium (1.1 equiv) in hexanes was added dropwise to a solution of intermediate monophosphine (1 equiv) in toluene (10.0 mL for 5.50 mmol). 30 Min later, the reaction mixture was cooled to –78 °C. A solution of chlorodiphenylphosphine (1 equiv) in toluene (5.00 mL) was added dropwise. After 1 h, the reaction mixture was allowed to reach 25 °C and was then filtered through silica gel with diethyl ether as eluent. The organic layer was evaporated.

(6'-Chloro-2'-(dicyclohexylphosphino)biphenyl-2-yl)diphenylphosphine (5a): Synthesized according to the general procedure and starting from (2'-bromo-6-chlorobiphenyl-2-yl)dicyclohexylphosphine (**4a**). Purification by chromatography on silica gel afforded (6'-chloro-2'-(dicyclohexylphosphino)biphenyl-2-yl) diphenyl-phosphine (**5a**; 81%) as a colorless solid. ^{1}H NMR ($CDCl_3$, 300 MHz): δ = 7.58 (dd, J = 7.91, 1.17 Hz, 1H), 7.47–7.14 (m, 13H), 7.11–7.02 (m, 3H), 2.04–0.74 (m, 22H). MS (EI): *m/z* (%): 568.2 5 (<1) [M^+], 533.2 (<1) [M^+–Cl], 484.8 (100) [M^+–Cy_2], 402.8 [M^+–Cy]. 370.8 [M^+–PCy_2], 183.1 [M^+–PCy_2–PPh_2–Cl]. $C_{36}H_{39}P_2$ (569.10): calcd. (%) C 75.98, H 6.91, found C 76.55, H 6.82.

(6-Dicyclohexylphosphanyl-2'-diphenylphosphanylbiphenyl-2-yl)dimethylamine (5b): Synthesized according to the general procedure and starting from (2'-bromo-6-dicyclohexylphosphanylbiphenyl-2-yl)dimethylamine (**4b**). Triturating the crude product from methanol afforded (6-dicyclohexylphosphanyl-2'-diphenylphosphanyl-biphenyl-2-yl)dimethylamine (**5b,** 55%) as a white powder. mp 159–161 °C. ^{1}H NMR ($CDCl_3$, 300 MHz): δ = 7.3–7.1 (m, 11H), 7.1–7.0 (m, 5H), 6.96 (dd, J = 7.5, 1.8 Hz, 1H), 2.03 (s, 6H), 1.7–1.6 (m, 12H), 1.1–1.0 (m, 10H). ^{13}C NMR ($CDCl_3$, 75 MHz): δ = 152.8 (d, J = 2.2 Hz), 152.7 (d, J = 2.2 Hz), 144.6, 144.1 (d, J = 6.0 Hz), 139.9 (d, J = 14.8 Hz), 139.8 (d, J = 14.3 Hz), 137.7, 136.4 (d, J = 3.3 Hz), 134.1, 133.6, 133.5, 132.7 (d, J = 8.7 Hz), 127.9, 127.9, 127.9, 127.8, 127.7, 127.5, 127.4, 127.3, 127.0, 127.0, 126.8, 118.8, 43.3 (2C), 37.1 (d, J = 16.5 Hz), 35.8 (d, J = 14.3 Hz), 31.2, 31.0, 31.0, 30.7, 30.6, 30.5, 29.7, 29.7, 27.9, 27.7, 27.7, 27.5, 27.3, 27.2, 27.2, 26.9, 26.5, 26.3. ^{31}P NMR ($CDCl_3$, 161 MHz): δ = –10.2, –13.5. $C_{38}H_{45}NP$ (577.72): calcd (%) C 79.00, H 7.85 N 2.42; found C 79.31, H 8.15, N 2.20.

3'-Dicyclohexylphosphanyl-2''-diphenylphosphanyl-[1,1':2',1'']terphenyl (5d): Synthesized according to the general procedure and starting from 2''-bromo-[1,1':2',1'']terphenyl-3'-yl)dicyclohexylphosphane (**4d**). Triturating the crude product with methanol afforded 3'-dicyclohexylphosphanyl-2''-diphenylphosphanyl-[1,1':2',1'']terphenyl (**5d**, 85%) as a white powder. mp 225–227 °C. ^{1}H NMR ($CDCl_3$, 300 MHz): δ = 7.61 (d, J = 7.3 Hz, 1H), 7.5–7.0 (m, 20H), 6.58 (t, J = 6.9 Hz, 1H) 1.9–1.5 (m, 11H), 1.4–1.0 (m, 11H). ^{13}C NMR ($CDCl_3$, 75 MHz): δ = 147.6 (dd, J = 52.7, 6.7 Hz), 147.5 (dd, J = 11.8, 5.7 Hz), 142.2 (3C), 139.1 (d, J = 15.2 Hz), 137.7 (d, J = 14.3 Hz), 137.0 (d, J = 20.3 Hz), 136.1 (d, J = 2.6 Hz), 134.0 (dd, J = 7.0, 3.6 Hz), 133.8 (dd, J = 18.2, 3.0 Hz, 2C), 133.0 (d, J = 18.7 Hz, 2C), 132.0, 130.8, 130.5 (2C), 128.0 (d, J = 5.9 Hz, 2C), 127.8, 127.8 (d, J = 4.0 Hz, 2C), 127.5 (3C), 127.4, 127.3, 126.8, 125.9, 37.8 (d, J = 16.9 Hz), 13.9 (d, J = 34.6 Hz), 31.0, 30.8, 30.6, 30.4, 30.3, 28.9, 28.9, 28.8, 28.7, 28.0, 27.9, 27.9, 27.7, 27.3, 27.2, 27.2. ^{31}P NMR ($CDCl_3$, 161 MHz): δ = –10.9, –14.9. HRMS for $C_{42}H_{44}P_2$ [M+H]: calcd. 611.2991; found 611.2998.

Dicyclohexyl(4-(2-(diphenylphosphino)phenyl)benzo[*d*][1,3]dioxol-5-yl)phosphine (5e): Synthesized according to the general procedure and starting from 5-bromo-4-(2-bromophenyl)-benzo[1,3]dioxole (**4e).** Purification by chromatography on silica gel afforded dicyclohexyl(4-(2-(diphenylphosphino)phenyl) benzo[*d*][1,3]dioxol-5-yl)phosphine (**5e**; 66%) as a colorless solid. ^{1}H NMR ($CDCl_3$, 300 MHz): δ = 7.48–7.21 (m, 13H), 7.19–7.07 (m, 2H), 6.85 (d, *J* = 8.0 Hz, 1H), 5.72 (d, *J* = 1.5 Hz, 1H), 5.12 (d, *J* = 1.5 Hz, 1H), 2.0–0.8 (m, 22H). ^{13}C NMR ($CDCl_3$, 75 MHz): δ = 146.9, 137.7, 137.5, 134.4, 134.3, 134.2, 133.9, 133.4, 133.2, 128.3, 128.2, 128.1, 128.0, 127.9, 127.8, 126.7, 126.7, 107.5, 100.5, 36.3, 36.1, 34.5, 34.4, 31.2, 31.0, 30.6, 30.4, 30.196, 30.1, 30.1, 30.0, 28.9, 28.8, 27.7, 27.5, 27.5, 27.4, 27.3, 27.3, 27.1, 26.9, 26.7, 26.4, 26.3, 26.3, 26.3. ^{31}P NMR ($CDCl_3$, 161 MHz): δ = –12.2 (d, *J* = 47.2 Hz), –13.2 (d, *J* = 47.2). $C_{37}H_{40}O_2P_2$ (578.66): calcd. (%) C 76.80, H 6.97, found C 77.11, H 7.08.

Synthesis of bis(dicyclohexylphosphino)biphenyl ligands

6-Methylbiphenyl-2,2'-diyl)bis(dicyclohexylphosphine (6b): At 25 °C, butyllithium (18.4 mmol, 2 equiv) in hexanes (11.8 mL) was added dropwise to a solution of 2,2'-dibromo-6-methylbiphenyl (**1b**; 3.00 g, 9.20 mmol, 1 equiv) in toluene (47.0 mL). Then the solution was heated at 110 °C for 1 h, cooled to 50 °C and a solution of chlorodicyclohexylphosphine (4.28 g, 4.06 mL, 18.4 mmol, 2 equiv) in toluene (19 mL) was added. After 2 h, the reaction mixture was allowed to reach rt. Water (50.0 mL) was added, followed by extraction with dichloromethane (3 × 50.0 mL). The combined organic layers were dried over sodium sulfate, filtered, and evaporated. Triturating from ethylacetate afforded (6-methylbiphenyl-2,2'-diyl)bis(dicyclohexylphosphine) (**6b**; 2.82 g, 5.03 mmol, 54%) as a colorless solid. mp 150–151 °C. ^{1}H NMR (300 MHz, $CDCl_3$): δ = 7.45 (s, 1H), 7.3–7.0 (m, 5H), 6.95 (s, 1H), 1.88 (s, 3H), 1.7–1.4 (m, 22H), 1.3–0.9 (m, 22H). ^{13}C NMR (75 MHz, $CDCl_3$): δ = 148.8 (dd, *J* = 28.3, 6.8 Hz), 148.0 (dd, *J* = 28.6, 7.7 Hz), 136.5 (d, *J* = 5.6 Hz), 135.8 (d, *J* = 16.3 Hz), 135.5 (d, *J* = 16.5 Hz), 132.4, 132.1 (d, *J* = 4.2 Hz), 129.9, 129.8, 127.2, 126.2 (2C), 37.3 (d, *J* = 16.1 Hz), 34.5 (d, *J* = 15.1 Hz), 33.7–33.4 (m, 2C), 31.9–31.6 (m, 1C), 31.6–29.7 (6C), 29.1 (m, 1C), 28.1–26.5 (m, 11C), 22.0 (m, 1C). ^{31}P NMR ($CDCl_3$, 161 MHz): δ = –10.2, –10.6 (m). MS (EI): *m/z* (%): 559.4 (<1) [M^+], 477.3 (100) [M^+–Cy], 463 (15) [M^+–Cy–Me], 395.3 (15) [M^+–2 Cy], 363.3 (30) [M^+–

PCy_2], 349.3 (4) [M^+–PCy_2–Me], 311.2 (12) [M^+–3 Cy], 281.3 (6) [M^+–PCy_2–Cy], 230.1 (14) [M^+–4 Cy], 197.2 (74) [M^+–PCy_2–2 Cy], 183.1 (41) [M^+–PCy_2–2 Cy–Me]. $C_{37}H_{54}P_2$ (560.77): calcd. (%) C 79.25, H 9.71; found C 79.47, H 9.79.

(6,2'-Bis(dicyclohexylphosphanyl)biphenyl-2-yl)dimethylamine (6c): At 0 °C, butyllithium (6.40 mmol, 1.1 equiv) in hexanes (4.00 mL) was added dropwise to a solution of (2'-bromo-6-dicyclohexylphosphanylbiphenyl-2-yl)dimethylamine (**7**; 5.90 mmol, 2.67 g, 1 equiv) in toluene (12.0 mL). After 15 min the reaction mixture was cooled to –78 °C. A solution of chlorodicyclohexylphosphine (6.40 mmol, 1.50 g, 1.42 mL, 1.1 equiv) in toluene (6.00 mL) was added dropwise. After 1 h, the reaction mixture was allowed to reach 25 °C and was then filtered through silica gel with diethyl ether as eluent. The organic layer was evaporated and the crude product was crystallized from a mixture of ethyl acetate and hexane to give (6,2'-bis(dicyclohexylphosphanyl)biphenyl-2-yl)dimethylamine (**6c**; 1.44 g, 2.44 mmol, 44%) as white crystals. mp 135–137 °C. ^{1}H NMR ($CDCl_3$, 300 MHz): δ = 7.53 (s, 1H), 7.3–7.0 (m, 5H), 6.97 (d, *J* = 7.5 Hz, 1H), 2.32 (s, 6H), 2.0–1.4 (m, 26H), 1.2–0.9 (m, 22H). ^{13}C NMR ($CDCl_3$, 75 MHz): δ = 152.5 (d, *J* = 9.9 Hz), 137.4 (d, *J* = 17.0 Hz), 137.1, 136.9, 134.2 (d, *J* = 12.0 Hz), 134.1, 132.6, 127.2, 126.7, 126.3, 126.1, 118.4, 37.9 (2C), 35.5 (d, *J* = 14.8 Hz), 35.2 (d, *J* = 15.9 Hz), 34.9 (d, *J* = 14.8 Hz), 31.6, 31.4, 31.3, 31.0 (m), 30.0 (m), 29.7, 28.2 (m), 28.1 (m), 27.8, 27.7, 27.6, 27.4 (m), 27.3, 27.3, 27.2 (m), 26.7, 26.6, 26.6, 26.5. ^{31}P NMR ($CDCl_3$, 161 MHz): δ = –8.3, –9.6. $C_{38}H_{57}NP_2$ (589.81): calcd. (%) C 77.38, H 9.74, N 2.37; found C 77.51, H 9.79, N 2.13.

[1,1':2',1'']Terphenyl-3',2''-yl)bis(dicyclohexylphosphane) (6f): At –78 °C, *tert*-butyllithium (24.0 mmol, 4 equiv) in pentane was added dropwise to a solution of 2,2'-dibromo-6-phenylbiphenyl (**1f**; 6.00 mmol, 2.32 g, 1 equiv) in THF (12.0 mL). After 1 h, a solution of chlorodicyclohexylphosphine (2.65 mL, 2.79 g, 12.0 mmol, 2 equiv) in toluene (10.0 mL) was added dropwise. After 1 h, the mixture was allowed to reach 25 °C and filtered on silica gel with diethyl ether. The crude product was purified by chromatography which afforded [1,1';2',1'']terphenyl-3',2''-yl)bis(dicyclohexyl-phosphane) (**6f**; 1.87 g, 3.97 mmol, 66%) as a colorless powder. mp 148–150 °C. ^{1}H NMR ($CDCl_3$, 300 MHz): δ = 7.4–7.0 (m, 12H), 2.0–1.4 (m, 21H), 1.4–1.0, 22H). ^{13}C NMR ($CDCl_3$, 100 MHz): δ = 147.8 (dd, *J* = 31.2, 5.6 Hz), 147.7 (dd, *J* = 31.2,

6.9 Hz), 142.8, 142.0 (d, J = 2.5 Hz), 137.6 (d, J = 19.3 Hz), 136.3 (d, J = 17.5 Hz), 134.9 (dd, J = 6.2, 4.3 Hz), 133.2, 131.3, 131.0 (3C), 130.3, 128.0 (2C), 127.0 (d, J = 18.1 Hz), 126.5, 126.3, 38.4 (d, J = 16.9), 34.9 (m, 2C), 34.0 (d, J = 15.6 Hz), 32.6, 32.5, 32.4, 32.4, 31.9, 31.8, 31.3, 31.3, 31.2, 30.7, 30.6, 30.3, 30.2, 29.8, 29.7, 29.6, 29.5, 28.7, 28.6, 28.3, 28.2, 27.9, 27.6, 27.1, 27.1, 26.9, 26.8, 26.7. ^{31}P NMR ($CDCl_3$, 161 MHz): δ = –9.7, –10.1. HRMS for $C_{42}H_{57}BrP_2$: calcd. 623.3930; found 623.3872.

(6-Chlorobiphenyl-2,2'-diyl)bis(dicyclohexylphosphine) (6g): At –75 °C, butyllithium (15.4 mmol, 2 equiv) in hexanes (9.88 mL) was added dropwise to a solution of 2,2'-dibromo-6-chlorobiphenyl (**1g**; 2.67 g, 7.70 mmol, 1 equiv) in THF (40.0 mL). After 1 h, a solution of chlorodicyclohexylphosphine (3.59 g, 3.40 mL, 15.4 mmol, 2 equiv) was added. After 1 h, the reaction mixture was allowed to reach rt. Water (50.0 mL) was added followed by extraction with dichloromethane (3 × 50.0 mL). The combined organic layers were dried over sodium sulfate, filtered, and evaporated. The crude product was purified by chromatography on silica gel with cyclohexane as eluent to afford (6-chlorobiphenyl-2,2'-diyl)bis(dicyclohexyl-phosphine) (**6g**; 2.20 g, 3.79 mmol, 49%) as a colorless solid. mp 163–164 °C. ^{1}H NMR (300 MHz, $CDCl_3$): δ = 7.5–7.4 (m, 1H), 7.4–7.3 (m, 3H), 7.3–7.2 (m, 1H), 7.0–6.9 (m, 2H), 1.7–1.3 (m, 22H), 1.3–0.8 (m, 22H). ^{13}C NMR (75 MHz, $CDCl_3$): δ = 147.4 (d, J = 35.1 Hz), 146.0 (d, J = 30.2 Hz), 138.9 (d, J = 23.3 Hz), 136.3 (d, J = 17.4 Hz), 134.8 (dd, J = 7.7, 1.8 Hz), 132.4, 132.3, 130.8, 129.4, 127.5, 127.2, 126.8, 37.1 (d, J = 17.8 Hz), 35.0 (d, J = 16.2 Hz), 33.5 (d, J = 15.4 Hz), 33.2 (d, J = 15.3 Hz), 30.6–29.4 (m, 5C), 28.0–26.4 (15C). ^{31}P NMR ($CDCl_3$, 161 MHz): δ = –11.3 (d, J = 22.6 Hz), –13.5 (d, J = 22.6 Hz). MS (EI): *m/z* (%) 580.3 (<1) [M^+], 543.3 (15) [M^+–Cl], 497.3 (100) [M^+–Cy], 415.2 (14) [M^+–2 Cy], 217.1 (56) [M^+–PCy_2–2 Cy], 183.2 [M^+–PCy_2–2 Cy–Cl]. $C_{36}H_{51}P_2Cl$ (581.19): calcd. (%) C 74.40, H 8.84, found C 74.73, H 8.92.

Dicyclohexyl(2-(5-(dicyclohexylphosphino)benzo[*d*][1,3]dioxol-4-yl)phenyl)phosphine (6h): At 25 °C, butyllithium (22.5 mmol, 2 equiv) in hexanes (14.4 mL) was added dropwise to a solution of 5-bromo-4-(2-bromophenyl)benzo[*d*][1,3]dioxole (**1h**; 4.00 g, 11.2 mmol, 1 equiv) in toluene (57.0 mL). Then, the solution was heated at 110 °C for 1 h. Then, it was cooled at 50 °C and a solution of chlorodicyclohexylphosphine (5.23 g, 4.96 mL, 22.5 mmol,

2 equiv) in toluene (23.0 mL) was added. After 2 h, the reaction mixture was allowed to reach rt. Water (60.0 mL) was added followed by extraction with dichloromethane (3 × 60.0 mL). The combined organic layers were dried over sodium sulfate, filtered, and evaporated. Triturating from ethylacetate afforded dicyclohexyl(2-(5-(dicyclohexylphosphino)benzo[*d*][1,3]dioxol-4-yl)phenyl)phosphine (**6h**; 4.59 g, 7.77 mmol, 70%) as colorless solid. mp 219–220 °C. ^{1}H NMR (300 MHz, $CDCl_3$): δ = 7.46 (d, *J* = 2.3 Hz, 1H), 7.3–7.2 (m, 2H), 7.1–7.0 (m, 1H), 6.96 (d, *J* = 7.2 Hz, 1H), 6.78 (d, *J* = 7.9 Hz, 1H), 5.86 (s, 1H), 5.72 (s, 1H), 1.9–1.4 (m, 22H), 1.4–0.9 (m, 22H). ^{13}C NMR (75 MHz, $CDCl_3$): δ = 145.6 (d, *J* = 11.8 Hz), 143.2 (dd, *J* = 31.1, 5.5 Hz), 136.3 (d, *J* = 19.2 Hz), 132.6 (d, *J* = 3.0 Hz), 132.0 (dd, *J* = 6.0, 2.1 Hz), 131.5 (dd, *J* = 35.6, 6.0 Hz), 128.3 (d, *J* = 19.2 Hz), 127.6, 127.0, 126.5, 107.4, 100.5, 36.9 (d, *J* = 16.4 Hz), 35.7 (d, *J* = 16.1 Hz), 34.4 (d, *J* = 13.9 Hz), 33.9 (d, *J* = 14.3 Hz), 31.0–29.7 (m, 7C), 29.2 (m, 1C), 27.9–26.8 (m, 7C), 26.8–26.5 (m, 5C). ^{31}P NMR ($CDCl_3$, 161 MHz): δ = –10.2 (d, *J* = 18.0 Hz), –12.8 (d, *J* = 18.0 Hz). MS (EI): *m/z* (%) 589.4 (< 2) [M^+], 507.4 (100) [M^+–Cy], 425.2 (28) [M^+–2 Cy], 393.3 (22) [M^+–PCy_2], 341.2 (17) [M^+–3 Cy], 260.1 (26) [M^+–4 Cy], 227.1 (77) [M^+–PCy_2–2 Cy], 199.1 (35) [M^+–2 PCy_2]. $C_{37}H_{52}O_2P_2$ (590.76): calcd. (%) C 75.23, H 8.87; found C 75.66, H 9.02.

Dicyclohexyl(2-(5-(dicyclohexylphosphino)-2,2-difluorobenzo[*d*][1,3]dioxol-4-yl)phenyl)phosphine (6i): At 25 °C, butyllithium (27.3 mmol, 3 equiv) in hexanes (17.5 mL) was added dropwise to a solution of 5-bromo-2,2-difluoro-4-(2-iodophenyl)benzo[*d*][1,3]dioxole (**1i**; 4.00 g, 9.11 mmol, 1 equiv) in toluene (46.0 mL). After 1 h, a solution of chlorodicyclohexylphosphine (6.36 g, 6.00 mL, 27.3 mmol, 3 equiv) in toluene (30.0 mL) was added. After 2 h, water (70.0 mL) was added followed by extraction with dichloromethane (3 × 70.0 mL). The combined organic layers were dried over sodium sulfate, filtered, and evaporated. The crude product was purified by chromatography on silica gel. Triturating from ethyl acetate afforded pure dicyclohexyl(2-(5-(dicyclohexylphosphino)-2,2-difluorobenzo[*d*][1,3]dioxol-4-yl)phenyl)phosphine (**6i**; 3.00 g, 5.25 mmol, 53%) as a colorless solid. mp 194–195 °C. ^{1}H NMR (300 MHz, $CDCl_3$): δ = 7.5–7.4 (m, 1H), 7.3–7.2 (m, 2H), 7.2–7.1 (m, 1H), 7.1–7.0 (m, 1H), 6.99 (d, *J* = 8.2 Hz, 1H), 1.8–1.4 (m, 22H), 1.3–0.8 (m, 22H). ^{13}C NMR (75 MHz, $CDCl_3$): δ = 143.2, 142.4 (d, *J* = 11.7 Hz), 141.3 (dd, *J* = 31.0, 5.3 Hz), 136.1 (d, *J* = 20.2 Hz), 132.9 (2C), 132.0 (dd, *J* = 6.2, 2.1 Hz), 127.9

(d, *J* = 2.9 Hz), 127.7 (2C), 127.5, 107.7, 36.8 (d, *J* = 16.9 Hz), 35.6 (d, *J* = 15.3 Hz), 34.0 (d, *J* = 14.6 Hz), 33.7 (d, *J* = 14.1 Hz), 31.0–30.0 (m, 6C), 29.3–29.1 (m, 2C), 27.7 (m, 8C), 26.8 (s, 2C), 26.4 (s, 2C). ^{31}P NMR ($CDCl_3$, 161 MHz): δ = –10.3 (d, *J* = 22.5 Hz), –12.2 (d, *J* = 22.3 Hz). MS (EI): *m/z* (%) 626.3 (< 1) [M^+], 543.3 (100) [M^+–Cy], 461.2 (17) [M^+–2 Cy], 295.1 (14) [M^+–4 Cy], 263 (63) [M^+–PCy_2–2 Cy].

Dicyclohexyl(2-(6-(dicyclohexylphosphino)-2,2,3,3-tetrafluoro-2,3-dihydrobenzo[*b*][1,4]dioxin-5-yl)phenyl)phosphine (6j): At 25 °C, butyllithium (12.3 mmol, 3 equiv) in hexanes (7.87 mL) was added dropwise to a solution of 6-bromo-2,2,3,3-tetrafluoro-5-(2-iodophenyl)-2,3-dihydrobenzo[*b*][1,4]dioxine (**1j**; 2.00 g, 4.09 mmol, 1 equiv) in toluene (20.0 mL). After 1 h, a solution of chlorodicyclohexylphosphine (2.86 g, 2.71 mL, 12.3 mmol, 3 equiv) in toluene (13.0 mL) was added. After 2 h, water (30.0 mL) was added followed by extraction with dichloromethane (3 × 30.0 mL). The combined organic layers were dried over sodium sulfate, filtered, and evaporated. The crude product was purified by chromatography on silica gel. Triturating from methanol afforded dicyclohexyl(2-(6-(dicyclohexylphosphino)-2,2,3,3-tetrafluoro-2,3-dihydrobenzo[*b*][1,4]dioxin-5-yl)phenyl)phosphine (**6j**; 2.00 g, 2.96 mmol, 73%) as colorless crystals. ^{1}H NMR (300 MHz, $CDCl_3$): δ = 7.49 (d, *J* = 5.1 Hz, 1H), 7.4–7.2 (m, 3H), 7.06 (d, *J* = 8.4 Hz,1H), 7.0–6.9 (m, 1H), 1.9–1.3 (m, 22H), 1.2–0.8 (m, 22H). ^{13}C NMR (75 MHz, $CDCl_3$): δ = 141.5 (d, *J* = 30.6 Hz), 137.33 (d, *J* = 1.9 Hz, 2C), 135.25 (d, *J* = 9.9 Hz, 2C), 132.9, 132.37 (d, *J* = 6.5 Hz), 129.0, 127.7, 127.4, 115.6, 36.7 (d, *J* = 17.2 Hz), 35.6–35.3 (m), 33.6–33.4 (m), 31.3–29.4 (m), 27.7–26.3 (m). ^{31}P NMR ($CDCl_3$, 161 MHz): δ = –10.0, –12.0. MS (EI): *m/z* (%) 676.74 (< 1) [M^+], 593.3 (100) [M^+–Cy], 511.2 (16) [M^+–2 Cy], 427.1 (10) [M^+–3 Cy], 313.1 (69) [M^+–P–4 Cy].

6-Fluoro-2,2'-bis(dicyclohexylphosphino)biphenyl (6k): 2,2’-Dibromo-6-fluorobiphenyl (**1k**; 1.60 g, 5.00 mmol) was added to a solution of butyllithium (10.0 mmol) in hexanes (6.30 mL) and THF (20.0 mL) at –78 °C. After 15 min at –75 °C, a solution of chlorodicyclohexylphosphine (2.00 mL, 2.00 g, 10.0 mmol) in THF (10.0 mL) was added to the reaction mixture. Evaporation of the solvent followed by column chromatography on silica gel with cyclohexane as eluent gave a colorless oil. Crystallization from ethyl acetate/hexanes (1:5) afforded 6-fluoro-2,2'-bis(dicyclohexylphosphino)biphenyl (**6k**; 2.23 g, mmol, 79%) as colorless needles.

mp 184–186 °C. ^{1}H NMR (400 MHz, $CDCl_3$): δ = 7.5 (m, 1H), 7.3 (m, 4H), 7.1 (m, 1H), 1.7 (m, 24H), 1.2 (m, 20H). ^{13}C NMR (101 MHz, [D8]toluene): δ = 162.7 (d, J = 11 Hz), 160.2 (d, J = 10 Hz), 143.8 (d, J = 5 Hz), 143.8 (d, J = 5.0 Hz), 139.6 (d, J = 22 Hz), 137.1 (d, J = 20 Hz), 133.6 (d, J = 5 Hz), 133.4, 133.1, 128.5 (d, J = 22 Hz), 128.2 (d, J = 12 Hz), 115.9 (d, J = 24 Hz), 37.8 (d, J = 18 Hz), 36.8 (d, J = 17 Hz), 34.6 (d, J = 17 Hz), 34.4 (d, J = 17 Hz), 31 (m), 30.9 (d, J = 14 Hz), 30.5 (d, J = 9 Hz), 30.4 (d, J = 8 Hz), 28 (m), 27.4. $C_{36}H_{51}FP_2$ (564.75): calcd. (%) C 76.56, H 9.10; found C 76.43, H 9.04.

(6-(Trifluoromethoxy)biphenyl-2,2'-diyl)bis(dicyclohexylphosphine) (6l): At 25 °C, butyllithium (6.96 mmol, 2 equiv) in hexanes (4.46 mL) was added dropwise to a solution of 2,2'-dibromo-6-(trifluoromethoxy)biphenyl (**1l**; 1.38 g, 3.48 mmol, 1 equiv) in toluene (18.0 mL). Then the solution was heated at 110 °C for 1 h. Then, it was cooled at 50 °C and a solution of chlorodicyclohexylphosphine (1.62 g, 1.54 mL, 6.96 mmol, 2 equiv) in toluene (7.00 mL) was added. After 2 h, the reaction mixture was allowed to reach rt. Water (30.0 mL) was added followed by extraction with dichloromethane (3 × 30.0 mL). The combined organic layers were dried over sodium sulfate, filtered, and evaporated. Triturating from methanol afforded (6-(trifluoromethoxy)biphenyl-2,2'-diyl)bis(dicyclohexylphosphine) (**6l**; 0.72 g, 1.14 mmol, 33%) as colorless crystals. ^{1}H NMR (300 MHz, $CDCl_3$): δ = 7.48 (d, J = 4.48 Hz, 1H), 7.4–7.2 (m, 5H), 6.98 (d, J = 6.68 Hz, 1H), 1.8–1.4 (m, 22H), 1.3–0.9 (m, 22H). ^{13}C NMR (75 MHz, $CDCl_3$): δ = 147.2 (d, J = 9.7 Hz), 132.4, 130.4, 127.6, 127.1, 122.1 (t, J = 260.0 Hz), 37.2 (d, J = 16.4 Hz), 35.3, 34.1, 33.9, 31.5–29.6 (m), 27.9–26.4 (m). ^{31}P NMR ($CDCl_3$, 161 MHz): δ = –9.6, –10.5. $C_{37}H_{51}F_3OP_2$ (630.34): calcd. (%) C 70.46, H 8.15; found C 70.93, H 8.32.

General procedure for the synthesis of bis(diphenylphosphines)biphenyls 1 by modification of the solvent: At 25 °C, BuLi (2 or 3 equiv) in hexanes was added dropwise to a solution of 2,2'-dihalobiphenyl (1 equiv) in toluene. The reaction mixture was heated at 110 °C. After 1 h it was cooled to 50 °C and a solution of $ClPPh_2$ (2 or 3 equiv) in toluene (1 mmol in 5 mL) was quickly added. The solution was heated for 2.5 h at 110 °C and was then allowed to reach rt. Water was immediately added followed by extraction with dichloromethane. The combined

organic layers were dried over sodium sulfate and solvents were evaporated under reduce pressure.

References

1. Schlosser, M. In *Organometallics in Synthesis : A Manual*; 2nd ed.; Schlosser, M., Ed.; Wiley: Chichester, 2002, p 1-352.

2. Bonnafoux, L.; Gramage-Doria, R.; Colobert, F.; Leroux, F. R. *Chem. Eur. J.* **2011**, doi: 10.1002/chem.201101529.

3. Leroux, F. R.; Mettler, H. *Adv. Synth. Catal.* **2007**, *349*, 323-336.

4. Bonnafoux, L.; Ernst, L.; Leroux, F. R.; Colobert, F. *Eur. J. Inorg. Chem.* **2011**, 3387-3397.

5. Bonnafoux, L.; Colobert, F.; Leroux, F. R. *Synlett* **2010**, 2953-2955.

6. Ollivier, C.; Renaud, P. *J. Am. Chem. Soc.* **2001**, *123*, 4717-4727.
