# Supplementary material for: Bromine–lithium exchange: An efficient tool in the modular construction of biaryl ligands
Source: Beilstein J Org Chem. 2011 Sep 14;7:1278–87. doi: 10.3762/bjoc.7.148 (PMC3182437; doi:10.3762/bjoc.7.148)
Supplement: File 2 — Crystal structure data for 3a. [file Beilstein_J_Org_Chem-07-1278-s002.pdf]

Supporting Information

for

Bromine–lithium exchange: An efficient tool in the modular construction of biaryl ligands

Laurence Bonnafoux, Frédéric R. Leroux* and Françoise Colobert*

Address: Laboratoire de stéréochimie, UMR 7509, CNRS-Université de Strasbourg, ECPM, 25 rue Becquerel, F-67087 Strasbourg Cedex 02, France

Email: Laurence Bonnafoux - laurence.bonnafoux@gmail.com; Frédéric R. Leroux* - frederic.leroux@unistra.fr; Françoise Colobert* - francoise.colobert@unistra.fr

* Corresponding author

Crystal structure data for 3a.
CCDC 827188

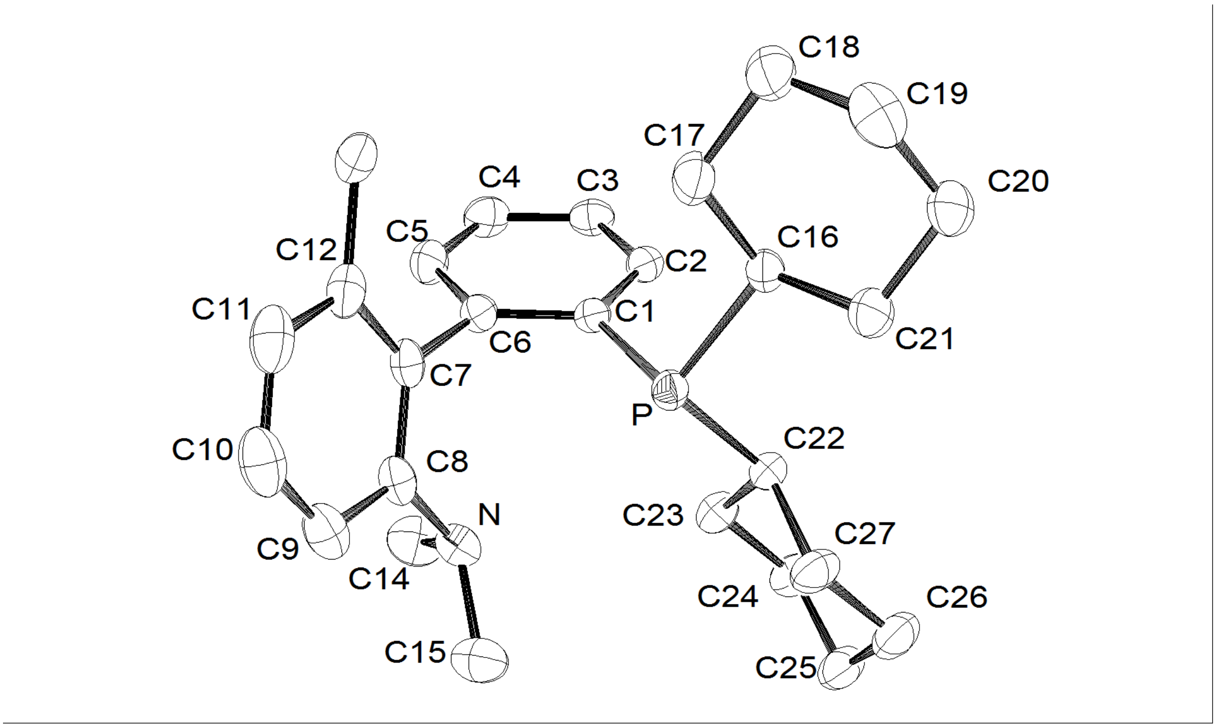

Crystal data

$C_{27}H_{38}NP$	
M_r = 407.55	D_x = 1.144 Mg m^{-3}
Monoclinic, $P2_1/c$	Melting point: ? K
Hall symbol: -P 2ybc	Mo $K\alpha$ radiation, λ = 0.71073 Å
a = 13.2100 (2) Å	Cell parameters from 6697 reflections
b = 10.0050 (2) Å	θ = 1.0–30.0°
c = 18.7160 (3) Å	μ = 0.13 mm^{-1}
β = 106.9320 (12)°	T = 173 K
V = 2366.39 (7) Å^3	Prism, colorless
Z = 4	0.20 × 0.20 × 0.20 mm
F(000) = 888	

Data collection

KappaCCD diffractometer	5101 reflections with $I > 2\sigma(I)$
Radiation source: fine-focus sealed tube	R_{int} = 0.035

graphite	θ_{max} = 30.0°, θ_{min} = 2.3°
Detector resolution: ? pixels mm^{-1}	$h = -18 \rightarrow 18$
π scans	$k = -14 \rightarrow 13$
12805 measured reflections	$l = -26 \rightarrow 26$
6902 independent reflections	

Refinement

Refinement on F^2	Secondary atom site location: difference Fourier map
Least-squares matrix: full	Hydrogen site location: inferred from neighbouring sites
$R[F^2 > 2\sigma(F^2)]$ = 0.050	H-atom parameters constrained
$wR(F^2)$ = 0.123	$w = 1/[\sigma^2(F_o^2) + (0.0501P)^2 + 0.7481P]$ where $P = (F_o^2 + 2F_c^2)/3$
S = 1.03	$(\Delta/\sigma)_{max}$ = 0.003
6902 reflections	$\Delta\rho_{max}$ = 0.28 e Å^{-3}
262 parameters	$\Delta\rho_{min}$ = −0.38 e Å^{-3}
0 restraints	Extinction correction: none
? constraints	Extinction coefficient: ?
Primary atom site location: structure-invariant direct methods	

Refinement of F^2 against ALL reflections. The weighted *R*-factor *wR* and goodness of fit S are based on F^2, conventional *R*-factors *R* are based on F, with F set to zero for negative F^2. The threshold expression of $F^2 > 2sigma(F^2)$ is used only for calculating *R*-factors(gt), etc. and is not relevant to the choice of reflections for refinement. *R*-factors based on F^2 are statistically about twice as large as those based on F, and *R*– factors based on ALL data will be even larger.

Fractional atomic coordinates and isotropic or equivalent isotropic displacement parameters (Å^2)

	x	*y*	*z*	U_{iso}*/U_{eq}
P	0.75931 (3)	0.21632 (4)	0.136582 (18)	0.02309 (10)

N	0.93783 (10)	0.46404 (13)	0.18200 (7)	0.0305* (3)
C1	0.73843 (10)	0.32748 (13)	0.05496 (7)	0.0216 (3)
C2	0.63675 (10)	0.36664 (14)	0.01305 (7)	0.0252 (3)
H2	0.5774	0.3330	0.0263	0.030*
C3	0.62021 (11)	0.45282 (15)	−0.04698 (7)	0.0290 (3)
H3	0.5504	0.4783	−0.0742	0.035*
C4	0.70601 (13)	0.50173 (16)	−0.06714 (8)	0.0326 (3)
H4	0.6954	0.5619	−0.1079	0.039*
C5	0.80743 (12)	0.46259 (16)	−0.02757 (8)	0.0315 (3)
H5	0.8660	0.4953	−0.0421	0.038*
C6	0.82525 (11)	0.37555 (14)	0.03353 (7)	0.0243 (3)
C7	0.93676 (11)	0.33210 (14)	0.07210 (8)	0.0268 (3)
C8	0.99034 (11)	0.37689 (14)	0.14449 (8)	0.0283 (3)
C9	1.09366 (12)	0.33211 (17)	0.17868 (9)	0.0368 (4)
H9	1.1306	0.3620	0.2275	0.044*
C10	1.14181 (13)	0.24525 (18)	0.14198 (11)	0.0424 (4)
H10	1.2117	0.2151	0.1659	0.051*
C11	1.09037 (13)	0.20132 (17)	0.07121 (11)	0.0416 (4)
H11	1.1251	0.1415	0.0466	0.050*
C12	0.98768 (12)	0.24365 (16)	0.03508 (9)	0.0334 (3)
C13	0.93261 (12)	0.18606 (17)	−0.04340 (9)	0.0354 (3)
H13A	0.9787	0.1193	−0.0563	0.053*
H13B	0.9186	0.2584	−0.0803	0.053*
H13C	0.8657	0.1440	−0.0433	0.053*
C14	0.92627 (15)	0.60025 (17)	0.15370 (10)	0.0434 (4)
H14A	0.9962	0.6422	0.1645	0.065*
H14B	0.8821	0.6513	0.1779	0.065*

H14C	0.8928	0.5991	0.0996	0.065*
C15	0.97877 (14)	0.46505 (19)	0.26340 (9)	0.0436 (4)
H15A	0.9847	0.3730	0.2821	0.065*
H15B	0.9304	0.5154	0.2843	0.065*
H15C	1.0487	0.5074	0.2784	0.065*
C16	0.67368 (10)	0.07300 (14)	0.09052 (7)	0.0240 (3)
H16	0.6042	0.1098	0.0598	0.029*
C17	0.72574 (13)	0.00130 (17)	0.03797 (9)	0.0374 (4)
H17A	0.7303	0.0638	−0.0020	0.045*
H17B	0.7987	−0.0246	0.0663	0.045*
C18	0.66472 (13)	−0.12347 (17)	0.00245 (10)	0.0400 (4)
H18A	0.5947	−0.0968	−0.0309	0.048*
H18B	0.7039	−0.1693	−0.0282	0.048*
C19	0.64956 (13)	−0.21898 (16)	0.06152 (10)	0.0401 (4)
H19A	0.6077	−0.2972	0.0371	0.048*
H19B	0.7193	−0.2514	0.0925	0.048*
C20	0.59280 (13)	−0.14949 (16)	0.11077 (9)	0.0361 (3)
H20A	0.5858	−0.2121	0.1500	0.043*
H20B	0.5208	−0.1239	0.0803	0.043*
C21	0.65260 (12)	−0.02444 (16)	0.14752 (8)	0.0336 (3)
H21A	0.6108	0.0215	0.1763	0.040*
H21B	0.7209	−0.0516	0.1830	0.040*
C22	0.67366 (11)	0.29620 (15)	0.18947 (7)	0.0256 (3)
H22	0.5990	0.2660	0.1667	0.031*
C23	0.67538 (12)	0.44958 (15)	0.19024 (8)	0.0297 (3)
H23A	0.7493	0.4809	0.2106	0.036*
H23B	0.6479	0.4832	0.1384	0.036*

C24	0.60852 (13)	0.50650 (17)	0.23750 (8)	0.0362 (4)
H24A	0.6144	0.6052	0.2387	0.043*
H24B	0.5333	0.4831	0.2141	0.043*
C25	0.64341 (14)	0.45302 (17)	0.31680 (8)	0.0372 (4)
H25A	0.7153	0.4865	0.3426	0.045*
H25B	0.5948	0.4862	0.3443	0.045*
C26	0.64402 (15)	0.30151 (18)	0.31764 (8)	0.0403 (4)
H26A	0.5706	0.2681	0.2978	0.048*
H26B	0.6725	0.2696	0.3697	0.048*
C27	0.71140 (13)	0.24585 (16)	0.27058 (8)	0.0346 (3)
H27A	0.7082	0.1470	0.2708	0.041*
H27B	0.7860	0.2727	0.2932	0.041*

Atomic displacement parameters (Å^2)

	U^{11}	U^{22}	U^{33}	U^{12}	U^{13}	U^{23}
P	0.02061 (17)	0.02650 (18)	0.02172 (17)	−0.00037 (14)	0.00548 (12)	0.00384 (14)
N	0.0283 (6)	0.0280 (6)	0.0321 (6)	−0.0014 (5)	0.0037 (5)	−0.0019 (5)
C1	0.0230 (6)	0.0224 (6)	0.0193 (6)	−0.0009 (5)	0.0062 (5)	−0.0009 (5)
C2	0.0231 (6)	0.0300 (7)	0.0227 (6)	−0.0004 (5)	0.0068 (5)	−0.0016 (5)
C3	0.0299 (7)	0.0308 (8)	0.0228 (6)	0.0050 (6)	0.0021 (5)	0.0005 (6)
C4	0.0411 (9)	0.0297 (8)	0.0250 (7)	0.0009 (6)	0.0066 (6)	0.0062 (6)
C5	0.0334 (8)	0.0319 (8)	0.0310 (7)	−0.0056 (6)	0.0124 (6)	0.0048 (6)
C6	0.0253 (7)	0.0235 (7)	0.0245 (6)	−0.0025 5)	0.0082 (5)	−0.0010 (5)
C7	0.0232 (7)	0.0250 (7)	0.0341 (7)	−0.0033 (5)	0.0115 (5)	0.0059 (6)

C8	0.0229 (7)	0.0261 (7)	0.0361 (7)	−0.0029 (6)	0.0089 (6)	0.0041 (6)
C9	0.0259 (7)	0.0375 (9)	0.0439 (9)	−0.0020 (6)	0.0051 (6)	0.0061 (7)
C10	0.0263 (8)	0.0390 (9)	0.0622 (11)	0.0040 (7)	0.0133 (7)	0.0113 (8)
C11	0.0342 (8)	0.0351 (9)	0.0635 (11)	0.0039 (7)	0.0270 (8)	0.0029 (8)
C12	0.0313 (8)	0.0316 (8)	0.0434 (9)	−0.0022 (6)	0.0206 (7)	0.0019 (6)
C13	0.0345 (8)	0.0413 (9)	0.0367 (8)	−0.0025 (7)	0.0199 (7)	−0.0027 (7)
C14	0.0520 (10)	0.0281 (8)	0.0469 (10)	0.0018 (7)	0.0093 (8)	−0.0020 (7)
C15	0.0425 (10)	0.0492 (11)	0.0338 (8)	−0.0007 (8)	0.0026 (7)	−0.0046 (8)
C16	0.0218 (6)	0.0255 (7)	0.0245 (6)	−0.0008 (5)	0.0066 (5)	0.0030 (5)
C17	0.0380 (9)	0.0353 (9)	0.0471 (9)	−0.0072 (7)	0.0252 (7)	−0.0082 (7)
C18	0.0410 (9)	0.0358 (9)	0.0507 (10)	−0.0048 (7)	0.0249 (8)	−0.0118 (7)
C19	0.0338 (8)	0.0249 (8)	0.0594 (10)	0.0029 (6)	0.0101 (7)	0.0002 (7)
C20	0.0429 (9)	0.0290 (8)	0.0375 (8)	−0.0076 (7)	0.0132 (7)	0.0061 (6)
C21	0.0375 (8)	0.0327 (8)	0.0302 (7)	−0.0067 (6)	0.0092 (6)	0.0053 (6)
C22	0.0259 (7)	0.0304 (7)	0.0213 (6)	−0.0032 (6)	0.0081 (5)	0.0002 (5)
C23	0.0342 (8)	0.0311 (8)	0.0256 (7)	0.0035 (6)	0.0115 (6)	0.0040 (6)
C24	0.0422 (9)	0.0377 (9)	0.0303 (7)	0.0077 (7)	0.0131 (6)	0.0011 (6)
C25	0.0478 (10)	0.0397 (9)	0.0285 (7)	−0.0011 (7)	0.0181 (7)	−0.0024 (7)
C26	0.0576 (11)	0.0401 (9)	0.0288 (7)	−0.0068 (8)	0.0214 (7)	0.0009 (7)
C27	0.0477 (9)	0.0330 (8)	0.0241 (7)	−0.0008 (7)	0.0122 (6)	0.0039 (6)

Geometric parameters (Å, °)

P—C1	1.8442 (13)	C15—H15C	0.9800
P—C16	1.8738 (14)	C16—C21	1.5294 (19)
P—C22	1.8843 (14)	C16—C17	1.532 (2)
N—C8	1.4206 (19)	C16—H16	1.0000
N—C14	1.454 (2)	C17—C18	1.529 (2)
N—C15	1.461 (2)	C17—H17A	0.9900
C1—C2	1.3997 (18)	C17—H17B	0.9900
C1—C6	1.4055 (18)	C18—C19	1.518 (2)
C2—C3	1.3822 (19)	C18—H18A	0.9900
C2—H2	0.9500	C18—H18B	0.9900
C3—C4	1.384 (2)	C19—C20	1.516 (2)
C3—H3	0.9500	C19—H19A	0.9900
C4—C5	1.385 (2)	C19—H19B	0.9900
C4—H4	0.9500	C20—C21	1.532 (2)
C5—C6	1.4017 (19)	C20—H20A	0.9900
C5—H5	0.9500	C20—H20B	0.9900
C6—C7	1.5036 (19)	C21—H21A	0.9900
C7—C8	1.407 (2)	C21—H21B	0.9900
C7—C12	1.410 (2)	C22—C23	1.535 (2)
C8—C9	1.402 (2)	C22—C27	1.5382 (19)
C9—C10	1.373 (2)	C22—H22	1.0000
C9—H9	0.9500	C23—C24	1.530 (2)
C10—C11	1.373 (3)	C23—H23A	0.9900
C10—H10	0.9500	C23—H23B	0.9900
C11—C12	1.394 (2)	C24—C25	1.518 (2)
C11—H11	0.9500	C24—H24A	0.9900

C12—C13	1.548 (2)	C24—H24B	0.9900
C13—H13A	0.9800	C25—C26	1.516 (2)
C13—H13B	0.9800	C25—H25A	0.9900
C13—H13C	0.9800	C25—H25B	0.9900
C14—H14A	0.9800	C26—C27	1.528 (2)
C14—H14B	0.9800	C26—H26A	0.9900
C14—H14C	0.9800	C26—H26B	0.9900
C15—H15A	0.9800	C27—H27A	0.9900
C15—H15B	0.9800	C27—H27B	0.9900
C1—P—C16	98.96 (6)	C18—C17—C16	112.52 (12)
C1—P—C22	102.21 (6)	C18—C17—H17A	109.1
C16—P—C22	101.06 (6)	C16—C17—H17A	109.1
C8—N—C14	114.19 (13)	C18—C17—H17B	109.1
C8—N—C15	115.72 (13)	C16—C17—H17B	109.1
C14—N—C15	109.95 (13)	H17A—C17—H17B	107.8
C2—C1—C6	118.29 (12)	C19—C18—C17	111.21 (14)
C2—C1—P	121.37 (10)	C19—C18—H18A	109.4
C6—C1—P	120.34 (10)	C17—C18—H18A	109.4
C3—C2—C1	121.92 (13)	C19—C18—H18B	109.4
C3—C2—H2	119.0	C17—C18—H18B	109.4
C1—C2—H2	119.0	H18A—C18—H18B	108.0
C2—C3—C4	119.61 (13)	C20—C19—C18	110.35 (13)
C2—C3—H3	120.2	C20—C19—H19A	109.6
C4—C3—H3	120.2	C18—C19—H19A	109.6
C3—C4—C5	119.71 (13)	C20—C19—H19B	109.6
C3—C4—H4	120.1	C18—C19—H19B	109.6
C5—C4—H4	120.1	H19A—C19—H19B	108.1

C4—C5—C6	121.21 (13)	C19—C20—C21	111.47 (13)
C4—C5—H5	119.4	C19—C20—H20A	109.3
C6—C5—H5	119.4	C21—C20—H20A	109.3
C5—C6—C1	119.23 (12)	C19—C20—H20B	109.3
C5—C6—C7	118.65 (12)	C21—C20—H20B	109.3
C1—C6—C7	122.07 (12)	H20A—C20—H20B	108.0
C8—C7—C12	119.68 (13)	C16—C21—C20	112.47 (12)
C8—C7—C6	121.20 (13)	C16—C21—H21A	109.1
C12—C7—C6	119.12 (13)	C20—C21—H21A	109.1
C9—C8—C7	119.19 (14)	C16—C21—H21B	109.1
C9—C8—N	121.42 (13)	C20—C21—H21B	109.1
C7—C8—N	119.37 (12)	H21A—C21—H21B	107.8
C10—C9—C8	120.35 (15)	C23—C22—C27	108.58 (12)
C10—C9—H9	119.8	C23—C22—P	114.80 (9)
C8—C9—H9	119.8	C27—C22—P	107.94 (10)
C11—C10—C9	120.92 (15)	C23—C22—H22	108.5
C11—C10—H10	119.5	C27—C22—H22	108.5
C9—C10—H10	119.5	P—C22—H22	108.5
C10—C11—C12	120.60 (15)	C24—C23—C22	111.59 (12)
C10—C11—H11	119.7	C24—C23—H23A	109.3
C12—C11—H11	119.7	C22—C23—H23A	109.3
C11—C12—C7	119.26 (15)	C24—C23—H23B	109.3
C11—C12—C13	117.98 (14)	C22—C23—H23B	109.3
C7—C12—C13	122.71 (14)	H23A—C23—H23B	108.0
C12—C13—H13A	109.5	C25—C24—C23	111.85 (13)
C12—C13—H13B	109.5	C25—C24—H24A	109.2
H13A—C13—H13B	109.5	C23—C24—H24A	109.2

C12—C13—H13C	109.5	C25—C24—H24B	109.2
H13A—C13—H13C	109.5	C23—C24—H24B	109.2
H13B—C13—H13C	109.5	H24A—C24—H24B	107.9
N—C14—H14A	109.5	C26—C25—C24	111.22 (13)
N—C14—H14B	109.5	C26—C25—H25A	109.4
H14A—C14—H14B	109.5	C24—C25—H25A	109.4
N—C14—H14C	109.5	C26—C25—H25B	109.4
H14A—C14—H14C	109.5	C24—C25—H25B	109.4
H14B—C14—H14C	109.5	H25A—C25—H25B	108.0
N—C15—H15A	109.5	C25—C26—C27	111.14 (13)
N—C15—H15B	109.5	C25—C26—H26A	109.4
H15A—C15—H15B	109.5	C27—C26—H26A	109.4
N—C15—H15C	109.5	C25—C26—H26B	109.4
H15A—C15—H15C	109.5	C27—C26—H26B	109.4
H15B—C15—H15C	109.5	H26A—C26—H26B	108.0
C21—C16—C17	110.87 (12)	C26—C27—C22	111.95 (13)
C21—C16—P	112.02 (9)	C26—C27—H27A	109.2
C17—C16—P	109.26 (9)	C22—C27—H27A	109.2
C21—C16—H16	108.2	C26—C27—H27B	109.2
C17—C16—H16	108.2	C22—C27—H27B	109.2
P—C16—H16	108.2	H27A—C27—H27B	107.9
C16—P—C1—C2	55.56 (12)	C10—C11—C12—C7	−0.2 (2)
C22—P—C1—C2	−47.90 (12)	C10—C11—C12—C13	−177.65 (15)
C16—P—C1—C6	−124.70 (11)	C8—C7—C12—C11	0.4 (2)
C22—P—C1—C6	131.84 (11)	C6—C7—C12—C11	−178.87 (13)
C6—C1—C2—C3	−1.6 (2)	C8—C7—C12—C13	177.77 (13)
P—C1—C2—C3	178.19 (11)	C6—C7—C12—C13	−1.5 ( )

C1—C2—C3—C4	0.5 (2)	C1—P—C16—C21	−166.41 (10)
C2—C3—C4—C5	0.8 (2)	C22—P—C16—C21	−62.00 (11)
C3—C4—C5—C6	−1.0 (2)	C1—P—C16—C17	70.33 (11)
C4—C5—C6—C1	−0.1 (2)	C22—P—C16—C17	174.75 (10)
C4—C5—C6—C7	177.37 (14)	C21—C16—C17—C18	51.83 (18)
C2—C1—C6—C5	1.3 (2)	P—C16—C17—C18	175.77 (11)
P—C1—C6—C5	−178.40 (11)	C16—C17—C18—C19	−55.29 (18)
C2—C1—C6—C7	−176.05 (12)	C17—C18—C19—C20	57.22 (18)
P—C1—C6—C7	4.20 (18)	C18—C19—C20—C21	−57.05 (17)
C5—C6—C7—C8	110.05 (15)	C17—C16—C21—C20	−51.50 (17)
C1—C6—C7—C8	−72.54 (18)	P—C16—C21—C20	−173.84 (11)
C5—C6—C7—C12	−70.70 (18)	C19—C20—C21—C16	54.89 (17)
C1—C6—C7—C12	106.71 (16)	C1—P—C22—C23	−39.39 (11)
C12—C7—C8—C9	−0.2 (2)	C16—P—C22—C23	−141.20 (10)
C6—C7—C8—C9	179.02 (13)	C1—P—C22—C27	−160.61 (10)
C12—C7—C8—N	−178.82 (13)	C16—P—C22—C27	97.57 (10)
C6—C7—C8—N	0.4 (2)	C27—C22—C23—C24	−56.21 (16)
C14—N—C8—C9	107.75 (16)	P—C22—C23—C24	−177.08 (10)
C15—N—C8—C9	−21.4 (2)	C22—C23—C24—C25	56.14 (18)
C14—N—C8—C7	−73.69 (17)	C23—C24—C25—C26	−54.46 (19)
C15—N—C8—C7	157.15 (14)	C24—C25—C26—C27	54.59 (19)
C7—C8—C9—C10	−0.2 (2)	C25—C26—C27—C22	−56.95 (18)
N—C8—C9—C10	178.38 (14)	C23—C22—C27—C26	56.96 (16)
C8—C9—C10—C11	0.4 (3)	P—C22—C27—C26	−178.02 (11)
C9—C10—C11—C12	−0.3 (3)		

All e.s.d.'s (except the e.s.d. in the dihedral angle between two l.s. planes) are estimated using the full covariance matrix. The cell e.s.d.'s are taken into account individually in the estimation of e.s.d.'s in distances, angles and torsion angles; correlations between e.s.d.'s in

cell parameters are only used when they are defined by crystal symmetry. An approximate (isotropic) treatment of cell e.s.d.'s is used for estimating e.s.d.'s involving l.s. planes.

Computing details

Data collection: Collect (Nonius B.V., 1998); cell refinement: *DENZO* (Nonius B.V., 1998); data reduction: *DENZO* (Nonius B.V., 1998); program(s) used to solve structure: *SHELXS97* (Sheldrick, 1997); program(s) used to refine structure: *SHELXL97* (Sheldrick, 1997); molecular graphics: *PLATON* 98 (Spek, 1998); software used to prepare material for publication: *SHELXL97* (Sheldrick, 1997).

References

Burla, M. C., Camalli, M., Cascarano, G., Giacovazzo, C., Polidori, G., Spagna, R. & &Viterbo, D. (1989). *SIR*. J. Appl. Cryst. 22, 389-393.

A.L. Spek, *PLATON* 98, Utrecht University, The Netherland, 1998.

KappaCCD Operation Manual (1997). Nonius B.V., Delft, The Nederlands.

Otwinowski, Z. & Minor, W. (1997). *Methods in Enzymology*, **276**, 307–326.

Sheldrick, G. M. (1997). *SHELXL97*. Program for the refinement of crystal structures. University of Gottingen. Germany.
