# Supplementary material for: Bromine–lithium exchange: An efficient tool in the modular construction of biaryl ligands
Source: Beilstein J Org Chem. 2011 Sep 14;7:1278–87. doi: 10.3762/bjoc.7.148 (PMC3182437; doi:10.3762/bjoc.7.148)
Supplement: File 3 — Crystal structure data for 3b. [file Beilstein_J_Org_Chem-07-1278-s003.pdf]

Supporting Information

for

Bromine–lithium exchange: An efficient tool in the modular construction of biaryl ligands

Laurence Bonnafoux, Frédéric R. Leroux* and Françoise Colobert*

Address: Laboratoire de stéréochimie, UMR 7509, CNRS-Université de Strasbourg, ECPM, 25 rue Becquerel, F-67087 Strasbourg Cedex 02, France

Email: Laurence Bonnafoux - laurence.bonnafoux@gmail.com; Frédéric R. Leroux* - frederic.leroux@unistra.fr; Françoise Colobert* - francoise.colobert@unistra.fr

* Corresponding author

Crystal structure data for 3b.
CCDC 827186

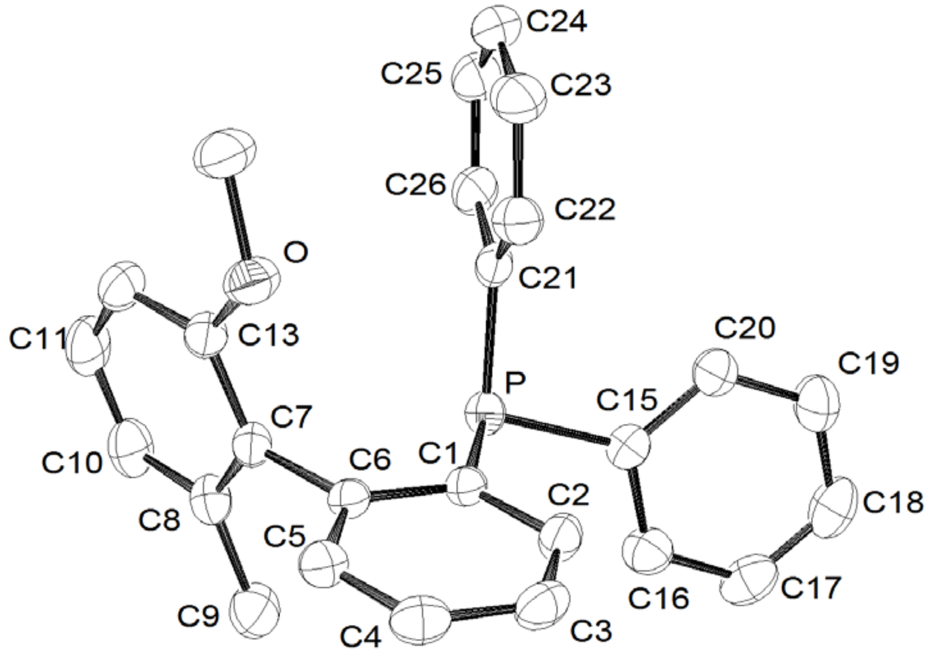

Crystal data

$C_{26}H_{23}OP$	
M_r = 382.41	D_x = 1.222 Mg m^{-3}
Monoclinic, *C*2/*c*	Melting point: ? K
Hall symbol: -C 2yc	Mo *K*α radiation, λ = 0.71073 Å
a = 17.0430 (3) Å	Cell parameters from 5029 reflections
b = 8.5060 (1) Å	θ = 1.0–29.1°
c = 29.1550 (5) Å	μ = 0.14 mm^{-1}
β = 100.2780 (7)°	*T* = 173 K
V = 4158.71 (11) Å^3	Prism, colorless
Z = 8	0.18 × 0.16 × 0.16 mm
F(000) = 1616	

Data collection

KappaCCD	4210 reflections with *I* > 2σ(*I*)

diffractometer	
Radiation source: fine-focus sealed tube	R_{int} = 0.021
graphite	θ_{max} = 29.1°, θ_{min} = 1.4°
Detector resolution: ? pixels mm^{-1}	$h = -22 \rightarrow 23$
π scans	$k = -11 \rightarrow 10$
9286 measured reflections	$l = -39 \rightarrow 39$
5552 independent reflections	

Refinement

Refinement on F^2	Secondary atom site location: difference Fourier map
Least-squares matrix: full	Hydrogen site location: inferred from neighbouring sites
$R[F^2 > 2\sigma(F^2)] = 0.043$	H-atom parameters constrained
$wR(F^2) = 0.116$	$w = 1/[\sigma^2(F_o^2) + (0.0465P)^2 + 2.2555P]$ where $P = (F_o^2 + 2F_c^2)/3$
$S = 1.06$	$(\Delta/\sigma)_{max} = 0.002$
5552 reflections	$\Delta\rho_{max}$ = 0.32 e Å^{-3}
253 parameters	$\Delta\rho_{min}$ = −0.34 e Å^{-3}
0 restraints	Extinction correction: none
Primary atom site location: structure-invariant direct methods	

Refinement of F^2 against ALL reflections. The weighted *R*-factor *wR* and goodness of fit S are based on F^2, conventional *R*-factors *R* are based on F, with F set to zero for negative F^2. The threshold expression of F^2 > 2sigma(F^2) is used only for calculating *R*-factors(gt), etc. and is not relevant to the choice of reflections for refinement. *R*-factors based on F^2 are statistically about twice as large as those based on F, and *R*– factors based on ALL data will be even larger.

Fractional atomic coordinates and isotropic or equivalent isotropic displacement parameters (Å^2)

	x	*y*	*Z*	$U_{iso}*/U_{eq}$

P	0.99375 (2)	0.37518 (4)	0.660704 (12)	0.02759 (10)
O	0.91170 (6)	0.58359 (13)	0.53409 (4)	0.0367 (2)
C1	1.05021 (7)	0.52952 (16)	0.63671 (4)	0.0253 (3)
C2	1.13309 (8)	0.52570 (18)	0.63970 (5)	0.0304 (3)
H2	1.1624	0.4379	0.6537	0.036*
C3	1.17304 (8)	0.64861 (19)	0.62245 (5)	0.0339 (3)
H3	1.2293	0.6442	0.6247	0.041*
C4	1.13126 (9)	0.77691 (18)	0.60212 (5)	0.0343 (3)
H4	1.1587	0.8611	0.5905	0.041*
C5	1.04904 (8)	0.78283 (17)	0.59865 (5)	0.0309 (3)
H5	1.0204	0.8712	0.5845	0.037*
C6	1.00802 (7)	0.66072 (16)	0.61572 (4)	0.0251 (3)
C7	0.91897 (8)	0.66908 (16)	0.61105 (5)	0.0275 (3)
C8	0.88313 (8)	0.71815 (18)	0.64828 (5)	0.0335 (3)
C9	0.93279 (10)	0.7681 (2)	0.69371 (6)	0.0449 (4)
H9A	0.8978	0.8002	0.7153	0.067*
H9B	0.9668	0.8567	0.6883	0.067*
H9C	0.9662	0.6801	0.7072	0.067*
C10	0.80017 (9)	0.7196 (2)	0.64262 (6)	0.0425 (4)
H10	0.7752	0.7511	0.6678	0.051*
C11	0.75401 (9)	0.6757 (2)	0.60086 (7)	0.0453 (4)
H11	0.6976	0.6771	0.5977	0.054*
C12	0.78843 (9)	0.62963 (19)	0.56353 (6)	0.0394 (4)
H12	0.7561	0.6009	0.5348	0.047*
C13	0.87118 (8)	0.62601 (17)	0.56871 (5)	0.0299 (3)
C14	0.86768 (10)	0.5097 (2)	0.49376 (6)	0.0454 (4)
H14A	0.8354	0.4244	0.5033	0.068*

H14B	0.9046	0.4668	0.4748	0.068*
H14C	0.8327	0.5871	0.4755	0.068*
C15	1.07408 (8)	0.25796 (17)	0.69458 (5)	0.0292 (3)
C16	1.11135 (9)	0.31910 (19)	0.73739 (5)	0.0383 (3)
H16	1.0954	0.4186	0.7473	0.046*
C17	1.17125 (10)	0.2364 (2)	0.76551 (5)	0.0446 (4)
H17	1.1969	0.2805	0.7942	0.053*
C18	1.19395 (10)	0.0900 (2)	0.75207 (6)	0.0473 (4)
H18	1.2354	0.0337	0.7713	0.057*
C19	1.15601 (10)	0.0260 (2)	0.71047 (6)	0.0480 (4)
H19	1.1704	−0.0759	0.7015	0.058*
C20	1.09700 (9)	0.10969 (19)	0.68179 (5)	0.0373 (3)
H20	1.0718	0.0653	0.6530	0.045*
C21	0.96099 (8)	0.25191 (16)	0.60933 (5)	0.0277 (3)
C22	0.99460 (9)	0.25641 (18)	0.56920 (5)	0.0338 (3)
H22	1.0389	0.3231	0.5681	0.041*
C23	0.96425 (10)	0.1648 (2)	0.53074 (5)	0.0410 (4)
H23	0.9873	0.1702	0.5034	0.049*
C24	0.90043 (10)	0.0655 (2)	0.53218 (6)	0.0446 (4)
H24	0.8794	0.0032	0.5058	0.054*
C25	0.86725 (9)	0.0570 (2)	0.57200 (7)	0.0448 (4)
H25	0.8241	−0.0125	0.5732	0.054*
C26	0.89691 (8)	0.14995 (19)	0.61018 (6)	0.0369 (3)
H26	0.8734	0.1443	0.6373	0.044*

Atomic displacement parameters (Å^2)

	U^{11}	U^{22}	U^{33}	U^{12}	U^{13}	U^{23}

P	0.02559 (19)	0.02855 (19)	0.02899 (19)	0.00195 (14)	0.00585 (13)	0.00076 (14)
O	0.0352 (5)	0.0418 (6)	0.0311 (5)	−0.0025 (5)	0.0005 (4)	−0.0048 (5)
C1	0.0237 (6)	0.0270 (7)	0.0243 (6)	0.0009 (5)	0.0024 (5)	−0.0035 (5)
C2	0.0235 (6)	0.0356 (8)	0.0311 (7)	0.0038 (6)	0.0021 (5)	−0.0022 (6)
C3	0.0225 (7)	0.0448 (9)	0.0341 (8)	−0.0048 (6)	0.0041 (5)	−0.0065 (6)
C4	0.0334 (7)	0.0356 (8)	0.0345 (8)	−0.0097 (6)	0.0075 (6)	−0.0030 (6)
C5	0.0321 (7)	0.0287 (7)	0.0303 (7)	0.0000 (6)	0.0016 (5)	−0.0010 (6)
C6	0.0239 (6)	0.0270 (7)	0.0236 (6)	0.0007 (5)	0.0015 (5)	−0.0042 (5)
C7	0.0240 (6)	0.0252 (7)	0.0321 (7)	0.0032 (5)	0.0020 (5)	0.0016 (5)
C8	0.0316 (7)	0.0318 (8)	0.0373 (8)	0.0070 (6)	0.0069 (6)	0.0009 (6)
C9	0.0441 (9)	0.0514 (10)	0.0392 (9)	0.0113 (8)	0.0073 (7)	−0.0075 (8)
C10	0.0341 (8)	0.0428 (9)	0.0532 (10)	0.0102 (7)	0.0150 (7)	0.0022 (8)
C11	0.0233 (7)	0.0427 (9)	0.0695 (12)	0.0039 (6)	0.0075 (7)	0.0056 (8)
C12	0.0272 (7)	0.0367 (8)	0.0499 (10)	−0.0015 (6)	−0.0054 (6)	0.0009 (7)
C13	0.0270 (7)	0.0263 (7)	0.0346 (7)	0.0005 (5)	0.0010 (5)	0.0015 (6)
C14	0.0519 (10)	0.0446 (10)	0.0339 (8)	−0.0043 (8)	−0.0079 (7)	−0.0027 (7)
C15	0.0287 (7)	0.0323 (7)	0.0267 (7)	−0.0006 (6)	0.0053 (5)	0.0031 (6)
C16	0.0445 (9)	0.0385 (8)	0.0308 (8)	−0.0002 (7)	0.0038 (6)	0.0006 (7)
C17	0.0448 (9)	0.0551 (11)	0.0302 (8)	−0.0070 (8)	−0.0033 (6)	0.0079 (7)
C18	0.0400 (9)	0.0566 (11)	0.0422 (9)	0.0066 (8)	−0.0009 (7)	0.0187 (8)
C19	0.0499 (10)	0.0414 (10)	0.0494 (10)	0.0142 (8)	−0.0003 (8)	0.0048 (8)
C20	0.0394 (8)	0.0357 (8)	0.0341 (8)	0.0057 (6)	−0.0008 (6)	0.0002 (6)
C21	0.0236 (6)	0.0266 (7)	0.0313 (7)	0.0034 (5)	0.0004 (5)	0.0031 (5)
C22	0.0374 (8)	0.0315 (8)	0.0319 (7)	−0.0015 (6)	0.0048 (6)	0.0012 (6)
C23	0.0522 (10)	0.0366 (8)	0.0320 (8)	0.0030 (7)	0.0015 (7)	−0.0012 (7)
C24	0.0435 (9)	0.0377 (9)	0.0455 (9)	0.0042 (7)	−0.0116 (7)	−0.0096 (7)

C25	0.0291 (8)	0.0383 (9)	0.0635 (11)	−0.0042 (7)	−0.0017 (7)	−0.0062 (8)
C26	0.0251 (7)	0.0389 (9)	0.0463 (9)	−0.0014 (6)	0.0056 (6)	−0.0017 (7)

Geometric parameters (Å, °)

P—C21	1.8321 (14)	C12—C13	1.392 (2)
P—C15	1.8335 (14)	C12—H12	0.9500
P—C1	1.8383 (14)	C14—H14A	0.9800
O—C13	1.3692 (17)	C14—H14B	0.9800
O—C14	1.4232 (18)	C14—H14C	0.9800
C1—C2	1.3999 (18)	C15—C20	1.391 (2)
C1—C6	1.4071 (18)	C15—C16	1.396 (2)
C2—C3	1.390 (2)	C16—C17	1.382 (2)
C2—H2	0.9500	C16—H16	0.9500
C3—C4	1.378 (2)	C17—C18	1.382 (3)
C3—H3	0.9500	C17—H17	0.9500
C4—C5	1.388 (2)	C18—C19	1.380 (3)
C4—H4	0.9500	C18—H18	0.9500
C5—C6	1.3925 (19)	C19—C20	1.385 (2)
C5—H5	0.9500	C19—H19	0.9500
C6—C7	1.5011 (18)	C20—H20	0.9500
C7—C8	1.4008 (19)	C21—C22	1.392 (2)
C7—C13	1.4010 (19)	C21—C26	1.398 (2)
C8—C10	1.394 (2)	C22—C23	1.388 (2)
C8—C9	1.500 (2)	C22—H22	0.9500
C9—H9A	0.9800	C23—C24	1.383 (2)
C9—H9B	0.9800	C23—H23	0.9500
C9—H9C	0.9800	C24—C25	1.381 (3)

C10—C11	1.378 (2)	C24—H24	0.9500
C10—H10	0.9500	C25—C26	1.385 (2)
C11—C12	1.382 (2)	C25—H25	0.9500
C11—H11	0.9500	C26—H26	0.9500

C21—P—C15	102.62 (6)	O—C13—C7	115.35 (12)
C21—P—C1	101.62 (6)	C12—C13—C7	120.56 (14)
C15—P—C1	101.66 (6)	O—C14—H14A	109.5
C13—O—C14	117.67 (12)	O—C14—H14B	109.5
C2—C1—C6	118.50 (12)	H14A—C14—H14B	109.5
C2—C1—P	123.41 (11)	O—C14—H14C	109.5
C6—C1—P	118.06 (9)	H14A—C14—H14C	109.5
C3—C2—C1	120.93 (13)	H14B—C14—H14C	109.5
C3—C2—H2	119.5	C20—C15—C16	118.21 (13)
C1—C2—H2	119.5	C20—C15—P	124.56 (11)
C4—C3—C2	120.16 (13)	C16—C15—P	117.12 (11)
C4—C3—H3	119.9	C17—C16—C15	120.71 (15)
C2—C3—H3	119.9	C17—C16—H16	119.6
C3—C4—C5	119.85 (13)	C15—C16—H16	119.6
C3—C4—H4	120.1	C18—C17—C16	120.35 (15)
C5—C4—H4	120.1	C18—C17—H17	119.8
C4—C5—C6	120.77 (13)	C16—C17—H17	119.8
C4—C5—H5	119.6	C19—C18—C17	119.58 (15)
C6—C5—H5	119.6	C19—C18—H18	120.2
C5—C6—C1	119.79 (12)	C17—C18—H18	120.2
C5—C6—C7	119.75 (12)	C18—C19—C20	120.23 (16)
C1—C6—C7	120.46 (12)	C18—C19—H19	119.9
C8—C7—C13	119.69 (13)	C20—C19—H19	119.9

C8—C7—C6	121.17 (12)	C19—C20—C15	120.87 (15)
C13—C7—C6	119.14 (12)	C19—C20—H20	119.6
C10—C8—C7	118.96 (14)	C15—C20—H20	119.6
C10—C8—C9	120.16 (14)	C22—C21—C26	118.15 (14)
C7—C8—C9	120.88 (13)	C22—C21—P	124.09 (11)
C8—C9—H9A	109.5	C26—C21—P	117.74 (11)
C8—C9—H9B	109.5	C23—C22—C21	120.92 (14)
H9A—C9—H9B	109.5	C23—C22—H22	119.5
C8—C9—H9C	109.5	C21—C22—H22	119.5
H9A—C9—H9C	109.5	C24—C23—C22	120.00 (15)
H9B—C9—H9C	109.5	C24—C23—H23	120.0
C11—C10—C8	120.62 (15)	C22—C23—H23	120.0
C11—C10—H10	119.7	C25—C24—C23	119.96 (15)
C8—C10—H10	119.7	C25—C24—H24	120.0
C10—C11—C12	121.13 (14)	C23—C24—H24	120.0
C10—C11—H11	119.4	C24—C25—C26	120.03 (15)
C12—C11—H11	119.4	C24—C25—H25	120.0
C11—C12—C13	119.02 (14)	C26—C25—H25	120.0
C11—C12—H12	120.5	C25—C26—C21	120.92 (15)
C13—C12—H12	120.5	C25—C26—H26	119.5
O—C13—C12	124.10 (13)	C21—C26—H26	119.5
C21—P—C1—C2	−95.87 (12)	C11—C12—C13—C7	−0.3 (2)
C15—P—C1—C2	9.81 (13)	C8—C7—C13—O	178.42 (13)
C21—P—C1—C6	86.42 (11)	C6—C7—C13—O	−1.95 (19)
C15—P—C1—C6	−16□.90 (10)	C8—C7—C13—C12	−0.8 (2)
C6—C1—C2—C3	0.1 (2)	C6—C7—C13—C12	178.79 (13)
P—C1—C2—C3	−177.57 (11)	C21—P—C15—C20	−2.41 (14)

C1—C2—C3—C4	0.1 (2)	C1—P—C15—C20	−107.30 (13)
C2—C3—C4—C5	−0.4 (2)	C21—P—C15—C16	−178.61 (11)
C3—C4—C5—C6	0.3 (2)	C1—P—C15—C16	76.50 (12)
C4—C5—C6—C1	−0.1 (2)	C20—C15—C16—C17	2.1 (2)
C4—C5—C6—C7	−179.31 (13)	P—C15—C16—C17	178.56 (12)
C2—C1—C6—C5	−0.16 (19)	C15—C16—C17—C18	−1.4 (2)
P—C1—C6—C5	177.66 (10)	C16—C17—C18—C19	−0.5 (3)
C2—C1—C6—C7	179.07 (12)	C17—C18—C19—C20	1.7 (3)
P—C1—C6—C7	−3.11 (16)	C18—C19—C20—C15	−1.0 (3)
C5—C6—C7—C8	−98.82 (16)	C16—C15—C20—C19	−0.9 (2)
C1—C6—C7—C8	81.94 (17)	P—C15—C20—C19	−177.06 (13)
C5—C6—C7—C13	81.55 (17)	C15—P—C21—C22	−87.56 (13)
C1—C6—C7—C13	−97.68 (16)	C1—P—C21—C22	17.36 (13)
C13—C7—C8—C10	1.4 (2)	C15—P—C21—C26	94.11 (12)
C6—C7—C8—C10	−178.17 (14)	C1—P—C21—C26	−160.97 (11)
C13—C7—C8—C9	−178.74 (14)	C26—C21—C22—C23	1.4 (2)
C6—C7—C8—C9	1.6 (2)	P—C21—C22—C23	−176.95 (12)
C7—C8—C10—C11	−1.0 (2)	C21—C22—C23—C24	−0.9 (□)
C9—C8—C10—C11	179.22 (16)	C22—C23—C24—C25	−0.4 (3)
C8—C10—C11—C12	−0.2 (3)	C23—C24—C25—C26	1.2 (3)
C10—C11—C12—C13	0.8 (3)	C24—C25—C26—C21	−0.7 (2)
C14—O—C13—C12	−12.9 (2)	C22—C21—C26—C25	−0.6 (2)
C14—O—C13—C7	167.88 (13)	P—C21—C26—C25	177.87 (12)
C11—C12—C13—O	−179.47 (15)		

All e.s.d.'s (except the e.s.d. in the dihedral angle between two l.s. planes) are estimated using the full covariance matrix. The cell e.s.d.'s are taken into account individually in the estimation of e.s.d.'s in distances, angles and torsion angles; correlations between e.s.d.'s in cell parameters are only used when they are defined by crystal symmetry. An approximate (isotropic) treatment of cell e.s.d.'s is used for estimating e.s.d.'s involving l.s. planes.

Computing details

Data collection: Collect (Nonius B.V., 1998); cell refinement: *DENZO* (Nonius B.V., 1998); data reduction: *DENZO* (Nonius B.V., 1998); program(s) used to solve structure: *SHELXS97* (Sheldrick, 1997); program(s) used to refine structure: *SHELXL97* (Sheldrick, 1997); molecular graphics: *PLATON* 98 (Spek, 1998); software used to prepare material for publication: *SHELXL97* (Sheldrick, 1997).

References

Burla, M. C., Camalli, M., Cascarano, G., Giacovazzo, C., Polidori, G., Spagna, R. & &Viterbo, D. (1989). *SIR*. J. Appl. Cryst. 22, 389-393.

A.L. Spek, *PLATON* 98, Utrecht University, The Netherland, 1998.

KappaCCD Operation Manual (1997). Nonius B.V., Delft, The Nederlands.

Otwinowski, Z. & Minor, W. (1997). *Methods in Enzymology*, **276**, 307–326.

Sheldrick, G. M. (1997). *SHELXL97*. Program for the refinement of crystal structures. University of Gottingen. Germany.
