# Supplementary material for: Bromine–lithium exchange: An efficient tool in the modular construction of biaryl ligands
Source: Beilstein J Org Chem. 2011 Sep 14;7:1278–87. doi: 10.3762/bjoc.7.148 (PMC3182437; doi:10.3762/bjoc.7.148)
Supplement: File 4 — Crystal structure data for 3d. [file Beilstein_J_Org_Chem-07-1278-s004.pdf]

Supporting Information

for

Bromine–lithium exchange: An efficient tool in the modular construction of biaryl ligands

Laurence Bonnafoux, Frédéric R. Leroux* and Françoise Colobert*

Address: Laboratoire de stéréochimie, UMR 7509, CNRS-Université de Strasbourg, ECPM, 25 rue Becquerel, F-67087 Strasbourg Cedex 02, France

Email: Laurence Bonnafoux - laurence.bonnafoux@gmail.com; Frédéric R. Leroux* - frederic.leroux@unistra.fr; Françoise Colobert* - francoise.colobert@unistra.fr

* Corresponding author

Crystal structure data for 3d.
CCDC 827187

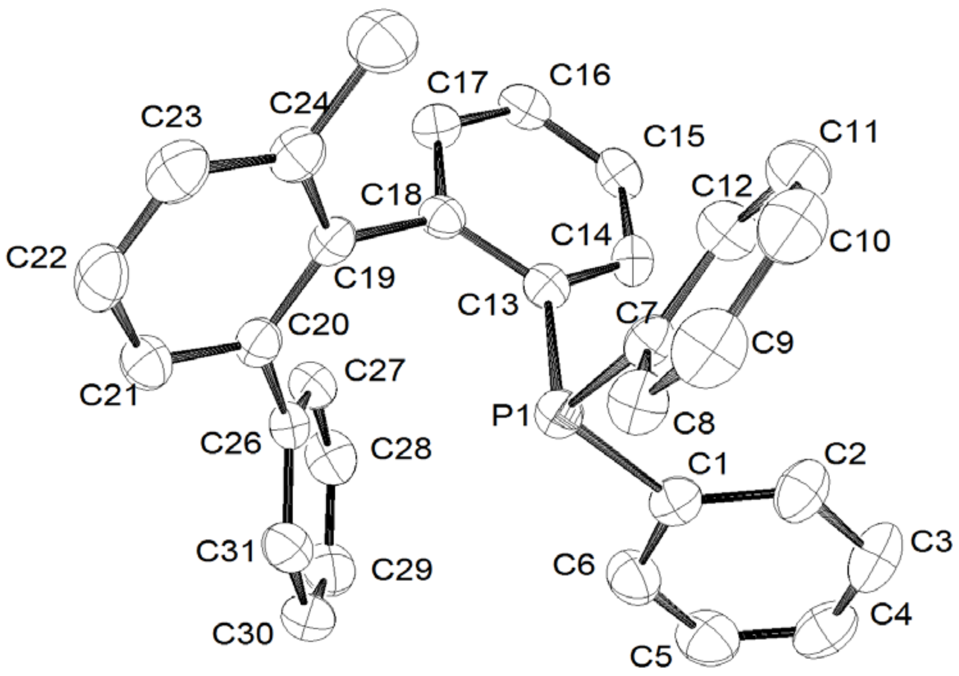

Crystal data

$C_{31}H_{25}P$	
M_r = 428.48	D_x = 1.200 Mg m^{-3}
Monoclinic, $P2_1/c$	Melting point: ? K
Hall symbol: -P 2ybc	Mo *K*α radiation, λ = 0.71073 Å
a = 14.1270 (6) Å	Cell parameters from 8522 reflections
b = 11.3350 (4) Å	θ = 1.0–27.5°
c = 16.5810 (5) Å	μ = 0.13 mm^{-1}
β = 116.748 (2)°	*T* = 173 K
V = 2371.00 (15) Å^3	Block, colorless
Z = 4	0.40 × 0.35 × 0.30 mm
F(000) = 904	

Data collection

CCD area detector diffractometer	3702 reflections with $I > 2\sigma(I)$
Radiation source: fine-focus sealed tube	R_{int} = 0.036

graphite	θ_{max} = 27.5°, θ_{min} = 1.6°
Detector resolution: ? pixels mm^{-1}	$h = -18 \rightarrow 18$
phi and ω scans	$k = -14 \rightarrow 13$
9678 measured reflections	$l = -21 \rightarrow 21$
5442 independent reflections	

Refinement

Refinement on F^2	Secondary atom site location: difference Fourier map
Least-squares matrix: full	Hydrogen site location: inferred from neighbouring sites
$R[F^2 > 2\sigma(F^2)]$ = 0.046	H-atom parameters constrained
$wR(F^2)$ = 0.120	$w = 1/[\sigma^2(F_o^2) + (0.0604P)^2 + 0.0691P]$ where $P = (F_o^2 + 2F_c^2)/3$
S = 1.03	$(\Delta/\sigma)_{max}$ <0.001
5442 reflections	$\Delta\rho_{max}$ = 0.19 e Å^{-3}
290 parameters	$\Delta\rho_{min}$ = −0.31 e Å^{-3}
0 restraints	Extinction correction: none
Primary atom site location: structure-invariant direct methods	

Refinement of F^2 against ALL reflections. The weighted *R*-factor *wR* and goodness of fit S are based on F^2, conventional *R*-factors *R* are based on F, with F set to zero for negative F^2. The threshold expression of F^2 > 2sigma(F^2) is used only for calculating *R*-factors(gt), etc. and is not relevant to the choice of reflections for refinement. *R*-factors based on F^2 are statistically about twice as large as those based on F, and *R*– factors based on ALL data will be even larger.

Fractional atomic coordinates and isotropic or equivalent isotropic displacement parameters (Å^2)

	x	*y*	*z*	$U_{iso}*/U_{eq}$
C1	0.31941 (12)	0.05649 (15)	0.71721 (10)	0.0314 (4)
C2	0.38978 (14)	0.11332 (16)	0.79591 (11)	0.0408 (4)

H2	0.3932	0.1970	0.7977	0.049*
C3	0.45525 (15)	0.04825 (18)	0.87204 (11)	0.0482 (5)
H3	0.5033	0.0877	0.9254	0.058*
C4	0.45039 (15)	−0.07319 (17)	0.87014 (12)	0.0475 (5)
H4	0.4944	−[illegible].1175	0.9223	0.057*
C5	0.38160 (14)	−0.12996 (17)	0.79255 (13)	0.0495 (5)
H5	0.3788	−0.2137	0.7908	0.059*
C6	0.31652 (13)	−0.06569 (16)	0.71719 (12)	0.0426 (5)
H6	0.2687	−0.1060	0.6642	0.051*
C7	0.23416 (12)	0.28440 (14)	0.64601 (10)	0.0287 (4)
C8	0.14840 (13)	0.32295 (16)	0.65905 (11)	0.0367 (4)
H8	0.0934	0.2690	0.6512	0.044*
C9	0.14195 (15)	0.43818 (17)	0.68317 (12)	0.0454 (5)
H9	0.0831	0.4626	0.6923	0.055*
C10	0.22068 (15)	0.51817 (17)	0.69409 (11)	0.0452 (5)
H10	0.2156	0.5978	0.7096	0.054*
C11	0.30641 (15)	0.48143 (16)	0.68227 (12)	0.0443 (5)
H11	0.3612	0.5358	0.6906	0.053*
C12	0.31352 (13)	0.36579 (15)	0.65836 (11)	0.0369 (4)
H12	0.3731	0.3417	0.6503	0.044*
C13	0.31834 (11)	0.13377 (13)	0.55586 (10)	0.0268 (3)
C14	0.42828 (12)	0.12213 (14)	0.60248 (10)	0.0299 (4)
H14	0.4605	0.1103	0.6660	0.036*
C15	0.49139 (12)	0.12729 (13)	0.55888 (11)	0.0314 (4)
H15	0.5660	0.1181	0.5922	0.038*
C16	0.44598 (12)	0.14580 (14)	0.46661 (11)	0.0323 (4)
H16	0.4892	0.1516	0.4364	0.039*

C17	0.33704 (12)	0.15577 (14)	0.41883 (11)	0.0311 (4)
H17	0.3059	0.1679	0.3554	0.037*
C18	0.27172 (12)	0.14847 (13)	0.46131 (10)	0.0263 (3)
C19	0.15423 (12)	0.15430 (14)	0.40443 (10)	0.0287 (4)
C20	0.09093 (12)	0.05268 (14)	0.38792 (10)	0.0294 (4)
C21	−0.01770 (13)	0.06120 (16)	0.33100 (11)	0.0365 (4)
H21	−0.0616	−0.0062	0.3211	0.044*
C22	−0.06206 (13)	0.16624 (17)	0.28897 (11)	0.0423 (5)
H22	−0.1356	0.1700	0.2485	0.051*
C23	−0.00004 (13)	0.26565 (17)	0.30551 (11)	0.0407 (4)
H23	−0□0314	0.3376	0.2763	0.049*
C24	0.10804 (12)	0.26238 (15)	0.36437 (10)	0.0343 (4)
C25	0.17240 (15)	0.37429 (16)	0.38470 (13)	0.0490 (5)
H25A	0.2120	0.3761	0.3491	0.074*
H25B	0.2221	0.3769	0.4492	0.074*
H25C	0.1249	0.4426	0.3691	0.074*
C26	0.13444 (11)	−0.06497 (14)	0.42756 (10)	0.0291 (4)
C27	0.21867 (12)	−0.11688 (15)	0.41837 (11)	0.0342 (4)
H27	0.2525	−0.0746	0.3891	0.041*
C28	0.25362 (14)	−0.22900 (16)	0.45135 (12)	0.0416 (4)
H28	0.3111	−0.2629	0.4445	0.050*
C29	0.20549 (14)	−0.29206 (16)	0.49414 (12)	0.0450 (5)
H29	0.2293	−0.3693	0.5163	0.054*
C30	0.12258 (14)	−0.24194 (17)	0.50441 (12)	0.0433 (4)
H30	0.0897	−0.2845	0.5344	0.052*
C31	0.08694 (13)	−0.12974 (15)	0.47125 (11)	0.0357 (4)
H31	0.0294	−0.0965	0.4784	0.043*

P1	0.23096 (3)	0.13002 (4)	0.61125 (3)	0.02832 (13)

Atomic displacement parameters (Å^2)

	U^{11}	U^{22}	U^{33}	U^{12}	U^{13}	U^{23}
C1	0.0301 (8)	0.0343 (10)	0.0331 (9)	0.0050 (7)	0.0170 (7)	0.0028 (7)
C2	0.0525 (11)	0.0366 (10)	0.0323 (9)	0.0111 (8)	0.0183 (8)	−0.0004 (8)
C3	0.0544 (12)	0.0547 (13)	0.0291 (9)	0.0165 (10)	0.0132 (8)	0.0006 (8)
C4	0.0521 (12)	0.0530 (13)	0.0404 (10)	0.0188 (10)	0.0234 (9)	0.0165 (9)
C5	0.0495 (11)	0.0361 (11)	0.0582 (13)	0.0048 (9)	0.0200 (10)	0.0137 (9)
C6	0.0378 (10)	0.0364 (11)	0.0452 (10)	−0.0019 (8)	0.0112 (8)	0.0050 (8)
C7	0.0283 (8)	0.0323 (9)	0.0238 (8)	0.0053 (7)	0.0104 (6)	0.0036 (7)
C8	0.0333 (9)	0.0403 (10)	0.0370 (9)	0.0049 (8)	0.0161 (7)	−0.0011 (8)
C9	0.0458 (11)	0.0469 (12)	0.0487 (11)	0.0153 (9)	0.0256 (9)	−0.0014 (9)
C10	0.0589 (12)	0.0336 (11)	0.0412 (10)	0.0112 (9)	0.0208 (9)	−0.0005 (8)
C11	0.0524 (11)	0.0346 (11)	0.0447 (10)	−0.0036 (9)	0.0209 (9)	−0.0013 (8)
C12	0.0330 (9)	0.0359 (10)	0.0438 (10)	0.0023 (8)	0.0192 (8)	−0.0014 (8)
C13	0.0267 (8)	0.0233 (8)	0.0308 (8)	0.0012 (6)	0.0132 (7)	−0.0008 (6)
C14	0.0273 (8)	0.0305 (9)	0.0278 (8)	0.0017 (7)	0.0087 (6)	−0.0038 (7)
C15	0.0227 (8)	0.0279 (9)	0.0424 (9)	0.0007 (7)	0.0136 (7)	−0.0057 (7)
C16	0.0336 (9)	0.0268 (9)	0.0435 (10)	−0.0027 (7)	0.0237 (8)	−0.0044 (7)

C17	0.0325 (9)	0.0300 (9)	0.0321 (8)	0.0010 (7)	0.0157 (7)	0.0018 (7)
C18	0.0266 (8)	0.0212 (8)	0.0309 (8)	0.0004 (6)	0.0127 (7)	0.0013 (6)
C19	0.0283 (8)	0.0343 (9)	0.0240 (8)	0.0040 (7)	0.0121 (7)	0.0029 (7)
C20	0.0269 (8)	0.0354 (9)	0.0260 (8)	0.0020 (7)	0.0119 (6)	0.0008 (7)
C21	0.0280 (8)	0.0433 (11)	0.0365 (9)	−0.0013 (8)	0.0131 (7)	0.0008 (8)
C22	0.0266 (9)	0.0574 (12)	0.0365 (10)	0.0093 (8)	0.0087 (7)	0.0054 (9)
C23	0.0379 (10)	0.0427 (11)	0.0403 (10)	0.0153 (8)	0.0165 (8)	0.0110 (8)
C24	0.0349 (9)	0.0365 (10)	0.0326 (9)	0.0067 (8)	0.0162 (7)	0.0040 (7)
C25	0.0498 (11)	0.0360 (11)	0.0576 (12)	0.0075 (9)	0.0209 (10)	0.0088 (9)
C26	0.0244 (8)	0.0320 (9)	0.0264 (8)	−0.0030 (7)	0.0075 (6)	−0.0019 (7)
C27	0.0312 (9)	0.0335 (10)	0.0379 (9)	−0.0016 (7)	0.0155 (7)	−0.0005 (8)
C28	0.0365 (9)	0.0356 (10)	0.0492 (10)	0.0054 (8)	0.0161 (8)	−0.0009 (8)
C29	0.0477 (11)	0.0299 (10)	0.0488 (11)	−0.0014 (8)	0.0140 (9)	0.0043 (8)
C30	0.0473 (11)	0.0381 (10)	0.0433 (10)	−0.0098 (9)	0.0194 (8)	0.0027 (8)
C31	0.0319 (9)	0.0370 (10)	0.0396 (9)	−0.0033 (8)	0.0172 (7)	−0.0025 (8)
P1	0.0259 (2)	0.0307 (2)	0.0287 (2)	0.00177 (17)	0.01255 (17)	0.00028 (17)

Geometric parameters (Å, °)

C1—C6	1.386 (2)	C16—C17	1.383 (2)
C1—C2	1.392 (2)	C16—H16	0.9500
C1—P1	1.8348 (16)	C17—C18	1.393 (2)

C2—C3	1.394 (2)	C17—H17	0.9500
C2—H2	0.9500	C18—C19	1.497 (2)
C3—C4	1.378 (3)	C19—C24	1.406 (2)
C3—H3	0.9500	C19—C20	1.407 (2)
C4—C5	1.374 (3)	C20—C21	1.397 (2)
C4—H4	0.9500	C20—C26	1.492 (2)
C5—C6	1.380 (2)	C21—C22	1.379 (2)
C5—H5	0.9500	C21—H21	0.9500
C6—H6	0.9500	C22—C23	1.377 (3)
C7—C8	1.393 (2)	C22—H22	0.9500
C7—C12	1.394 (2)	C23—C24	1.394 (2)
C7—P1	1.8365 (16)	C23—H23	0.9500
C8—C9	1.381 (2)	C24—C25	1.508 (2)
C8—H8	0.9500	C25—H25A	0.9800
C9—C10	1.382 (3)	C25—H25B	0.9800
C9—H9	0.9500	C25—H25C	0.9800
C10—C11	1.376 (3)	C26—C27	1.396 (2)
C10—H10	0.9500	C26—C31	1.397 (2)
C11—C12	1.386 (2)	C27—C28	1.384 (2)
C11—H11	0.9500	C27—H27	0.9500
C12—H12	0.9500	C28—C29	1.382 (3)
C13—C14	1.395 (2)	C28—H28	0.9500
C13—C18	1.411 (2)	C29—C30	1.380 (3)
C13—P1	1.8411 (16)	C29—H29	0.9500
C14—C15	1.379 (2)	C30—C31	1.387 (2)
C14—H14	0.9500	C30—H30	0.9500
C15—C16	1.383 (2)	C31—H31	0.9500

C15—H15	0.9500		
C6—C1—C2	118.12 (15)	C18—C17—H17	119.1
C6—C1—P1	116.52 (12)	C17—C18—C13	119.01 (14)
C2—C1—P1	125.35 (13)	C17—C18—C19	118.56 (13)
C1—C2—C3	120.46 (18)	C13—C18—C19	122.43 (14)
C1—C2—H2	119.8	C24—C19—C20	120.13 (13)
C3—C2—H2	119.8	C24—C19—C18	118.90 (14)
C4—C3—C2	120.14 (18)	C20—C19—C18	120.92 (13)
C4—C3—H3	119.9	C21—C20—C19	118.91 (14)
C2—C3—H3	119.9	C21—C20—C26	117.98 (14)
C5—C4—C3	119.75 (17)	C19—C20—C26	123.11 (13)
C5—C4—H4	120.1	C22—C21—C20	120.81 (16)
C3—C4—H4	120.1	C22—C21—H21	119.6
C4—C5—C6	120.19 (19)	C20—C21—H21	119.6
C4—C5—H5	119.9	C23—C22—C21	120.13 (15)
C6—C5—H5	119.9	C23—C22—H22	119.9
C5—C6—C1	121.33 (17)	C21—C22—H22	119.9
C5—C6—H6	119.3	C22—C23—C24	121.05 (16)
C1—C6—H6	119.3	C22—C23—H23	119.5
C8—C7—C12	117.88 (15)	C24—C23—H23	119.5
C8—C7—P1	116.56 (12)	C23—C24—C19	118.87 (16)
C12—C7—P1	125.54 (12)	C23—C24—C25	119.63 (15)
C9—C8—C7	121.13 (17)	C19—C24—C25	121.49 (14)
C9—C8—H8	119.4	C24—C25—H25A	109.5
C7—C8—H8	119.4	C24—C25—H25B	109.5
C8—C9—C10	120.28 (17)	H25A—C25—H25B	109.5
C8—C9—H9	119.9	C24—C25—H25C	109.5

C10—C9—H9	119.9	H25A—C25—H25C	109.5
C11—C10—C9	119.40 (17)	H25B—C25—H25C	109.5
C11—C10—H10	120.3	C27—C26—C31	117.85 (15)
C9—C10—H10	120.3	C27—C26—C20	122.09 (14)
C10—C11—C12	120.59 (18)	C31—C26—C20	119.96 (14)
C10—C11—H11	119.7	C28—C27—C26	120.93 (16)
C12—C11—H11	119.7	C28—C27—H27	119.5
C11—C12—C7	120.71 (16)	C26—C27—H27	119.5
C11—C12—H12	119.6	C29—C28—C27	120.48 (17)
C7—C12—H12	119.6	C29—C28—H28	119.8
C14—C13—C18	118.26 (14)	C27—C28—H28	119.8
C14—C13—P1	123.41 (12)	C30—C29—C28	119.44 (17)
C18—C13—P1	118.34 (11)	C30—C29—H29	120.3
C15—C14—C13	121.79 (14)	C28—C29—H29	120.3
C15—C14—H14	119.1	C29—C30—C31	120.37 (17)
C13—C14—H14	119.1	C29—C30—H30	119.8
C14—C15—C16	119.94 (14)	C31—C30—H30	119.8
C14—C15—H15	120.0	C30—C31—C26	120.93 (16)
C16—C15—H15	120.0	C30—C31—H31	119.5
C17—C16—C15	119.25 (15)	C26—C31—H31	119.5
C17—C16—H16	120.4	C1—P1—C7	102.85 (7)
C15—C16—H16	120.4	C1—P1—C13	99.65 (7)
C16—C17—C18	121.70 (15)	C7—P1—C13	101.96 (7)
C16—C17—H17	119.1		
C6—C1—C2—C3	0.1 (3)	C19—C20—C21—C22	2.2 (2)
P1—C1—C2—C3	−178.44 (14)	C26—C20—C21—C22	−177.05 (15)
C1—C2—C3—C4	−0.4 (3)	C20—C21—C22—C23	−2.5 (3)

C2—C3—C4—C5	0.7 (3)	C21—C22—C23—C24	0.2 (3)
C3—C4—C5—C6	−0.9 (3)	C22—C23—C24—C19	2.4 (3)
C4—C5—C6—C1	0.7 (3)	C22—C23—C24—C25	−176.78 (17)
C2—C1—C6—C5	−0.3 (3)	C20—C19—C24—C23	−2.6 (2)
P1—C1—C6—C5	178.39 (15)	C18—C19—C24—C23	174.82 (15)
C12—C7—C8—C9	0.2 (2)	C20—C19—C24—C25	176.54 (16)
P1—C7—C8—C9	−178.48 (13)	C18—C19—C24—C25	−6.0 (2)
C7—C8—C9—C10	0.6 (3)	C21—C20—C26—C27	125.78 (16)
C8—C9—C10—C11	−1.1 (3)	C19—C20—C26—C27	−53.5 (2)
C9—C10—C11—C12	0.9 (3)	C21—C20—C26—C31	−50.6 (2)
C10—C11—C12—C7	−0.1 (3)	C19—C20—C26—C31	130.13 (17)
C8—C7—C12—C11	−0.4 (2)	C31—C26—C27—C28	0.2 (2)
P1—C7—C12—C11	178.13 (13)	C20—C26—C27—C28	−176.29 (14)
C18—C13—C14—C15	1.5 (2)	C26—C27—C28—C29	0.1 (2)
P1—C13—C14—C15	−178.93 (12)	C27—C28—C29—C30	−0.5 (3)
C13—C14—C15—C16	0.8 (2)	C28—C29—C30—C31	0.7 (3)
C14—C15—C16—C17	−1.7 (2)	C29—C30—C31—C26	−0.5 (2)
C15—C16—C17—C18	0.5 (2)	C27—C26—C31—C30	0.0 (2)
C16—C17—C18—C13	1.8 (2)	C20—C26—C31—C30	176.58 (14)
C16—C17—C18—C19	−177.10 (14)	C6—C1—P1—C7	166.92 (13)
C14—C13—C18—C17	−2.7 (2)	C2—C1—P1—C7	−14.47 (17)
P1—C13—C18—C17	177.70 (11)	C6—C1—P1—C13	−88.34 (14)
C14—C13—C18—C19	176.15 (14)	C2—C1—P1—C13	90.26 (16)
P1—C13—C18—C19	−3.5 (2)	C8—C7—P1—C1	−100.54 (12)
C17—C18—C19—C24	−71.07 (19)	C12—C7—P1—C1	80.91 (15)
C13—C18—C19—C24	110.10 (17)	C8—C7—P1—C13	156.52 (12)
C17—C18—C19—C20	106.35 (18)	C12—C7—P1—C13	−22.0□ (15)

C13—C18—C19—C20	−72.5 (2)	C14—C13—P1—C1	−19.81 (14)
C24—C19—C20—C21	0.3 (2)	C18—C13—P1—C1	159.81 (12)
C18—C19—C20—C21	−177.04 (14)	C14—C13—P1—C7	85.65 (14)
C24—C19—C20—C26	179.61 (14)	C18—C13—P1—C7	−94.73 (12)
C18—C19—C20—C26	2.2 (2)		

All e.s.d.'s (except the e.s.d. in the dihedral angle between two l.s. planes) are estimated using the full covariance matrix. The cell e.s.d.'s are taken into account individually in the estimation of e.s.d.'s in distances, angles and torsion angles; correlations between e.s.d.'s in cell parameters are only used when they are defined by crystal symmetry. An approximate (isotropic) treatment of cell e.s.d.'s is used for estimating e.s.d.'s involving l.s. planes.

Computing details

Data collection: Collect (Nonius B.V., 1998); cell refinement: *DENZO* (Nonius B.V., 1998); data reduction: *DENZO* (Nonius B.V., 1998); program(s) used to solve structure: *SHELXS97* (Sheldrick, 1997); program(s) used to refine structure: *SHELXL97* (Sheldrick, 1997); molecular graphics: *PLATON* 98 (Spek, 1998); software used to prepare material for publication: *SHELXL97* (Sheldrick, 1997).
