# Supplementary material for: Bromine–lithium exchange: An efficient tool in the modular construction of biaryl ligands
Source: Beilstein J Org Chem. 2011 Sep 14;7:1278–87. doi: 10.3762/bjoc.7.148 (PMC3182437; doi:10.3762/bjoc.7.148)
Supplement: File 5 — Crystal structure data for 3f. [file Beilstein_J_Org_Chem-07-1278-s005.pdf]

Supporting Information

for

Bromine–lithium exchange: An efficient tool in the modular construction of biaryl ligands

Laurence Bonnafoux, Frédéric R. Leroux* and Françoise Colobert*

Address: Laboratoire de stéréochimie, UMR 7509, CNRS-Université de Strasbourg, ECPM, 25 rue Becquerel, F-67087 Strasbourg Cedex 02, France

Email: Laurence Bonnafoux - laurence.bonnafoux@gmail.com; Frédéric R. Leroux* - frederic.leroux@unistra.fr; Françoise Colobert* - francoise.colobert@unistra.fr

* Corresponding author

Crystal structure data for 3f.
CCDC 827189

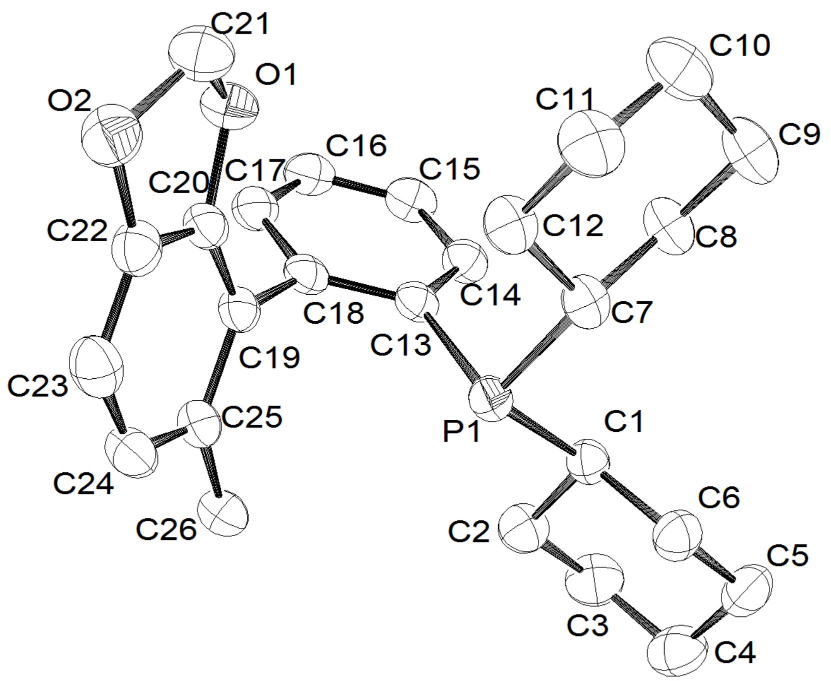

Crystal data

$C_{26}H_{33}O_2P$	
M_r = 408.49	D_x = 1.207 Mg m^{-3}
Monoclinic, *P*2$_1$/*c*	Melting point: ? K
Hall symbol: -P 2ybc	Mo *K*α radiation, λ = 0.71073 Å
a = 10.5090 (4) Å	Cell parameters from 10201 reflections
b = 15.8680 (9) Å	θ = 1.0–27.5°
c = 15.6060 (5) Å	μ = 0.14 mm^{-1}
β = 120.241 (2)°	*T* = 173 K
V = 2248.26 (17) Å^3	Block, colorless
Z = 4	0.35 × 0.30 × 0.25 mm
F(000) = 880	

Data collection

CCD area detector diffractometer	3633 reflections with $I > 2\sigma(I)$
Radiation source: fine-focus sealed tube	R_{int} = 0.028
graphite	θ_{max} = 27.5°, θ_{min} = 2.0°
Detector resolution: ? pixels mm^{-1}	$h = -13 \rightarrow 13$
phi and ω scans	$k = -20 \rightarrow 19$
8893 measured reflections	$l = -20 \rightarrow 20$
5132 independent reflections	

Refinement

Refinement on F^2	Secondary atom site location: difference Fourier map
Least-squares matrix: full	Hydrogen site location: inferred from neighbouring sites
$R[F^2 > 2\sigma(F^2)]$ = 0.044	H-atom parameters constrained
$wR(F^2)$ = 0.118	$w = 1/[\sigma^2(F_o^2) + (0.0558P)^2 + 0.1809P]$ where $P = (F_o^2 + 2F_c^2)/3$
S = 1.07	$(\Delta/\sigma)_{max}$ <0.001
5132 reflections	$\Delta\rho_{max}$ = 0.25 e Å^{-3}
263 parameters	$\Delta\rho_{min}$ = −0.29 e Å^{-3}
0 restraints	Extinction correction: none
Primary atom site location: structure-invariant direct methods	

Refinement of F^2 against ALL reflections. The weighted *R*-factor *wR* and goodness of fit S are based on F^2, conventional *R*-factors *R* are based on F, with F set to zero for negative F^2. The threshold expression of $F^2 > 2sigma(F^2)$ is used only for calculating *R*-factors(gt), etc. and is not relevant to the choice of reflections for refinement. *R*-factors based on F^2 are statistically about twice as large as those based on F, and *R*– factors based on ALL data will be even larger.

Fractional atomic coordinates and isotropic or equivalent isotropic displacement parameters (Å^2)

	x	*y*	*z*	U_{iso}*/U_{eq}
C1	0.33029 (17)	0.10674 (10)	0.03152 (11)	0.0307 (4)
H1	0.3836	0.0976	−0.0057	0.037*
C2	0.16784 (19)	0.08241 (11)	−0.03540 (13)	0.0401 (4)
H2A	0.1128	0.0959	−0.0010	0.048*
H2B	0.1251	0.1162	−0.0971	0.048*
C3	0.1498 (2)	−0.01127 (13)	−0.06183 (14)	0.0504 (5)
H3A	0.0437	−0.0251	−0.1021	0.060*
H3B	0.1962	−0.0238	−0.1021	0.060*
C4	0.2203 (2)	−0.06511 (13)	0.03106 (14)	0.0523 (5)
H4A	0.2129	−0.1252	0.0122	0.063*
H4B	0.1664	−0.0573	0.0674	0.063*
C5	0.3815 (2)	−0.04201 (12)	0.09858 (13)	0.0460 (5)
H5A	0.4225	−0.0757	0.1603	0.055*
H5B	0.4376	−0.0562	0.0651	0.055*
C6	0.40039 (19)	0.05130 (11)	0.12465 (12)	0.0386 (4)
H6A	0.5067	0.0646	0.1650	0.046*
H6B	0.3543	0.0641	0.1650	0.046*
C7	0.54442 (17)	0.24031 (11)	0.13682 (11)	0.0330 (4)
H7	0.5820	0.2105	0.2016	0.040*
C8	0.63789 (18)	0.20828 (12)	0.09346 (12)	0.0396 (4)
H8A	0.6234	0.1468	0.0819	0.048*
H8B	0.6053	0.2360	0.0288	0.048*
C9	0.80113 (19)	0.22671 (13)	0.16369 (14)	0.0494 (5)
H9A	0.8360	0.1943	0.2258	0.059*

H9B	0.8584	0.2081	0.1325	0.059*
C10	0.8280 (2)	0.31967 (14)	0.18801 (14)	0.0513 (5)
H10A	0.8025	0.3516	0.1269	0.062*
H10B	0.9337	0.3290	0.2361	0.062*
C11	0.73650 (19)	0.35231 (13)	0.23163 (13)	0.0484 (5)
H11A	0.7513	0.4138	0.2426	0.058*
H11B	0.7696	0.3250	0.2966	0.058*
C12	0.57270 (18)	0.33383 (12)	0.16183 (12)	0.0391 (4)
H12A	0.5162	0.3525	0.1935	0.047*
H12B	0.5373	0.3664	0.0998	0.047*
C13	0.28261 (16)	0.27120 (10)	−0.05590 (11)	0.0281 (4)
C14	0.30042 (18)	0.23330 (11)	−0.13061 (11)	0.0337 (4)
H14	0.3483	0.1802	−0.1182	0.040*
C15	0.24984 (19)	0.27151 (12)	−0.22198 (11)	0.0372 (4)
H15	0.2631	0.2447	−0.2713	0.045*
C16	0.18038 (17)	0.34834 (12)	−0.24104 (11)	0.0381 (4)
H16	0.1451	0.3745	−0.3037	0.046*
C17	0.16179 (17)	0.38764 (12)	−0.16865 (11)	0.0354 (4)
H17	0.1148	0.4411	−0. 820	0.042*
C18	0.21133 (16)	0.34971 (11)	−0.07617 (11)	0.0292 (4)
C19	0.19379 (16)	0.39796 (10)	−0.00022 (11)	0.0296 (4)
C20	0.27471 (16)	0.47027 (11)	0.03913 (11)	0.0307 (4)
C21	0.4527 (2)	0.56590 (13)	0.09150 (13)	0.0442 (5)
H21A	0.5460	0.5406	0.1434	0.053*
H21B	0.4761	0.6165	0.0650	0.053*
C22	0.26977 (18)	0.51901 (11)	0.11019 (12)	0.0356 (4)
C23	0.17869 (19)	0.49842 (12)	0.14608 (13)	0.0413 (4)

H23	0.1747	0.5312	0.1956	0.050*
C24	0.09231 (19)	0.42681 (12)	0.10581 (13)	0.0410 (4)
H24	0.0272	0.4113	0.1287	0.049*
C25	0.09581 (17)	0.37672 (11)	0.03403 (12)	0.0338 (4)
C26	−0.00685 (19)	0.30213 (12)	−0.00861 (13)	0.0414 (4)
H26A	0.0360	0.2535	0.0356	0.062*
H26B	−0.0211	0.2884	−0.0741	0.062*
H26C	−0.1021	0.3161	−0.0150	0.062*
O1	0.37159 (12)	0.50630 (8)	0.01358 (8)	0.0376 (3)
O2	0.36142 (13)	0.58823 (8)	0.13200 (8)	0.0435 (3)
P1	0.34252 (4)	0.21990 (3)	0.06512 (3)	0.02895 (13)

Atomic displacement parameters (Å^2)

	U^{11}	U^{22}	U^{33}	U^{12}	U^{13}	U^{23}
C1	0.0303 (8)	0.0336 (10)	0.0287 (7)	0.0025 (7)	0.0152 (7)	0.0009 (7)
C2	0.0358 (10)	0.0417 (11)	0.0376 (9)	−0.0048 (8)	0.0147 (8)	−0.0030 (8)
C3	0.0523 (12)	0.0451 (12)	0.0507 (11)	−0.0124 (10)	0.0237 (10)	−0.0088 (9)
C4	0.0671 (14)	0.0385 (12)	0.0606 (12)	−0.0075 (10)	0.0391 (11)	−0.0035 (10)
C5	0.0607 (13)	0.0375 (11)	0.0457 (10)	0.0090 (10)	0.0312 (10)	0.0101 (9)
C6	0.0420 (10)	0.0409 (11)	0.0332 (8)	0.0049 (9)	0.0193 (8)	0.0053 (8)
C7	0.0290 (9)	0.0397 (10)	0.0262 (7)	0.0016 (8)	0.0108 (7)	−0.0027 (7)
C8	0.0300 (9)	0.0483 (12)	0.0381 (9)	0.0009 (8)	0.0153 (8)	−0.0082 (8)

C9	0.0286 (10)	0.0670 (15)	0.0466 (10)	0.0037 (9)	0.0145 (8)	−0.0068 (10)
C10	0.0294 (10)	0.0677 (15)	0.0477 (10)	−0.0090 (10)	0.0127 (8)	−0.0082 (10)
C11	0.0401 (11)	0.0517 (13)	0.0426 (10)	−0.0085 (9)	0.0128 (8)	−0.0117 (9)
C12	0.0351 (9)	0.0430 (11)	0.0351 (8)	0.0001 (8)	0.0147 (8)	−0.0040 (8)
C13	0.0213 (8)	0.0352 (10)	0.0259 (7)	−0.0023 (7)	0.0106 (6)	−0.0004 (7)
C14	0.0322 (9)	0.0392 (11)	0.0306 (8)	−0.0001 (8)	0.0165 (7)	−0.0002 (7)
C15	0.0354 (9)	0.0499 (12)	0.0269 (8)	−0.0060 (9)	0.0161 (7)	−0.0042 (8)
C16	0.0305 (9)	0.0518 (12)	0.0276 (8)	−0.0038 (8)	0.0112 (7)	0.0074 (8)
C17	0.0275 (9)	0.0410 (11)	0.0337 (8)	0.0006 (8)	0.0125 (7)	0.0064 (8)
C18	0.0194 (8)	0.0365 (10)	0.0291 (7)	−0.0035 (7)	0.0103 (6)	0.0000 (7)
C19	0.0238 (8)	0.0331 (10)	0.0303 (8)	0.0050 (7)	0.0124 (7)	0.0056 (7)
C20	0.0248 (8)	0.0351 (10)	0.0322 (8)	0.0032 (7)	0.0145 (7)	0.0046 (7)
C21	0.0405 (11)	0.0463 (12)	0.0442 (10)	−0.0097 (9)	0.0202 (9)	−0.0059 (9)
C22	0.0319 (9)	0.0341 (10)	0.0361 (8)	0.0026 (8)	0.0137 (7)	0.0011 (8)
C23	0.0428 (10)	0.0456 (12)	0.0403 (9)	0.0070 (9)	0.0244 (8)	−0.0021 (8)
C24	0.0356 (10)	0.0489 (12)	0.0473 (10)	0.0067 (9)	0.0274 (8)	0.0071 (9)
C25	0.0264 (8)	0.0360 (10)	0.0390 (8)	0.0047 (7)	0.0165 (7)	0.0057 (8)
C26	0.0318 (9)	0.0452 [illegible]	0.0513 [illegible]	0.0000 (8)	0.0240 (8)	0.0047 (9)

		(12)	(10)			
O1	0.0338 (7)	0.0412 (8)	0.0391 (6)	−0.0085 (5)	0.0193 (5)	−0.0038 (5)
O2	0.0429 (7)	0.0393 (8)	0.0456 (7)	−0.0033 (6)	0.0204 (6)	−0.0067 (6)
P1	0.0281 (2)	0.0341 (3)	0.0249 (2)	0.00224 (19)	0.01351 (17)	0.00072 (17)

Geometric parameters (Å, °)

C1—C6	1.533 (2)	C11—H11B	0.9900
C1—C2	1.536 (2)	C12—H12A	0.9900
C1—P1	1.8570 (17)	C12—H12B	0.9900
C1—H1	1.0000	C13—C18	1.405 (2)
C2—C3	1.529 (3)	C13—C14	1.406 (2)
C2—H2A	0.9900	C13—P1	1.8514 (15)
C2—H2B	0.9900	C14—C15	1.386 (2)
C3—C4	1.516 (3)	C14—H14	0.9500
C3—H3A	0.9900	C15—C16	1.374 (3)
C3—H3B	0.9900	C15—H15	0.9500
C4—C5	1.522 (3)	C16—C17	1.388 (2)
C4—H4A	0.9900	C16—H16	0.9500
C4—H4B	0.9900	C17—C18	1.400 (2)
C5—C6	1.522 (3)	C17—H17	0.9500
C5—H5A	0.9900	C18—C19	1.498 (2)
C5—H5B	0.9900	C19—C20	1.375 (2)
C6—H6A	0.9900	C19—C25	1.419 (2)
C6—H6B	0.9900	C20—C22	1.374 (2)
C7—C12	1.525 (2)	C20—O1	1.3898 (18)
C7—C8	1.533 (2)	C21—O2	1.433 (2)

C7—P1	1.8621 (16)	C21—O1	1.434 (2)
C7—H7	1.0000	C21—H21A	0.9900
C8—C9	1.528 (2)	C21—H21B	0.9900
C8—H8A	0.9900	C22—C23	1.370 (2)
C8—H8B	0.9900	C22—O2	1.386 (2)
C9—C10	1.514 (3)	C23—C24	1.391 (3)
C9—H9A	0.9900	C23—H23	0.9500
C9—H9B	0.9900	C24—C25	1.389 (2)
C10—C11	1.521 (3)	C24—H24	0.9500
C10—H10A	0.9900	C25—C26	1.512 (2)
C10—H10B	0.9900	C26—H26A	0.9800
C11—C12	1.532 (2)	C26—H26B	0.9800
C11—H11A	0.9900	C26—H26C	0.9800
C6—C1—C2	109.84 (14)	C10—C11—H11B	109.5
C6—C1—P1	110.81 (11)	C12—C11—H11B	109.5
C2—C1—P1	109.28 (11)	H11A—C11—H11B	108.1
C6—C1—H1	109.0	C7—C12—C11	111.87 (15)
C2—C1—H1	109.0	C7—C12—H12A	109.2
P1—C1—H1	109.0	C11—C12—H12A	109.2
C3—C2—C1	111.85 (15)	C7—C12—H12B	109.2
C3—C2—H2A	109.2	C11—C12—H12B	109.2
C1—C2—H2A	109.2	H12A—C12—H12B	107.9
C3—C2—H2B	109.2	C18—C13—C14	118.10 (14)
C1—C2—H2B	109.2	C18—C13—P1	119.23 (11)
H2A—C2—H2B	107.9	C14—C13—P1	122.62 (12)
C4—C3—C2	110.82 (15)	C15—C14—C13	121.60 (16)
C4—C3—H3A	109.5	C15—C14—H14	119.2

C2—C3—H3A	109.5	C13—C14—H14	119.2
C4—C3—H3B	109.5	C16—C15—C14	119.84 (15)
C2—C3—H3B	109.5	C16—C15—H15	120.1
H3A—C3—H3B	108.1	C14—C15—H15	120.1
C3—C4—C5	111.33 (16)	C15—C16—C17	119.98 (15)
C3—C4—H4A	109.4	C15—C16—H16	120.0
C5—C4—H4A	109.4	C17—C16—H16	120.0
C3—C4—H4B	109.4	C16—C17—C18	120.96 (17)
C5—C4—H4B	109.4	C16—C17—H17	119.5
H4A—C4—H4B	108.0	C18—C17—H17	119.5
C6—C5—C4	111.65 (15)	C17—C18—C13	119.52 (14)
C6—C5—H5A	109.3	C17—C18—C19	117.76 (15)
C4—C5—H5A	109.3	C13—C18—C19	122.61 (13)
C6—C5—H5B	109.3	C20—C19—C25	115.64 (14)
C4—C5—H5B	109.3	C20—C19—C18	119.01 (13)
H5A—C5—H5B	108.0	C25—C19—C18	125.33 (15)
C5—C6—C1	111.66 (14)	C22—C20—C19	124.32 (14)
C5—C6—H6A	109.3	C22—C20—O1	109.32 (15)
C1—C6—H6A	109.3	C19—C20—O1	126.35 (14)
C5—C6—H6B	109.3	O2—C21—O1	107.06 (13)
C1—C6—H6B	109.3	O2—C21—H21A	110.3
H6A—C6—H6B	107.9	O1—C21—H21A	110.3
C12—C7—C8	110.48 (14)	O2—C21—H21B	110.3
C12—C7—P1	109.82 (11)	O1—C21—H21B	110.3
C8—C7—P1	117.81 (11)	H21A—C21—H21B	108.6
C12—C7—H7	106.0	C23—C22—C20	120.92 (17)
C8—C7—H7	106.0	C23—C22—O2	129.12 (16)

P1—C7—H7	106.0	C20—C22—O2	109.90 (14)
C9—C8—C7	110.94 (14)	C22—C23—C24	116.20 (16)
C9—C8—H8A	109.5	C22—C23—H23	121.9
C7—C8—H8A	109.5	C24—C23—H23	121.9
C9—C8—H8B	109.5	C25—C24—C23	123.73 (16)
C7—C8—H8B	109.5	C25—C24—H24	118.1
H8A—C8—H8B	108.0	C23—C24—H24	118.1
C10—C9—C8	111.48 (16)	C24—C25—C19	119.14 (16)
C10—C9—H9A	109.3	C24—C25—C26	120.01 (15)
C8—C9—H9A	109.3	C19—C25—C26	120.84 (15)
C10—C9—H9B	109.3	C25—C26—H26A	109.5
C8—C9—H9B	109.3	C25—C26—H26B	109.5
H9A—C9—H9B	108.0	H26A—C26—H26B	109.5
C9—C10—C11	111.35 (16)	C25—C26—H26C	109.5
C9—C10—H10A	109.4	H26A—C26—H26C	109.5
C11—C10—H10A	109.4	H26B—C26—H26C	109.5
C9—C10—H10B	109.4	C20—O1—C21	103.67 (12)
C11—C10—H10B	109.4	C22—O2—C21	103.43 (13)
H10A—C10—H10B	108.0	C13—P1—C1	101.39 (7)
C10—C11—C12	110.70 (14)	C13—P1—C7	103.63 (7)
C10—C11—H11A	109.5	C1—P1—C7	103.51 (7)
C12—C11—H11A	109.5		
C6—C1—C2—C3	−55.59 (18)	C25—C19—C20—O1	−176.40 (14)
P1—C1—C2—C3	−177.35 (12)	C18—C19—C20—O1	1.9 (2)
C1—C2—C3—C4	56.3 (2)	C19—C20—C22—C23	−1.2 (3)
C2—C3—C4—C5	−55.5 (2	O1—C20—C22—C23	178.08 (15)
C3—C4—C5—C6	55.4 (2)	C19—C20—C22—O2	−178.57 (15)

C4—C5—C6—C1	−55.43 (19)	O1—C20—C22—O2	0.66 (18)
C2—C1—C6—C5	54.95 (18)	C20—C22—C23—C24	−0.6 (2)
P1—C1—C6—C5	175.79 (12)	O2—C22—C23—C24	176.26 (16)
C12—C7—C8—C9	55.3 (2)	C22—C23—C24—C25	0.7 (3)
P1—C7—C8—C9	−177.40 (13)	C23—C24—C25—C19	1.0 (3)
C7—C8—C9—C10	−56.1 (2)	C23—C24—C25—C26	−177.37 (17)
C8—C9—C10—C11	56.3 (2)	C20—C19—C25—C24	−2.5 (2)
C9—C10—C11—C12	−55.5 (2)	C18—C19—C25—C24	179.26 (15)
C8—C7—C12—C11	−55.44 (18)	C20—C19—C25—C26	175.79 (15)
P1—C7—C12—C11	172.96 (11)	C18—C19—C25—C26	−2.4 (2)
C10—C11—C12—C7	55.5 (2)	C22—C20—O1—C21	14.92 (17)
C18—C13—C14—C15	0.1 (2)	C19—C20—O1—C21	−165.87 (16)
P1—C13—C14—C15	177.59 (12)	O2—C21—O1—C20	−24.76 (17)
C13—C14—C15—C16	0.0 (3)	C23—C22—O2—C21	166.93 (18)
C14—C15—C16—C17	0.3 (3)	C20—C22—O2—C21	−15.93 (17)
C15—C16—C17—C18	−0.8 (2)	O1—C21—O2—C22	25.09 (17)
C16—C17—C18—C13	0.8 (2)	C18—C13—P1—C1	150.44 (12)
C16—C17—C18—C19	177.09 (14)	C14—C13—P1—C1	−27.07 (15)
C14—C13—C18—C17	−0.5 (2)	C18—C13—P1—C7	−102.46 (13)
P1—C13—C18—C17	−178.08 (11)	C14—C13—P1—C7	80.03 (14)
C14—C13—C18—C19	−176.54 (14)	C6—C1—P1—C13	170.31 (11)
P1—C13—C18—C19	5.8 (2)	C2—C1—P1—C13	−68.52 (12)
C17—C18—C19—C20	−67.27 (19)	C6—C1—P1—C7	63.11 (12)
C13—C18—C19—C20	108.87 (18)	C2—C1—P1—C7	−175.72 (11)
C17—C18—C19—C25	110.89 (18)	C12—C7—P1—C13	69.22 (12)
C13—C18—C19—C25	−73.0 (2)	C8—C7—P1—C13	−58.41 (15)
C25—C19—C20—C22	2.7 (2)	C12—C7—P1—C1	174.72 (10)

C18—C19—C20—C22	−178.96 (15)	C8—C7—P1—C1	47.09 (15)

All e.s.d.'s (except the e.s.d. in the dihedral angle between two l.s. planes) are estimated using the full covariance matrix. The cell e.s.d.'s are taken into account individually in the estimation of e.s.d.'s in distances, angles and torsion angles; correlations between e.s.d.'s in cell parameters are only used when they are defined by crystal symmetry. An approximate (isotropic) treatment of cell e.s.d.'s is used for estimating e.s.d.'s involving l.s. planes.

Computing details

Data collection: Collect (Nonius B.V., 1998); cell refinement: *DENZO* (Nonius B.V., 1998); data reduction: *DENZO* (Nonius B.V., 1998); program(s) used to solve structure: *SHELXS97* (Sheldrick, 1997); program(s) used to refine structure: *SHELXL97* (Sheldrick, 1997); molecular graphics: *PLATON* 98 (Spek, 1998); software used to prepare material for publication: *SHELXL97* (Sheldrick, 1997).
