# Supplementary material for: Bromine–lithium exchange: An efficient tool in the modular construction of biaryl ligands
Source: Beilstein J Org Chem. 2011 Sep 14;7:1278–87. doi: 10.3762/bjoc.7.148 (PMC3182437; doi:10.3762/bjoc.7.148)
Supplement: File 6 — Crystal structure data for 6c. [file Beilstein_J_Org_Chem-07-1278-s006.pdf]

Supporting Information

for

Bromine–lithium exchange: An efficient tool in the modular construction of biaryl ligands

Laurence Bonnafoux, Frédéric R. Leroux* and Françoise Colobert*

Address: Laboratoire de stéréochimie, UMR 7509, CNRS-Université de Strasbourg, ECPM, 25 rue Becquerel, F-67087 Strasbourg Cedex 02, France

Email: Laurence Bonnafoux - laurence.bonnafoux@gmail.com; Frédéric R. Leroux* - frederic.leroux@unistra.fr; Françoise Colobert* - francoise.colobert@unistra.fr

* Corresponding author

Crystal structure data for 6c.
CCDC 827190

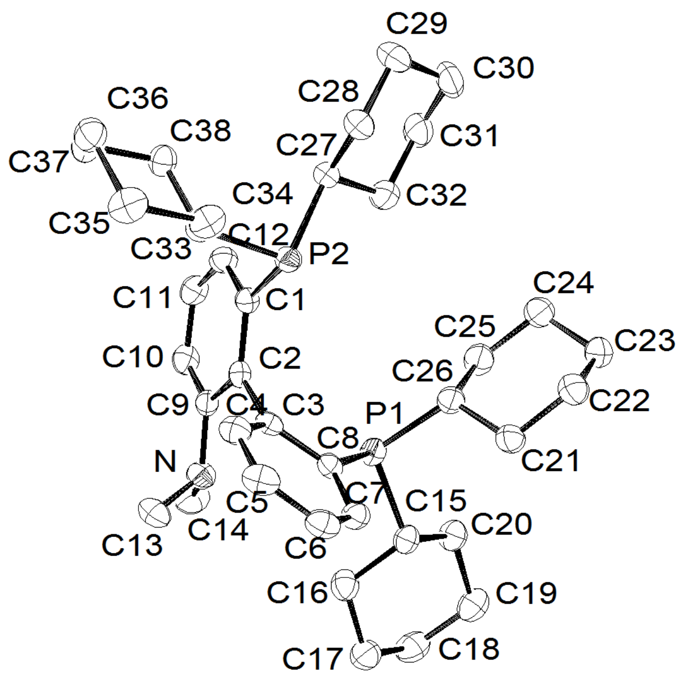

Crystal data

$C_{38}H_{57}NP_2$	
M_r = 589.79	D_x = 1.126 Mg m^{-3}
Monoclinic, $P2_1/c$	Melting point: ? K
Hall symbol: -P 2ybc	Mo *K*α radiation, λ = 0.71073 Å
a = 17.2800 (3) Å	Cell parameters from 9078 reflections
b = 10.9970 (2) Å	θ = 1.0–29.1°
c = 21.6860 (4) Å	μ = 0.15 mm^{-1}
β = 122.3851 (8)°	*T* = 173 K
V = 3480.01 (11) Å^3	Prism, colorless
Z = 4	0.18 × 0.14 × 0.14 mm
F(000) = 1288	

Data collection

KappaCCD diffractometer	5525 reflections with $I > 2\sigma(I)$
Radiation source: fine-focus sealed tube	R_{int} = 0.048

graphite	θ_{max} = 29.2°, θ_{min} = 1.4°
Detector resolution: ? pixels mm^{-1}	*h* = −23→23
π scans	*k* = −15→13
15417 measured reflections	*l* = −29→29
9362 independent reflections	

Refinement

Refinement on F^2	Secondary atom site location: difference Fourier map
Least-squares matrix: full	Hydrogen site location: inferred from neighbouring sites
$R[F^2 > 2\sigma(F^2)]$ = 0.050	H-atom parameters constrained
$wR(F^2)$ = 0.134	$w = 1/[\sigma^2(F_o^2) + (0.0629P)^2]$ where $P = (F_o^2 + 2F_c^2)/3$
S = 1.00	$(\Delta/\sigma)_{max}$ = 0.001
9362 reflections	$\Delta\rho_{max}$ = 0.29 e Å^{-3}
370 paramet rs	$\Delta\rho_{min}$ = −0.36 e Å^{-3}
0 restraints	Extinction correction: none
Primary atom site location: structure-invariant direct methods	

Refinement of F^2 against ALL reflections. The weighted *R*-factor *wR* and goodness of fit S are based on F^2, conventional *R*-factors *R* are based on F, with F set to zero for negative F^2. The threshold expression of F^2 > 2sigma(F^2) is used only for calculating *R*-factors(gt), etc. and is not relevant to the choice of reflections for refinement. *R*-factors based on F^2 are statistically about twice as large as those based on F, and *R*– factors based on ALL data will be even larger.

Fractional atomic coordinates and isotropic or equivalent isotropic displacement parameters (Å^2)

	x	*y*	*z*	U_{iso}*/U_{eq}
P1	0.91843 (3)	0.14246 (4)	0.65350 (2)	0.02714 (12)
P2	0.60987 (3)	0.16517 (4)	0.46461 (2)	0.02599 (12)

N	0.93937 (9)	0.24522 (14)	0.51579 (8)	0.0307 (4)
C1	0.70170 (10)	0.26860 (16)	0.47772 (9)	0.0245 (4)
C2	0.78540 (10)	0.21866 (16)	0.49289 (8)	0.0236 (4)
C3	0.80320 (10)	0.08453 (16)	0.50451 (9)	0.0239 (4)
C4	0.75907 (11)	0.00450 (18)	0.44538 (9)	0.0306 (4)
H4	0.7165	0.0360	0.3982	0.037*
C5	0.77563 (11)	−0.11862 (18)	0.45367 (10)	0.0343 (4)
H5	0.7443	−0.1712	0.4127	0.041*
C6	0.83822 (11)	−0.16567 (16)	0.52203 (10)	0.0317 (4)
H6	0.8505	−0.2505	0.5282	0.038*
C7	0.88271 (11)	−0.08784 (16)	0.58137 (9)	0.0287 (4)
H7	0.9259	−0.1205	0.6281	0.034*
C8	0.86593 (10)	0.03763 (16)	0.57454 (9)	0.0236 (4)
C9	0.85503 (10)	0.29652 (17)	0.49934 (9)	0.0254 (4)
C10	0.84011 (11)	0.42097 (18)	0.49296 (9)	0.0316 (4)
H10	0.8866	0.4735	0.4976	0.038*
C11	0.75913 (11)	0.47039 (17)	0.48001 (9)	0.0322 (4)
H11	0.7508	0.5561	0.4767	0.039*
C12	0.69042 (11)	0.39491 (17)	0.47189 (9)	0.0300 (4)
H12	0.6345	0.4293	0.4622	0.036*
C13	0.93793 (12)	0.16859 (19)	0.46058 (11)	0.0403 (5)
H13A	0.8849	0.1140	0.4398	0.060*
H13B	0.9944	0.1204	0.4829	0.060*
H13C	0.9335	0.2197	0.4218	0.060*
C14	1.01711 (12)	0.32833 (19)	0.54717 (11)	0.0420 (5)
H14A	1.0104	0.3838	0.5092	0.063*
H14B	1.0740	0.2817	0.5670	0.063*

H14C	1.0192	0.3754	0.5864	0.063*
C15	1.03232 (10)	0.07045 (16)	0.71507 (9)	0.0288 (4)
H15	1.0242	−0.0184	0.7197	0.035*
C16	1.08980 (11)	0.08864 (19)	0.68093 (10)	0.0353 (4)
H16A	1.0606	0.0440	0.6340	0.042*
H16B	1.0902	0.1761	0.6703	0.042*
C17	1.18859 (12)	0.04483 (19)	0.73010 (11)	0.0415 (5)
H17A	1.1890	−0.0446	0.7355	0.050*
H17B	1.2233	0.0645	0.7070	0.050*
C18	1.23517 (12)	0.1037 (2)	0.80495 (11)	0.0453 (5)
H18A	1.2420	0.1920	0.8003	0.054*
H18B	1.2972	0.0686	0.8366	0.054*
C19	1.17981 (12)	0.0837 (2)	0.83986 (10)	0.0438 (5)
H19A	1.2098	0.1266	0.8873	0.053*
H19B	1.1787	−0.0042	0.8493	0.053*
C20	1.08147 (12)	0.12988 (18)	0.79088 (10)	0.0353 (4)
H20A	1.0824	0.2192	0.7854	0.042*
H20B	1.0470	0.1124	0.8144	0.042*
C21	0.86550 (11)	−0.02207 (17)	0.72991 (10)	0.0331 (4)
H21A	0.9303	−0. 260	0.7709	0.040*
H21B	0.8554	−0.0873	0.6949	0.040*
C22	0.80336 (12)	−0.04428 (18)	0.75915 (10)	0.0372 (5)
H22A	0.7391	−0.0514	0.7176	0.045*
H22B	0.8207	−0.1220	0.7864	0.045*
C23	0.81042 (12)	0.05770 (19)	0.80896 (10)	0.0384 (5)
H23A	0.8735	0.0608	0.8526	0.046*
H23B	0.7678	0.0419	0.8254	0.046*

C24	0.78699 (13)	0.17862 (19)	0.76925 (10)	0.0394 (5)
H24A	0.7937	0.2444	0.8029	0.047*
H24B	0.7224	0.1774	0.7278	0.047*
C25	0.84945 (13)	0.20475 (19)	0.74069 (10)	0.0382 (5)
H25A	0.9131	0.2163	0.7826	0.046*
H25B	0.8296	0.2812	0.7122	0.046*
C26	0.84759 (11)	0.10080 (17)	0.69212 (9)	0.0286 (4)
H26	0.7829	0.0971	0.6493	0.034*
C27	0.54513 (11)	0.26195 (17)	0.49228 (9)	0.0288 (4)
H27	0.5271	0.3394	0.4637	0.035*
C28	0.45866 (12)	0.19574 (19)	0.47743 (10)	0.0370 (5)
H28A	0.4181	0.1799	0.4244	0.044*
H28B	0.4761	0.1164	0.5030	0.044*
C29	0.40661 (13)	0.2705 (2)	0.50328 (12)	0.0458 (5)
H29A	0.3837	0.3462	0.4742	0.055*
H29B	0.3531	0.2235	0.4952	0.055*
C30	0.46771 (15)	0.3025 (2)	0.58376 (12)	0.0496 (6)
H30A	0.4336	0.3561	0.5978	0.060*
H30B	0.4839	0.2273	0.6132	0.060*
C31	0.55485 (14)	0.3663 (2)	0.60044 (10)	0.0448 (5)
H31A	0.5951	0.3794	0.6538	0.054*
H31B	0.5392	0.4468	0.5763	0.054*
C32	0.60619 (12)	0.2915 (2)	0.57394 (10)	0.0388 (5)
H32A	0.6279	0.2148	0.6021	0.047*
H32B	0.6605	0.3376	0.5830	0.047*
C33	0.53870 (11)	0.16834 (16)	0.36168 (9)	0.0266 (4)
H33	0.5839	0.1666	0.3463	0.032*

C34	0.48207 (11)	0.05179 (17)	0.33029 (10)	0.0345 (4)
H34A	0.5227	−0.0198	0.3519	0.041*
H34B	0.4353	0.0479	0.3434	0.041*
C35	0.43479 (12)	0.04816 (18)	0.24727 (10)	0.0387 (5)
H35A	0.4817	0.0417	0.2345	0.046*
H35B	0.3952	−0.0248	0.2280	0.046*
C36	0.37714 (12)	0.16064 (18)	0.21178 (10)	0.0357 (4)
H36A	0.3261	0.1625	0.2201	0.043*
H36B	0.3504	0.1572	0.1584	0.043*
C37	0.43393 (11)	0.27612 (18)	0.24251 (10)	0.0345 (4)
H37A	0.3938	0.3480	0.2201	0.041*
H37B	0.4812	0.2783	0.2300	0.041*
C38	0.48031 (11)	0.28176 (17)	0.32547 (9)	0.0303 (4)
H38A	0.4330	0.2883	0.3378	0.036*
H38B	0.5196	0.3550	0.3445	0.036*

Atomic displacement parameters (Å^2)

	U^{11}	U^{22}	U^{33}	U^{12}	U^{13}	U^{23}
P1	0.0310 (2)	0.0232 (3)	0.0256 (2)	−0.00075 (19)	0.0141 (2)	−0.0011 (2)
P2	0.0234 (2)	0.0271 (3)	0.0287 (3)	0.00086 (18)	0.01474 (19)	0.0025 (2)
N	0.0244 (7)	0.0334 (9)	0.0354 (9)	−0.0037 (7)	0.0166 (7)	0.0008 (7)
C1	0.0247 (8)	0.0266 (10)	0.0225 (9)	0.0005 (7)	0.0129 (7)	−0.0003 (7)
C2	0.0262 (8)	0.0260 (10)	0.0189 (8)	−0.0015 (7)	0.0122 (7)	0.0003 (7)
C3	0.0202 (7)	0.0262 (10)	0.0298 (9)	−0.0018 (7)	0.0164 (7)	−0.0024 (8)

C4	0.0256 (8)	0.0327 (11)	0.0312 (10)	−0.0015 (8)	0.0136 (8)	−0. 055 (8)
C5	0.0299 (9)	0.0314 (11)	0.0393 (11)	−0.0057 (8)	0.0169 (8)	−0.0140 (9)
C6	0.0324 (9)	0.0236 (10)	0.0436 (11)	−0.0018 (8)	0.0233 (9)	−0.0049 (9)
C7	0.0305 (8)	0.0245 (10)	0.0331 (10)	0.0016 (8)	0.0184 (8)	0.0025 (8)
C8	0.0241 (8)	0.0241 (10)	0.0270 (9)	−0.0013 (7)	0.0166 (7)	−0.0007 (8)
C9	0.0239 (8)	0.0301 (10)	0.0218 (9)	−0.0005 (7)	0.0121 (7)	0.0026 (8)
C10	0.0299 (9)	0.0318 (11)	0.0323 (10)	−0.0077 (8)	0.0161 (8)	0.0017 (8)
C11	0.0359 (9)	0.0249 (10)	0.0325 (10)	−0.0017 (8)	0.0163 (8)	0.0012 (8)
C12	0.0305 (9)	0.0283 (11)	0.0310 (10)	0.0035 (8)	0.0164 (8)	0.0000 (8)
C13	0.0360 (10)	0.0483 (14)	0.0475 (12)	0.0019 (9)	0.0296 (9)	0.0007 (10)
C14	0.0281 (9)	0.0401 (13)	0.0544 (13)	−0.0055 (8)	0.0198 (9)	0.0066 (10)
C15	0.0314 (9)	0.0251 (10)	0.0284 (9)	−0.0019 (8)	0.0150 (8)	0.0011 (8)
C16	0.0332 (9)	0.0400 (12)	0.0328 (10)	−0.0034 (9)	0.0176 (8)	0.0010 (9)
C17	0.0358 (10)	0.0430 (13)	0.0490 (12)	0.0034 (9)	0.0249 (10)	0.0102 (10)
C18	0.0304 (9)	0.0463 (14)	0.0463 (12)	0.0010 (9)	0.0119 (9)	0.0056 (11)
C19	0.0378 (10)	0.0469 (14)	0.0335 (11)	0.0010 (10)	0.0103 (9)	0.0040 (10)
C20	0.0340 (9)	0.0384 (12)	0.0283 (10)	−0.0020 (8)	0.0131 (8)	−0.0021 (9)

C21	0.0366 (9)	0.0295 (11)	0.0383 (11)	0.0034 (8)	0.0234 (9)	0.0016 (9)
C22	0.0375 (10)	0.0370 (12)	0.0414 (11)	0.0005 (9)	0.0242 (9)	0.0030 (9)
C23	0.0372 (10)	0.0495 (14)	0.0303 (10)	0.0060 (9)	0.0192 (9)	0.0051 (10)
C24	0.0462 (11)	0.0422 (13)	0.0320 (11)	0.0101 (9)	0.0224 (9)	−0.0014 (9)
C25	0.0520 (11)	0.0299 (11)	0.0363 (11)	0.0064 (9)	0.0260 (10)	−0.0010 (9)
C26	0.0330 (9)	0.0253 (10)	0.0285 (9)	0.0026 (8)	0.0171 (8)	−0.0010 (8)
C27	0.0279 (8)	0.0335 (11)	0.0288 (10)	0.0056 (8)	0.0177 (8)	0.0052 (8)
C28	0.0327 (9)	0.0441 (13)	0.0401 (11)	0.0023 (9)	0.0233 (9)	0.0064 (10)
C29	0.0407 (10)	0.0558 (15)	0.0563 (13)	0.0082 (10)	0.0360 (10)	0.0081 (11)
C30	0.0692 (14)	0.0507 (15)	0.0550 (14)	0.0177 (11)	0.0506 (12)	0.0151 (11)
C31	0.0563 (12)	0.0491 (14)	0.0350 (11)	0.0111 (10)	0.0285 (10)	0.0027 (10)
C32	0.0375 (10)	0.0477 (13)	0.0305 (10)	0.0105 (9)	0.0177 (9)	0.0033 (9)
C33	0.0255 (8)	0.0252 (10)	0.0306 (9)	−0.0002 (7)	0.0160 (7)	0.0013 (8)
C34	0.0332 (9)	0.0264 (11)	0.0368 (11)	−0.0012 (8)	0.0141 (8)	−0.0011 (9)
C35	0.0371 (10)	0.0313 (12)	0.0387 (11)	−0.0027 (9)	0.0143 (9)	−0.0079 (9)
C36	0.0324 (9)	0.0414 (12)	0.0299 (10)	−0.0019 (9)	0.0144 (8)	−0.0012 (9)
C37	0.0364 (10)	0.0366 (12)	0.0278 (10)	−0.0019 (8)	0.0153 (8)	0.0022 (9)

C38	0.0347 (9)	0.0264 (10)	0.0298 (10)	−0.0003 (8)	0.0173 (8)	0.0023 (8)

Geometric parameters (Å, °)

P1—C8	1.8494 (17)	C21—C26	1.524 (3)
P1—C15	1.8622 (17)	C21—C22	1.531 (2)
P1—C26	1.8731 (16)	C21—H21A	0.9900
P2—C1	1.8465 (16)	C21—H21B	0.9900
P2—C27	1.8610 (17)	C22—C23	1.516 (3)
P2—C33	1.8856 (17)	C22—H22A	0.9900
N—C9	1.419 (2)	C22—H22B	0.9900
N—C13	1.453 (2)	C23—C24	1.517 (3)
N—C14	1.457 (2)	C23—H23A	0.9900
C1—C12	1.399 (2)	C23—H23B	0.9900
C1—C2	1.411 (2)	C24—C25	1.532 (3)
C2—C9	1.421 (2)	C24—H24A	0.9900
C2—C3	1.500 (2)	C24—H24B	0.9900
C3—C4	1.398 (2)	C25—C26	1.543 (3)
C3—C8	1.408 (2)	C25—H25A	0.9900
C4—C5	1.376 (3)	C25—H25B	0.9900
C4—H4	0.9500	C26—H26	1.0000
C5—C6	1.385 (3)	C27—C28	1.533 (2)
C5—H5	0.9500	C27—C32	1.534 (2)
C6—C7	1.386 (2)	C27—H27	1.0000
C6—H6	0.9500	C28—C29	1.530 (3)
C7—C8	1.401 (2)	C28—H28A	0.9900
C7—H7	0.9500	C28—H28B	0.9900

C9—C10	1.386 (3)	C29—C30	1.520 (3)
C10—C11	1.382 (2)	C29—H29A	0.9900
C10—H10	0.9500	C29—H29B	0.9900
C11—C12	1.380 (2)	C30—C31	1.518 (3)
C11—H11	0.9500	C30—H30A	0.9900
C12—H12	0.9500	C30—H30B	0.9900
C13—H13A	0.9800	C31—C32	1.529 (3)
C13—H13B	0.9800	C31—H31A	0.9900
C13—H13C	0.9800	C31—H31B	0.9900
C14—H14A	0.9800	C32—H32A	0.9900
C14—H14B	0.9800	C32—H32B	0.9900
C14—H14C	0.9800	C33—C38	1.530 (2)
C15—C20	1.535 (2)	C33—C34	1.533 (2)
C15—C16	1.537 (2)	C33—H33	1.0000
C15—H15	1.0000	C34—C35	1.528 (3)
C16—C17	1.528 (2)	C34—H34A	0.9900
C16—H16A	0.9900	C34—H34B	0.9900
C16—H16B	0.9900	C35—C36	1.514 (3)
C17—C18	1.517 (3)	C35—H35A	0.9900
C17—H17A	0.9900	C35—H35B	0.9900
C17—H17B	0.9900	C36—C37	1.523 (3)
C18—C19	1.521 (3)	C36—H36A	0.9900
C18—H18A	0.9900	C36—H36B	0.9900
C18—H18B	0.9900	C37—C38	1.529 (2)
C19—C20	1.530 (2)	C37—H37A	0.9900
C19—H19A	0.9900	C37—H37B	0.9900
C19—H19B	0.9900	C38—H38A	0.9900

C20—H20A	0.9900	C38—H38B	0.9900
C20—H20B	0.9900		
C8—P1—C15	101.82 (7)	C21—C22—H22A	109.3
C8—P1—C26	98.49 (7)	C23—C22—H22B	109.3
C15—P1—C26	105.56 (7)	C21—C22—H22B	109.3
C1—P2—C27	102.33 (8)	H22A—C22—H22B	107.9
C1—P2—C33	97.63 (7)	C22—C23—C24	110.42 (15)
C27—P2—C33	104.34 (7)	C22—C23—H23A	109.6
C9—N—C13	117.47 (14)	C24—C23—H23A	109.6
C9—N—C14	115.72 (15)	C22—C23—H23B	109.6
C13—N—C14	110.67 (14)	C24—C23—H23B	109.6
C12—C1—C2	118.90 (14)	H23A—C23—H23B	108.1
C12—C1—P2	122.08 (11)	C23—C24—C25	111.25 (15)
C2—C1—P2	119.02 (13)	C23—C24—H24A	109.4
C1—C2—C9	119.75 (16)	C25—C24—H24A	109.4
C1—C2—C3	120.93 (13)	C23—C24—H24B	109.4
C9—C2—C3	119.29 (13)	C25—C24—H24B	109.4
C4—C3—C8	119.04 (16)	H24A—C24—H24B	108.0
C4—C3—C2	120.19 (15)	C24—C25—C26	112.04 (16)
C8—C3—C2	120.75 (15)	C24—C25—H25A	109.2
C5—C4—C3	121.79 (16)	C26—C25—H25A	109.2
C5—C4—H4	119.1	C24—C25—H25B	109.2
C3—C4—H4	119.1	C26—C25—H25B	109.2
C4—C5—C6	119.75 (17)	H25A—C25—H25B	107.9
C4—C5—H5	120.1	C21—C26—C25	111.47 (14)
C6—C5—H5	120.1	C21—C26—P1	117.66 (11)
C5—C6—C7	119.35 (17)	C25—C26—P1	110.14 (12)

C5—C6—H6	120.3	C21—C26—H26	105.5
C7—C6—H6	120.3	C25—C26—H26	105.5
C6—C7—C8	121.97 (16)	P1—C26—H26	105.5
C6—C7—H7	119.0	C28—C27—C32	109.49 (14)
C8—C7—H7	119.0	C28—C27—P2	110.31 (13)
C7—C8—C3	118.08 (15)	C32—C27—P2	109.45 (11)
C7—C8—P1	123.01 (13)	C28—C27—H27	109.2
C3—C8—P1	118.86 (13)	C32—C27—H27	109.2
C10—C9—N	121.94 (14)	P2—C27—H27	109.2
C10—C9—C2	118.86 (14)	C29—C28—C27	111.46 (16)
N—C9—C2	119.12 (16)	C29—C28—H28A	109.3
C11—C10—C9	121.50 (15)	C27—C28—H28A	109.3
C11—C10—H10	119.2	C29—C28—H28B	109.3
C9—C10—H10	119.2	C27—C28—H28B	109.3
C12—C11—C10	119.82 (17)	H28A—C28—H28B	108.0
C12—C11—H11	120.1	C30—C29—C28	111.23 (16)
C10—C11—H11	120.1	C30—C29—H29A	109.4
C11—C12—C1	121.12 (15)	C28—C29—H29A	109.4
C11—C12—H12	119.4	C30—C29—H29B	109.4
C1—C12—H12	119.4	C28—C29—H29B	109.4
N—C13—H13A	109.5	H29A—C29—H29B	108.0
N—C13—H13B	109.5	C31—C30—C29	111.75 (15)
H13A—C13—H13B	109.5	C31—C30—H30A	109.3
N—C13—H13C	109.5	C29—C30—H30A	109.3
H13A—C13—H13C	109.5	C31—C30—H30B	109.3
H13B—C13—H13C	109.5	C29—C30—H30B	109.3
N—C14—H14A	109.5	H30A—C30—H30B	107.9

N—C14—H14B	109.5	C30—C31—C32	111.32 (18)
H14A—C14—H14B	109.5	C30—C31—H31A	109.4
N—C14—H14C	109.5	C32—C31—H31A	109.4
H14A—C14—H14C	109.5	C30—C31—H31B	109.4
H14B—C14—H14C	109.5	C32—C31—H31B	109.4
C20—C15—C16	109.32 (14)	H31A—C31—H31B	108.0
C20—C15—P1	110.67 (12)	C31—C32—C27	111.65 (14)
C16—C15—P1	107.86 (11)	C31—C32—H32A	109.3
C20—C15—H15	109.7	C27—C32—H32A	109.3
C16—C15—H15	109.7	C31—C32—H32B	109.3
P1—C15—H15	109.7	C27—C32—H32B	109.3
C17—C16—C15	113.06 (14)	H32A—C32—H32B	108.0
C17—C16—H16A	109.0	C38—C33—C34	111.41 (14)
C15—C16—H16A	109.0	C38—C33—P2	117.08 (12)
C17—C16—H16B	109.0	C34—C33—P2	111.51 (12)
C15—C16—H16B	109.0	C38—C33—H33	105.2
H16A—C16—H16B	107.8	C34—C33—H33	105.2
C18—C17—C16	111.50 (15)	P2—C33—H33	105.2
C18—C17—H17A	109.3	C35—C34—C33	110.88 (15)
C16—C17—H17A	109.3	C35—C34—H34A	109.5
C18—C17—H17B	109.3	C33—C34—H34A	109.5
C16—C17—H17B	109.3	C35—C34—H34B	109.5
H17A—C17—H17B	108.0	C33—C34—H34B	109.5
C17—C18—C19	110.87 (15)	H34A—C34—H34B	108.1
C17—C18—H18A	109.5	C36—C35—C34	111.63 (16)
C19—C18—H18A	109.5	C36—C35—H35A	109.3
C17—C18—H18B	109.5	C34—C35—H35A	109.3

C19—C18—H18B	109.5	C36—C35—H35B	109.3
H18A—C18—H18B	108.1	C34—C35—H35B	109.3
C18—C19—C20	111.55 (15)	H35A—C35—H35B	108.0
C18—C19—H19A	109.3	C35—C36—C37	111.37 (14)
C20—C19—H19A	109.3	C35—C36—H36A	109.4
C18—C19—H19B	109.3	C37—C36—H36A	109.4
C20—C19—H19B	109.3	C35—C36—H36B	109.4
H19A—C19—H19B	108.0	C37—C36—H36B	109.4
C19—C20—C15	111.95 (15)	H36A—C36—H36B	108.0
C19—C20—H20A	109.2	C36—C37—C38	110.95 (15)
C15—C20—H20A	109.2	C36—C37—H37A	109.4
C19—C20—H20B	109.2	C38—C37—H37A	109.4
C15—C20—H20B	109.2	C36—C37—H37B	109.4
H20A—C20—H20B	107.9	C38—C37—H37B	109.4
C26—C21—C22	112.25 (14)	H37A—C37—H37B	108.0
C26—C21—H21A	109.2	C37—C38—C33	110.89 (15)
C22—C21—H21A	109.2	C37—C38—H38A	109.5
C26—C21—H21B	109.2	C33—C38—H38A	109.5
C22—C21—H21B	109.2	C37—C38—H38B	109.5
H21A—C21—H21B	107.9	C33—C38—H38B	109.5
C23—C22—C21	111.75 (15)	H38A—C38—H38B	108.0
C23—C22—H22A	109.3		
C27—P2—C1—C12	−28. 1 (16)	C20—C15—C16—C17	53.7 (2)
C33—P2—C1—C12	78.55 (15)	P1—C15—C16—C17	174.15 (14)
C27—P2—C1—C2	153.08 (13)	C15—C16—C17—C18	−54.8 (2)
C33—P2—C1—C2	−100.37 (13)	C16—C17—C18—C19	54.6 (2)
C12—C1—C2—C9	−2.2 (2)	C17—C18—C19—C20	−55.9 (2)

P2—C1—C2—C9	176.71 (12)	C18—C19—C20—C15	56.7 (2)
C12—C1—C2—C3	175.97 (15)	C16—C15—C20—C19	−54.4 (2)
P2—C1—C2—C3	−5.1 (2)	P1—C15—C20—C19	−173.02 (12)
C1—C2—C3—C4	73.16 (19)	C26—C21—C22—C23	−54.6 (2)
C9—C2—C3—C4	−108.63 (17)	C21—C22—C23—C24	57.24 (19)
C1—C2—C3—C8	−108. 3 (17)	C22—C23—C24—C25	−57.4 (2)
C9—C2—C3—C8	69.88 (19)	C23—C24—C25—C26	54.9 (2)
C8—C3—C4—C5	−0.4 (2)	C22—C21—C26—C25	51.2 (2)
C2—C3—C4—C5	178.16 (15)	C22—C21—C26—P1	179.75 (12)
C3—C4—C5—C6	−0.6 (3)	C24—C25—C26—C21	−51.5 (2)
C4—C5—C6—C7	0.6 (2)	C24—C25—C26—P1	176.00 (12)
C5—C6—C7—C8	0.4 (2)	C8—P1—C26—C21	72.34 (14)
C6—C7—C8—C3	−1.4 (2)	C15—P1—C26—C21	−32.52 (15)
C6—C7—C8—P1	175.90 (12)	C8—P1—C26—C25	−158.44 (12)
C4—C3—C8—C7	1.4 (2)	C15—P1—C26—C25	96.70 (13)
C2—C3—C8—C7	−177.17 (13)	C1—P2—C27—C28	174.01 (12)
C4—C3—C8—P1	−176.06 (11)	C33—P2—C27—C28	72.70 (13)
C2—C3—C8—P1	5.42 (18)	C1—P2—C27—C32	−65.46 (14)
C15—P1—C8—C7	33.38 (14)	C33—P2—C27—C32	−166.77 (12)
C26—P1—C8—C7	−74.57 (14)	C32—C27—C28—C29	56.6 (2)
C15—P1—C8—C3	−149.34 (11)	P2—C27—C28—C29	177.08 (12)
C26—P1—C8—C3	102.71 (12)	C27—C28—C29—C30	−56.2 (2)
C13—N—C9—C10	−115.67 (19)	C28—C29—C30—C31	54.6 (2)
C14—N—C9—C10	18.1 (2)	C29—C30—C31—C32	−54.3 (2)
C13—N—C9—C2	67.6 (2)	C30—C31—C32—C27	55.7 (2)
C14—N—C9—C2	−158.57 (15)	C28—C27—C32—C31	−56.4 (2)
C1—C2—C9—C10	2.2 (2)	P2—C27—C32—C31	−177.40 (14)

C3—C2—C9—C10	−176.07 (14)	C1—P2—C33—C38	−76.41 (13)
C1—C2—C9—N	178.97 (14)	C27—P2—C33—C38	28.46 (14)
C3—C2—C9—N	0.7 (2)	C1—P2—C33—C34	153.64 (12)
N—C9—C10—C11	−177.19 (16)	C27—P2—C33—C34	−101[illegible]49 (12)
C2—C9—C10—C11	−0.5 (3)	C38—C33—C34—C35	54.68 (18)
C9—C10—C11—C12	−1.1 (3)	P2—C33—C34—C35	−172.46 (11)
C10—C11—C12—C1	1.1 (3)	C33—C34—C35—C36	−54.95 (19)
C2—C1—C12—C11	0.6 (2)	C34—C35—C36—C37	56.0 (2)
P2—C1—C12—C11	−178.29 (13)	C35—C36—C37—C38	−56.29 (1[illegible])
C8—P1—C15—C20	−169.54 (12)	C36—C37—C38—C33	55.88 (18)
C26—P1—C15—C20	−67.14 (13)	C34—C33—C38—C37	−55.37 (18)
C8—P1—C15—C16	70.91 (13)	P2—C33—C38—C37	174.62 (11)
C26—P1—C15—C16	173.31 (12)		

All e.s.d.'s (except the e.s.d. in the dihedral angle between two l.s. planes) are estimated using the full covariance matrix. The cell e.s.d.'s are taken into account individually in the estimation of e.s.d.'s in distances, angles and torsion angles; correlations between e.s.d.'s in cell parameters are only used when they are defined by crystal symmetry. An approximate (isotropic) treatment of cell e.s.d.'s is used for estimating e.s.d.'s involving l.s. planes.

Refinement

(type here to add refinement details)

Computing details

Data collection: Collect (Nonius B.V., 1998); cell refinement: *DENZO* (Nonius B.V., 1998); data reduction: *DENZO* (Nonius B.V., 1998); program(s) used to solve structure: *SHELXS97* (Sheldrick, 1997); program(s) used to refine structure: *SHELXL97* (Sheldrick, 1997); molecular graphics: *PLATON* 98 (Spek, 1998); software used to prepare material for publication: *SHELXL97* (Sheldrick, 1997).

References

Burla, M. C., Camalli, M., Cascarano, G., Giacovazzo, C., Polidori, G., Spagna, R. & &Viterbo, D. (1989). *SIR*. J. Appl. Cryst. 22, 389-393.

A.L. Spek, *PLATON* 98, Utrecht University, The Netherland, 1998.

KappaCCD Operation Manual (1997). Nonius B.V., Delft, The Nederlands.

Otwinowski, Z. & Minor, W. (1997). *Methods in Enzymology*, **276**, 307–326.

Sheldrick, G. M. (1997). *SHELXL97*. Program for the refinement of crystal structures. University of Gottingen. Germany.
